# Factors Associated With Menopause Symptoms: A Systematic Review and Meta‐Analysis

**DOI:** 10.1111/1471-0528.70257

**Published:** 2026-05-06

**Authors:** Janice Hoang, Kathryn Halliday, Deborah Allen, Wema Meranda Mtika, Eve Tranter, Grace Glover, Lynn Tatnell, Julia Hippisley‐Cox, Carol Coupland, Sarah Hillman, Jennifer Hirst

**Affiliations:** ^1^ Nuffield Department of Primary Care Health Sciences University of Oxford Oxford UK; ^2^ Cancer Division Leeds Institute of Clinical Trial Research, University of Leeds Leeds UK; ^3^ Wolfson Institute of Population Health, Queen Mary University of London London UK; ^4^ Faculty of Medicine & Health Sciences University of Nottingham Nottingham UK; ^5^ Department of Applied Health, School of Health Sciences University of Birmingham Birmingham UK

**Keywords:** disparities, menopause, meta‐analysis, symptoms, systematic review

## Abstract

**Background:**

Menopause, marked by hormonal decline and menstrual cessation, is associated with various symptoms. Socio‐demographic and behavioural factors may influence symptom type and severity. Understanding these associations can inform better symptom management.

**Objectives:**

To identify factors associated with the presence and severity of menopausal symptoms through systematic review and meta‐analysis.

**Search Strategy:**

We searched Medline, Embase, CINAHL and Cochrane for studies on demographic, behavioural, or health factors linked to vasomotor, vaginal dryness and joint symptoms in women aged 40–60.

**Selection Criteria:**

Studies reporting odds ratios or raw numbers for symptom presence or severity were included.

**Data Collection and Analysis:**

Studies were combined for meta‐analysis, reporting odds ratios and 95% confidence intervals. Quality assessment was performed to quantify the risk of bias.

**Results:**

Of 9228 screened articles, 61 were meta‐analysed. Compared with White women, Black women had higher odds of vasomotor symptom presence (OR 1.65, 1.41–1.94) and severity (OR 1.91, 1.10–3.29), and vaginal dryness presence (OR 1.27, 1.10–1.47), while Asian had lower vasomotor symptom presence and severity (OR 0.40, 0.22–0.72; OR 0.55, 0.53–0.56). Higher education (OR 1.31, 1.09–1.56), high income (OR 1.41, 1.01–1.97) and depression (OR 2.36, 1.51–3.70) were associated with increased presence of vasomotor symptoms. Smoking and obesity were associated with both presence (OR 1.63, 1.30–2.04 and 1.35, 1.02–1.78) and severity (OR 1.56, 1.07–2.27 and 1.42, 1.11–1.83) of vasomotor symptoms.

**Conclusion:**

Socio‐demographic and behavioural factors, including ethnicity, education, income, smoking, obesity and depression, influence menopausal symptoms, highlighting the need for personalised care.

**Trial Registration:**

PROSPERO number: CRD42023459154

## 1 Introduction

Menopause is a natural phase of aging experienced by women and people with ovaries, marked by the end of menstrual cycles and a change in hormone levels. The menopausal transition usually takes place between the ages of 40 and 60, where the ovaries produce progressively less oestrogen, which causes symptoms. These hormonal changes are linked to a range of menopausal symptoms [1, 2, 3, 4]. Most women experience vasomotor symptoms (VMS), including hot flashes and night sweats, with VMS lasting a median of 7.4 years and often having a profound impact on quality of life [3].

Prior research highlights that individual socio‐demographic characteristics might be associated with the experience of menopause symptoms [5, 6, 7]. The Study of Women's Health Across the Nation (SWAN) in the United States observed that vasomotor symptoms were more common in African American and Hispanic women compared to White women and found that low‐income women reported more frequent and severe menopausal symptoms than those with higher incomes [6] A recent study highlighted that women from ethnic minorities and more deprived backgrounds may experience a longer duration or severity of VMS [8]. Many studies have sought to describe the relationship between race/ethnicity and specific symptoms, but no study to date combined data from all published studies [7, 9, 10, 11, 12]. However, these findings should be interpreted with caution because they may be influenced by multi‐factorial reasons such as access to healthcare and the use of hormone therapy, which can vary across different socio‐demographic groups and countries [13, 14].

Therefore, to gain a comprehensive understanding of how individual characteristics influence menopause symptoms, we conducted a systematic review to identify socioeconomic, health‐related and behavioural factors associated with the presence and severity of vasomotor, vaginal dryness, and joint symptoms during the menopause from published data.

## 2 Methods

### 2.1 Search Strategy and Selection Criteria

The study protocol was registered in the PROSPERO database, registration number CRD42023459154 (www.crd.york.ac.uk). Searches were performed on 10th October 2023 of Medline, Embase, CINAHL and Cochrane databases and citation searching to identify relevant studies. Searches were restricted to articles published from 1980 to 2023, using search terms related to menopause, symptoms, severity, prevalence and risk factors (Table S1). Relevant articles were imported into Covidence [13] for screening.

### 2.2 Inclusion and Exclusion Criteria

Observational studies written in any language investigating factors associated with the severity or presence of menopausal symptoms in women in the age range 40–60 years or an age group overlapping with this were included. We included studies reporting quantitative associations between sociodemographic, health and behavioural factors and the presence or severity of menopausal symptoms. Studies in other languages were translated into English using Google translate. Exclusion criteria were: studies restricted to women who had gone through premature or surgical menopause; women with current or previous cancer; life‐limiting conditions, or health conditions related to menstruation, such as endometriosis or polycystic ovarian syndrome.

### 2.3 Study Screening and Data Extraction

Studies were screened in duplicate by six authors (JH, ET, GG, DA, WMM, JAH) at two stages: title and abstract, then full text. Where conflicts arose, these were discussed and resolved between at least three reviewers. Data were manually extracted from eligible articles into a piloted data extraction form in Microsoft Excel (Microsoft Corporation, Version 2408): first author and year; study title; journal and citation; country; study design; study period; study setting; exposures; outcomes; statistics reported; sample size; and study population age.

Exposures included (i) demographic factors: income, education, ethnicity, occupation/employment, (ii) behavioural factors: physical activity, smoking, alcohol consumption, marital status, body mass index (BMI), number of children, (iii) comorbidities: chronic disease, hypertension, diabetes, self‐reported health status, anxiety or pre‐existing depression (as opposed to depression attributed to menopause), hyperlipidaemia, sleep disturbance, joint pain, comorbidities, acute myocardial infarction.

Outcomes were menopause symptoms. Due to a large number of outcomes reported in included papers, in consultation with an academic GP specialising in women's health, a post hoc decision was made to limit the outcomes assessed to outcomes that were more consistently reported across studies. These were: presence and severity of vasomotor, genitourinary (including vaginal dryness, urinary tract infections, urine leakage, vaginal dryness, vaginal pain, dyspareunia, vaginal itching, vaginal discharge, vaginal burning and postcoital bleeding), and joint symptoms (pain/stiffness). Adjusted and unadjusted odds ratios and raw numbers for each menopause symptom were extracted. Articles only reporting *p*‐values or correlation coefficients were excluded from meta‐analysis.

### 2.4 Handling of Randomised Controlled Trials (RCTs)

Randomised controlled trials were included only when they reported observational associations between non‐randomised exposures (e.g., BMI, ethnicity, smoking, education) and menopausal symptoms within the trial cohort. Randomised treatment effects were not analysed. Eligible RCTs were treated as prospective cohort datasets, and only baseline or pre‐intervention exposure–symptom associations were extracted. Any estimate that could plausibly be influenced by the randomised intervention (e.g., hormone therapy or symptom‐modifying treatments) was excluded.

### 2.5 Quality Assessment

We developed an assessment tool for evaluating risk of bias for each paper by combining domains from the QUIPS Tool [14] and Newcastle‐Ottawa Scale [15]. Our study examines multiple factors associated with each outcome; therefore, we used the Newcastle–Ottawa Scale (NOS), which assesses the quality of non‐randomised cohort and case–control studies across selection, comparability and outcome (or exposure) domains. However, the NOS has been criticised for limited guidance and insufficient assessment of confounding and analytical methods. To address these limitations, we incorporated relevant domains from the QUIPS tool, which offers stronger evaluation of confounding, adjustment for other factors, and statistical reporting. Integrating both frameworks enabled a more comprehensive assessment of study quality.

Our adapted tool consisted of six items: *(i) study population inclusion and exclusion criteria*, *(ii) definition of exposures and methods of measurement*, *(iii) definition of outcomes and methods of measurement*, *(iv) measurement of comparison among exposures*, *(v) factors addressing study confounding* and *(vi) statistical analysis and reporting*. Elements of these domains were rated as ‘1‐low’, ‘2‐moderate’, ‘3‐high or unclear’. Total quality scores were categorised as follows: ≤ 22 = low risk, 23–43 = moderate risk and ≥ 44 = high risk, to reflect the overall assessment of each study. This adapted tool has been previously used [16]. Quality assessment for each paper was undertaken by DA, KH and JH independently, and mean scores from reviewers were calculated to reduce individual bias.

### 2.6 Defining Exposure and Outcome Variables

Exposures were simplified into binary variables representing presence or absence, or high or low levels, using absence or low as reference groups. We compared Black and White ethnicities for the main analysis, and ‘any other ethnicities’ compared to White ethnicity as secondary analysis because studies reported varying ethnic categories. The number of participants in the White comparator arm was adjusted by dividing by the number of other ethnic groups within a single study to avoid over‐counting as recommended in the Cochrane Handbook [17]. Detailed definitions of exposures, outcomes and reference groups are provided in Table 1.

**TABLE 1:** Definition of exposures and reference groups in the systematic review.

Factors/exposures	Definitions	Reference group
*Demographic factors*		
Education	Education was classified as further (college/university) and school‐level (from primary to high school). Due to variability in how key variables were reported across studies, only those with raw data were included in the meta‐analysis. For instance, education was categorised inconsistently—by level (e.g., ‘< high school,’ ‘college graduate’) or years of schooling (e.g., ‘< 9 years,’ ‘≥ 9 years’), and sometimes as ‘no formal,’ ‘primary,’ ‘secondary,’ or ‘tertiary’ education—making it difficult to harmonise data for pooled analysis	School education level
Ethnicity	White, Black, Asian, Other ethnic groups In multi‐ethnic studies, we adjusted the White reference group by dividing the number of White women by the number of other ethnic groups	White
Income	Income was classified as the highest and lowest level. Due to variability in reporting, only studies with raw data or reported in the same categories were included in the meta‐analysis	Lowest income level
Marital status	Marital status was classified as married and not married. Due to variability in reporting, only studies with raw data or reported in the same categories were included in the meta‐analysis	Married
Employment	Employment was classified as employed and not employed. Due to variability in reporting, only studies with raw data or reported in the same categories were included in the meta‐analysis	Employed level
Parity	Parity was classified as nulliparous, parous, parity one or two, and parity above two. Due to variability in reporting, only studies with raw data or reported in the same categories were included in the meta‐analysis	Nulliparous
*Behavioural factors*		
Alcohol intake	Alcohol was classified as any use and no use. Due to variability in reporting, studies with raw data or reported in the same categories were included in the meta‐analysis	No use
Physical activity	Physical activity was classified as yes (regular/high/most intensive) and no (irregular/light/no). Due to variability in reporting, studies with raw data or reported in the same categories were included in the meta‐analysis	No physical activity
Smoking	Smoking was classified as never, current, not current, or former. Due to variability in reporting, studies with raw data or reported in the same categories were included in the meta‐analysis	Never/not current
*Comorbidities*		
*BMI*	Body mass index (BMI) was a categorical variable where data allowed, including underweight (BMI ≤ 18.5), normal weight (BMI 18.5–24.9), overweight (BMI 25–29.9), obese (BMI 30–34.9)	Normal weight (BMI 18.5–24.9)
Anxiety	Anxiety was classified as low, moderate and high levels	Low level
Depression	Depression was classified as low, moderate and high levels	Low level

### 2.7 Statistical Analysis

The associations between demographic, behavioural and health characteristics and vasomotor symptoms were evaluated using ORs and corresponding 95% confidence intervals (95% CI). Inconsistency between studies was reported using the *I* ^2^ statistic. Meta‐analysis was undertaken separately for each exposure using a Hartung‐Knapp‐Sidik‐Jonkman (HKSJ) random effects model [18]. Where available, raw data were used to calculate odds ratios. If unavailable, reported unadjusted or adjusted odds ratios were extracted. When both were provided, raw data was prioritised. Results were presented in individual forest plots and summary forest plots grouped by type of exposure (demographic, behavioural and comorbidities). Statistical analyses were conducted using Stata/SE 18 (StataCorp LLC, College Station, TX) [19]. We conducted a sensitivity analysis restricted to studies that reported unadjusted odds ratios calculated directly from raw event counts.

## 3 Results

**FIGURE 1:**
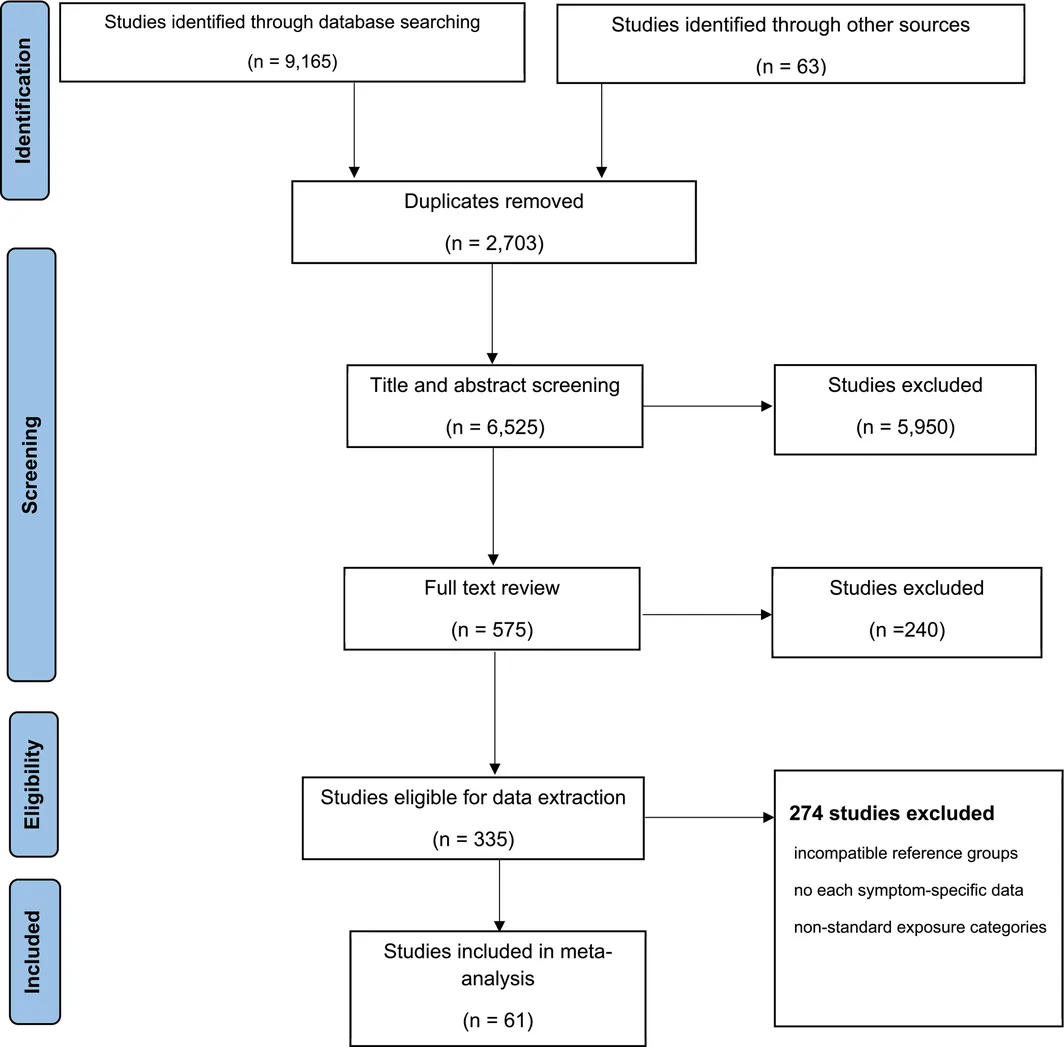
PRISMA flow diagram for study selection.

Searches identified 9228 articles from Medline (*n* = 4096), EMBASE (*n* = 2507), CINAHL (*n* = 1892) and Cochrane (*n* = 670). A further 63 of these articles were identified from reference lists of included articles. 2703 duplicates were removed, and 5950 studies were excluded following title and abstract screening leaving 575 studies for full‐text review. A further 240 studies were excluded for the following reasons: no eligible exposures (*n* = 122); wrong outcomes (*n* = 66), where menopausal symptoms were mentioned but not reported as one of the three prespecified symptom outcomes for meta‐analysis; wrong study design (*n* = 20); no usable numerical data (*n* = 12); wrong patient population (*n* = 14); and wrong indication (*n* = 6), leaving 335 studies for extraction. Data from 274 of 335 studies could not be extracted for meta‐analysis due to incompatible reference groups (*n* = 24), lack of symptom‐specific statistics (*n* = 120), or exposure/outcomes categories (*n* = 130) that did not align with our predefined classifications. 61 studies (279 813 participants) were included in the meta‐analysis of one or more factors for VMS, vaginal dryness and joint symptoms. The detailed study selection procedure is summarised in the PRISMA flowchart (Figure 1).

Characteristics of the 61 studies included in meta‐analyses are shown in Table 2. Studies were published between 1998 and 2023. 27 studies were from the USA, 4 from India and Australia, 2 from each of Brazil, Korea, China, Turkey and Iran, and one each from 16 other countries. 45 studies were from high‐income countries, 9 from upper‐middle‐income countries, 7 from middle‐income countries, using the World Bank classification [79]. 45 of the included studies were of cross‐sectional design, 13 were cohort studies, 3 were randomised trials.

**TABLE 2:** Characteristics of papers included in meta‐analysis.

Author, Year	Year	Country	Study design	Data collection period	Age of population	Sample size	Setting
Gartoulla 2015 [20]	2015	Australia	Cross‐sectional study	10/2013 to 3/2014	40–65	2020	General population
Herber‐Gast 2014 [21]	2014	Australia	Cross‐sectional study	1996, 1998, 2004 and 2010	45–50	4895	General population
Islam 2016 [22]	2016	Bangladesh	Cross‐sectional study	9/2013 to 3/2014	30–59	1590	General population
daSilva 2013 [23]	2013	Brazil	Cross‐sectional study	7/2011 to 1/2012	35–65	1415	Secondary care outpatients
Sclowitz 2005 [24]	2005	Brazil	Cross‐sectional study	2/2002–5/2002	40–69	879	Community
Huang 2020 [25]	2020	China	Cross‐sectional study	11/2018 to 11/2019	40–60	1501	Outpatient clinics
Armeni 2023 [26]	2023	Greece	Cross‐sectional study	9/2020 to 11/2021	43–75	281	Menopause clinic
Karmakar 2017 [27]	2017	India	Cross‐sectional study	not specified	40–60	100	General population
S 2020 [28]	2020	India	Cross‐sectional study	2/2015 to 5/2015	40–60	400	General population
Thakur 2019 [29]	2019	India	Cross‐sectional study	Not mentioned	35–55	351	General population
Fooladi 2018 [30]	2018	Iran	Cross‐sectional study	Not mentioned	40–64	1520	Community
Lerner‐Geva 2010 [31]	2010	Israel	Cross‐sectional study	6/2004 to 3/2006	45–64	814	General population
DiDonato 2005 [32]	2005	Italy	Cross sectional study	1997 to 2001	21–77	66 501	Outpatient clinic
Tomida 2021 [33]	2021	Japan	Cross‐sectional analyses	7/2010 to 7/2012	40–91	1152	General population
Kwon 2022 [34]	2022	Korea	Cross‐sectional analysis of a prospective cohort study	2014–2018	42–52	4164	Primary care
Hasan 2016 [35]	2016	Malaysia	Cross‐sectional study	3/2012 to 1/2013	> 35	640	Mix: secondary and primary care outpatients and community
Tsiligianni 2014 [36]	2014	Mediterranean Islands	Cross‐sectional study	Not mentioned	> = 65	851	General population
Olaolorun 2009 [37]	2009	Nigeria	Cross‐sectional study	Not mentioned	40–60	1189	Community
Li 2003 [38]	2003	Sweden	Cross‐sectional study	1996–2000	50–64	6917	Community (mailed questionnaire)
Mishra 2012 [39]	2012	UK	Cohort study	3/1/1946	47 to 54	695	General population
Crandall 2006 [40]	2006	USA	Cross‐sectional study	1995–1997	40–55	1467	Community‐based sampling
Ensrud 2009 [41]	2009	USA	Cross‐sectional study	2/2006 to 10/2006	40–60	217	Women recruited into an RCT
Ford 2005 [42]	2005	USA	Cohort study	A 10‐year period beginning in 1992	24–44	3192	General population
Freeman 2001_2 [43]	2001	USA	Cohort study	Not specified	35–48	375	General population
Freeman 2014 [44]	2014	USA	Cohort study	A 16‐year follow‐up period (1996–2012)	35–48 at entry of the cohort	255	General population
Freeman 2016 [45]	2016	USA	Cohort study	1996 to 2012	35–47	233	General population
Gallicchio 2015 [46]	2015	USA	Cohort study	2006 to 4/2014	45–54	732	Outpatient clinic
Gibson 2014 [47]	2014	USA	Cohort study	1996–1997	42–52	52	Secondary/research
Gold 2000 [48]	2000	USA	Cross‐sectional study	1995–1997	40–55	12 425	Community
Gold 2006 [7]	2006	USA	Prospective cohort	1996–2002	42–52	2784	Community
Huang 2008 [49]	2008	USA	Cross‐sectional study	Not specified	< 80 years	3167	General population
Im 2019 1 [50]	2019	USA	Cross‐sectional study		40–60	164	General population
Im 2019 2 [51]	2019	USA	Cross‐sectional study	2012 and 2010	40–60	1054	General population
Im 2010 [52]	2010	USA	Cross‐sectional study	Not specific	40–60	512	General population
Kroenke 2012 [53]	2012	USA	Trial study	1993 to 1998	50–79	17 473	‘The Women's Health Initiative (WHI) Dietary Modification (DM) trial’
Matthews 2020 [54]	2020	USA	A multisite observational study	1995–1997	42–52	1407	General population
Mukarram 2021 [55]	2021	USA	Cross‐sectional study	6/2017 to 2/2018	40–65	104	General population
Reed 2013 [56]	2013	USA	Cross‐sectional study	Not mentioned	45–58	5634	General population
Sievert 2006 [57]	2006	USA	Cross‐sectional study	2001–2002	45–55	293	Secondary/research institution
Staropoli 1998 [58]	1998	USA	Cross‐sectional study	5/1994–9/1994	45–65	233	Outpatient clinic
Thurston 2008 [59]	2008	USA	Cohort study	2003 to 2005	42–52	1042	General population
Whitcomb 2007 [60]	2007	USA	Cross‐sectional study	2001	40–60	512	Community (mail)
Ahmadieh 2021 [61]	2021	Lebanon	Cross‐sectional study	7/2016 to 4/2017	45–75	623	General population
Aydin 2014 [62]	2014	Turkey	Cross‐sectional study	2005 to 2012	> = 40	1071	Outpatient clinic
Bozkurt 2007 [63]	2007	Turkey	Cross‐sectional study	2/1/2005	37–83	2365	General population
Nunez‐Pizarro 2017 [64]	2017	Latin America	Cross‐sectional study	Not mentioned	40–59	3503	General population
Huang 2010 [65]	2010	USA	Cohort study	1998–2002	55–75	1017	General population
Nisar 2015 [66]	2015	Pakistan	Cross‐sectional study	8/1/2007	40–70	3062	General population
Worsley 2017_2 [67]	2017	Australia	Cross‐sectional study	10/2013 to 3/2014	40–65	2020	General population
Freeman 2005 [68]	2005	USA	Cohort study	Not mentioned	35–47	436	General population
Aldrighi 2004 [69]	2004	not stated	Trial study	Not specific	45–75	487	General population
Reed 2014 [70]	2014	USA	Cross‐sectional study	3/2010–2/2011	45–56	1513	General population
Gupta 2006 [71]	2006	India	Cross‐sectional study	Not mentioned	45–55	153	General population
Pastore 2004 [72]	2004	USA	Cross‐sectional study	28/02/1997	50–79	98 705	Outpatient clinic
Delavar 2011 [73]	2011	Iran	Cross‐sectional study	Not specific	45–63	1397	General population
Whiteman 2003 [74]	2003	USA	Cross‐sectional study	2001	40–60	1087	General population
Gartoulla 2018 [20]	2018	Australia	Cross‐sectional study	10/2013 to 3/2014	40–65	2020	General population
Koo 2017 [75]	2017	Korea	Cross‐sectional study	2012–2013	44–56	533	General population
Ryu 2020 [76]	2020	Korea	Cross‐sectional study	2010–2012	45–65	2481	General population
Tang 2022 [77]	2022	China	Cohort study	Started 7/2005 and followed up annually	35 to 64	430	General population
Faubion 2023 [78]	2023	USA	Cohort study	15/5/2015–31/1/2022	45–60	5708	General population

### 3.1 Risk of Bias Within Studies

All papers included in the meta‐analysis had mean quality assessment scores between 20 and 40, indicating moderate risk of bias overall; however, 55 papers scored ≤ 30, suggesting a lower level within that range (Figure S1). Figure S2 shows the distribution of mean scores across the six main items in our adapted tool. Most studies were rated as moderate risk of bias across all domains. Specifically: for study population, 23 papers were low, 35 moderate and 3 high risk; for exposure measurement, 4 low, 55 moderate, 2 high; for outcome measurement, 6 low and 55 moderate; for comparability, 32 low, 25 moderate, 4 high; for confounding, 6 low, 54 moderate, 1 high; and for statistical analysis/reporting, 37 low, 24 moderate.

### 3.2 Menopause Symptoms

Of the 61 included studies, 54 reported the presence, frequency and/or severity of VMS. Eight studies reported on the presence and/or severity of vaginal dryness, while four studies reported joint symptoms.

Instruments used to identify women with menopause symptoms and their severity included the Menopause Rating Scale, the Menopause‐Specific Quality of Life questionnaire and the Kupperman Menopause Index, the Menopause Health Questionnaire, Greene Climacteric Scale and Menopause Symptom Scale and Female Sexual Function Index. Other studies used study‐specific questionnaires. In instruments used to identify women experiencing menopausal symptoms, the recall periods varied from 2 weeks to ‘ever’ (i.e., lifetime recall). Symptom severity was rated on a 0–3 scale (none to severe), with specific weighting applied. For analysis, severity was dichotomised into moderate/severe vs. none/mild to improve comparability across studies and account for variability in reporting.

Menopausal status criteria varied across the studies. Nine studies included mixed menopausal groups, while one only included postmenopausal women. Five studies examined women transitioning from premenopause to perimenopause, and another three from perimenopause to postmenopause. Thirty‐one papers specifically addressed transitional phases, spanning from early premenopause to late perimenopause and into postmenopause. However, menopausal status was unclear in 15 papers. Based on the Stages of Reproductive Aging Workshop [80] staging system, premenopause typically refers to women under 40 with regular menstrual cycles; perimenopause generally occurs between ages 40 and 55 and is marked by menstrual irregularities and hormonal fluctuations; and postmenopause begins after 12 consecutive months of amenorrhea, usually from around age 50 onward.

Data for exposure factors examined were as follows: demographic (30 studies), behavioural [81] and comorbidities [9]. Table 3 shows pooled ORs, 95% CI, *p* values, *I* ^2^ and number of papers for associations with VMS, vaginal dryness and joint symptoms from meta‐analyses.

**TABLE 3:** Pooled odds ratios for all factors and menopause symptoms.

Factors	OR	95% CI		*p*	*I* ^2^	Number of included studies	Supporting Information figures
*Demographic factors*							
Education (University or College vs. School Education)						23	
VMS							
Presence	1.31	1.09	1.56	0.006	88.8	16	Figure S3a
Severity	0.94	0.65	1.35	0.7	97.4	8	Figure S3b
Vaginal dryness							
Presence	1.12	0.82	1.52	0.364	72.8	5	Figure S3c
Ethnicity							
VMS							
Black (vs. White)							
Presence	1.65	1.41	1.94	< 0.001	12.1	8	Figure S4a
Severity	1.91	1.10	3.29	0.028	87.0	7	Figure S4b
Asian (vs. White)							
Presence	0.4	0.22	0.720	0.011	75.4	6	Figure S4c
Severity	0.55	0.53	0.560	< 0.001	0.0	4	Figure S4d
Other (vs. White)							
Presence	0.83	0.68	1.01	0.065	42.3	12	Figure S4e
Severity	1.33	0.80	2.22	0.238	63.2	12	Figure S4f
Vaginal dryness							
Black (vs. White)							
Presence	1.27	1.10	1.47	0.019	19.9	3	Figure S4g
Non‐White (vs. White)							
Presence	1.14	0.91	1.44	0.222	90.9	12	Figure S4h
Joint Symptoms							Figure S4i
Black (vs. White)							
Presence	0.96	0.23	4.07	0.915	74.2	3	
Asian (vs. White)							
Presence	0.81	0.58	1.14	0.199	50.3	11	
Other (vs. White)							
Presence	1.2	0.48	3.03	0.628	78.3	6	
Income (High vs. Low)							
VMS							
Presence	1.41	1.01	1.97	0.046	72.9	7	Figure S5
Marital status							
VMS							
Presence	0.89	0.77	1.03	0.103	71.4	6	Figure S6a
Vaginal dryness							
Presence	1.39	0.53	3.64	0.276	92.9	3	Figure S6b
Employment							
VMS							
Presence	1.001	0.84	1.19	0.986	38.0	9	Figure S7
*Behaviour factors*							
Alcohol intake							
VMS							
Presence	1.06	0.67	1.66	0.731	71.6	4	Figure S8a
Severity	1.2	0.80	1.80	0.278	59.5	5	Figure S8b
Physical activity							
VMS							
Presence	1.016	0.59	1.76	0.939	70.7	5	Figure S9a
Vaginal dryness							
Presence	0.86	0.24	3.07	0.665	94.4	3	Figure S9b
Smoking							
VMS							
Current versus not							
Presence	1.63	1.30	2.04	0.01	62.5	8	Figure S10a
Severity	1.56	1.07	2.27	0.027	82.4	8	Figure S10b
Vaginal dryness							
Smoking versus no							
Presence	1.41	0.73	2.73	0.217	90.1	5	Figure S10c
BMI categories							
VMS							
Overweight BMI							
Presence	1.198	1.00	1.44	0.052	61.5	13	Figure S11a
Severity	1.192	0.97	1.46	0.087	63	10	Figure S11b
Obese BMI							
Presence	1.349	1.02	1.79	0.038	79.3	14	Figure S11a
Severity	1.424	1.11	1.83	0.01	82.9	12	Figure S11b
Vaginal dryness							
Obese BMI							
Presence	1.13	0.67	1.92	0.417	71.1	3	Figure S11c
Parity (> 2 children vs. 0–2 children)							
Vaginal dryness	0.88	0.38	2.07	0.598	84.6	3	Figure S12
Presence							
*Comorbidities*							
Depression							
VMS							Figure S13
High versus lowest level							
Severity	2.36	1.51	3.70	0.004	91.2	6	
Diabetes							
Vaginal dryness							
Presence	1.27	0.61	2.63	0.296	13.8	3	Figure S14

### 3.3 Demographic Factors

**FIGURE 2:**
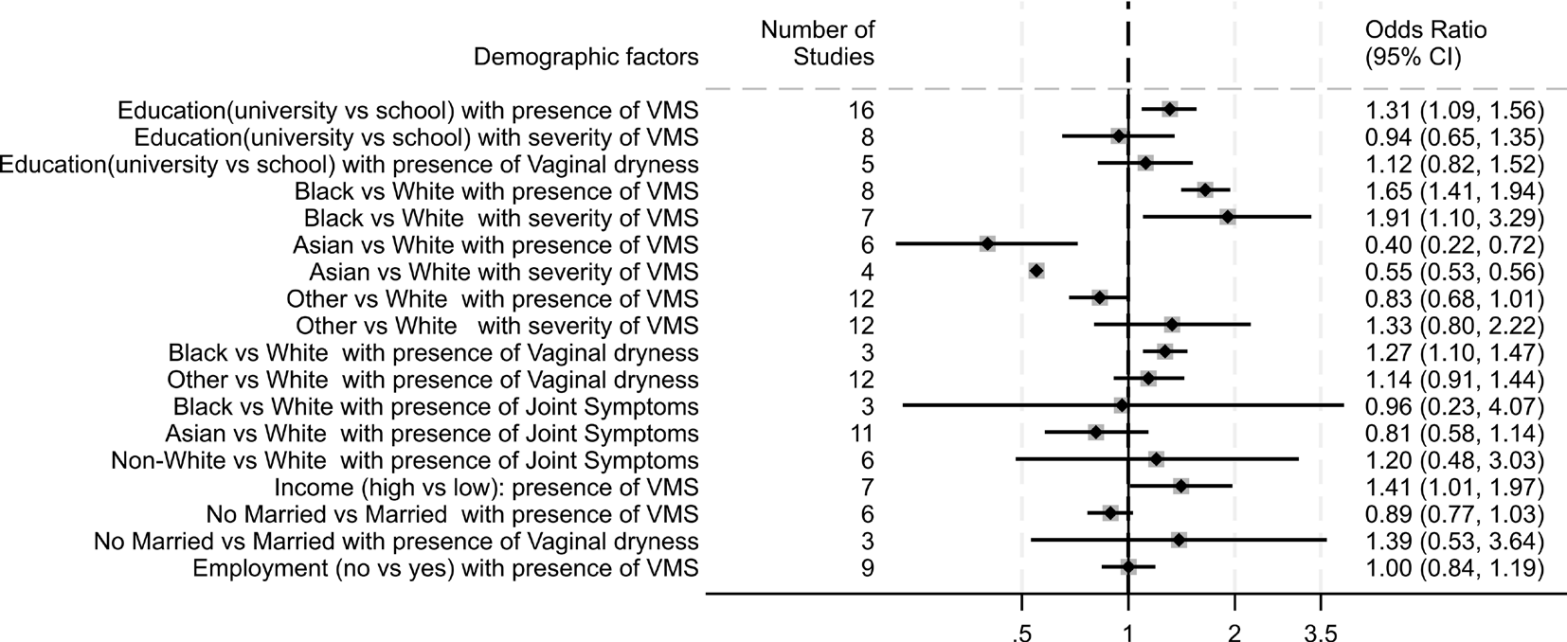
Association of demographic factors with VMS, vaginal dryness and joint symptoms.

Results of the meta‐analyses of demographic factors and menopause symptoms are summarised in Figure 2. Women with a university or college education had a higher likelihood of experiencing VMS compared to those with only school‐level education (OR 1.31, 95% CI 1.09–1.56). However, there was no significant association between education level and VMS severity (OR 0.94, 95% CI 0.65–1.35). There was also no significant association between education level and the presence of vaginal dryness (OR 1.12, 95% CI 0.82–1.52). High‐income women had more frequent VMS presence than low‐income women (OR 1.41, 95% CI 1.01–1.97).

Compared to White women, Black women had higher VMS presence (OR 1.59, 95% CI 1.34–1.88) and VMS severity (OR 1.91, 95% CI 1.10–3.29), while Asian women had lower VMS presence (OR 0.40, 95% CI 0.31–0.51) and VMS severity (OR 0.48, 95% CI 0.33–0.70). No significant differences were observed for other ethnic groups vs. White with VMS. Black women also had a higher presence of vaginal dryness (OR 1.27, 95% CI 1.1–1.47). There was no significant association between ethnicity and joint symptoms.

Marital status and employment showed no significant associations with VMS (ORs 0.89, 95% CI 0.77–1.03 and 1.00, 95% CI 0.84–1.19, respectively). There were also no significant associations between marital status and presence of vaginal dryness (OR 1.39, 95% CI 0.53–3.64). Forest plots illustrating the findings for each factor are presented in Figures S3–S7.

### 3.4 Behavioural Factors

**FIGURE 3:**
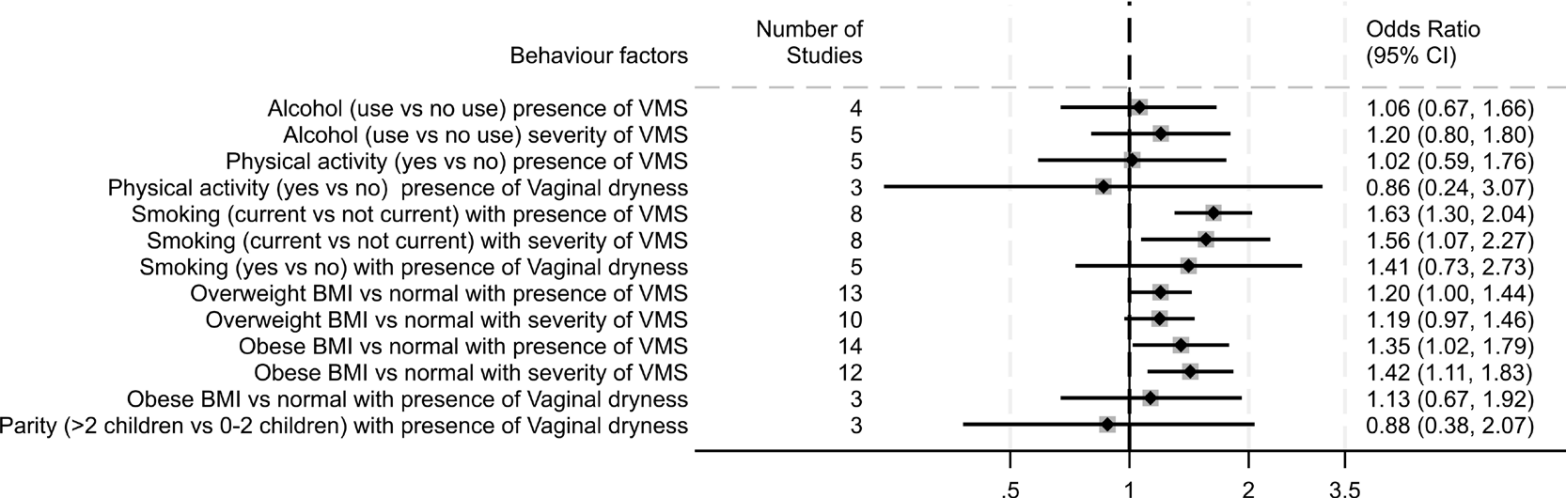
Association of behavioural factors with VMS, and vaginal dryness.

The included studies investigated associations between menopause symptoms and alcohol, physical activity, smoking, BMI and parity. The number of studies for each factor and associations with symptoms are shown in Figure 3. Alcohol consumption was not associated with VMS presence (OR 1.06, 95% CI 0.67–1.66) or severity (OR 1.20, 0.80–1.80). Current and ever smokers had higher VMS presence and severity, compared to non‐smokers (OR 1.63, 1.30–2.04; OR 1.56, 1.07–2.27 respectively). There were no significant associations between smoking and vaginal dryness symptoms (OR 1.41, 0.73 2.73) or physical activity and VMS presence (OR 1.02, 0.59–1.76) or presence of vaginal dryness (OR 0.86, 0.24–3.07).

For BMI, both overweight and obesity were associated with a higher presence of VMS, with odds ratios of 1.20 (1.00–1.44) and 1.35 (1.02–1.78), respectively. Obesity was significantly associated with greater VMS severity (OR 1.42, 1.11–1.83), whereas the association between overweight and severity was not statistically significant (OR 1.19, 0.97–1.47). However, obesity was not associated with a higher presence of vaginal dryness (OR 1.13, 0.67–1.92). Having more than two children was not associated with the presence of vaginal dryness. Forest plots for each factor are presented in Figures S8–S12.

### 3.5 Comorbidities

Only depression and diabetes reported sufficient studies for meta‐analysis. Depression was significantly associated with higher VMS severity (OR 2.36, 95% CI 1.51–3.70). There were no associations between diabetes and the presence of vaginal dryness (OR 1.27, 0.61–2.63). Forest plots for each factor are presented in Figures S13 and S14.

### 3.6 Subgroup and Sensitivity Analyses

Sensitivity analyses using only raw data examining demographic (education, ethnicity), behavioural (BMI) and contextual factors (region, country income level) demonstrated patterns broadly consistent with the primary findings (Table S2). Subgroup analyses for education and the presence of vasomotor symptoms (VMS) showed that effect estimates showed some variation by region, but these differences did not follow a consistent or interpretable pattern, and most regional strata included too few studies to support reliable inference. Similarly, subgroup analyses by country income group showed no clear differences. For ethnicity, pooled estimates for ‘Other’ ethnicity and Non‐White groups did not materially differ from the main results for VMS or vaginal dryness. For Asian ethnicity and joint symptoms, the main analysis used raw data only so no sensitivity analyses were conducted. Sensitivity analyses for BMI found no significant associations between overweight and VMS severity or presence. Obesity was significantly associated with VMS severity (OR 1.429, 1.078–1.895). Across all domains, sensitivity estimates were largely comparable in direction and magnitude to the primary analyses, with wide intervals and heterogeneity reflecting limited study numbers and reliance on unadjusted data.

### 3.7 Narrative Synthesis Results From Studies Excluded From Meta‐Analysis

Across the excluded studies, most reported consistent positive associations between a range of sociodemographic, lifestyle, health and psychosocial factors and the severity of menopausal symptoms. Older age [82, 83], lower education [18, 81], obesity [19, 84, 85, 86], chronic disease [87, 88] and marginalised socioeconomic status [89, 90] were frequently linked with greater overall symptom burden, particularly somatic and psychological symptoms. Several studies also found stronger symptoms among rural women [89, 91], those with limited social support [81], or those reporting low quality of life [92, 93]. Conversely, higher physical activity [94, 95], healthier dietary patterns [96, 97] and positive health perception were generally associated with reduced symptom severity. Some studies identified specific predictors of higher symptom scores (e.g., low vitamin D, smoking, insomnia, or high parity) [19, 98, 99, 100], while others highlighted notable ethnic differences in symptom profiles [101, 102, 103, 104]. Overall, these findings suggest a moderate to strong direction of effect, indicating that social disadvantage, poorer health and adverse lifestyle factors are consistently related to more severe menopausal symptoms.

## 4 Discussion

### 4.1 Summary of Key Findings

The systematic review and meta‐analyses have combined data from published literature across the world to reveal that several demographic and behavioural factors are associated with experiencing menopause symptoms and severity of symptoms. Women with university or college education were 31% more likely to experience vasomotor symptoms, and those with higher income levels were 41% more likely than those with lower incomes to report presence of VMS. Black women were 65% more likely to experience VMS and 91% more likely to have severe VMS compared with White women, and Asian women were 60% less likely to experience VMS and 52% less likely to have severe VMS compared with White women.

Smoking, obesity and depression were associated with increased odds of VMS presence and severity: smokers were approximately 60% more likely to experience VMS presence and severe VMS; women with obesity were about 40% more likely for both; and those with depression were 2.4 times more likely to experience severe VMS.

Black women were 27% more likely to experience vaginal dryness compared with White women. We found no associations between any other factors and the presence or severity of vaginal dryness. We found no associations between ethnicity and the presence of joint symptoms.

### 4.2 Discussion in Context of Existing Literature

These findings are consistent with Blackson et al. [105], who, in a systematic review of observational studies, reported that racial disparities and chronic psychosocial stress contribute to earlier onset and more severe VMS, with African American women disproportionately affected. These findings are further supported by evidence from the SWAN study [106], a large, multi‐ethnic prospective longitudinal cohort study, which revealed that racial discrimination is independently associated with increased reporting of VMS, even after controlling for other factors.

Our finding that higher‐income women were more likely to report vasomotor symptoms (VMS) may reflect greater health literacy or a stronger sense of empowerment to seek support [107]. This interpretation likely reflects the reliance on self‐reported questionnaires rather than clinical records, which may exacerbate socioeconomic differences in symptom reporting.

Our finding of strong associations between smoking and obesity with both VMS presence and severity echoes findings from a previous pooled analysis report of 20 observational studies from Australia, China, Japan, Norway, Sweden, UK and USA, which demonstrated a dose–response relationship between these factors and VMS risk, particularly among obese smokers [108].

Our finding of an association between depression and vasomotor symptom severity may be influenced by unmeasured treatment factors (e.g., selective serotonin reuptake inhibitors), which can affect VMS severity [109]. This limitation is particularly relevant given the disparities in treatment access among women with obesity and those of Black ethnicity, who often experience more severe symptoms but are less likely to receive hormone replacement therapy (HRT) [110]. Nonetheless, our findings align with a recent international systematic review (2000–2021) (2023) [111], which highlighted the role of hormonal changes, sleep disturbances and VMS in increasing depression risk during menopause, with Black women again disproportionately affected and undertreated. Similarly, the strong association between depression and increased VMS severity [112], suggests that mental health screening and support should be integrated into menopause care pathways, particularly for women with overlapping vulnerabilities.

Notably, women experiencing the most severe VMS are not always those receiving hormone replacement therapy (HRT), especially among women with obesity and those of Black ethnicity [110]. This highlights potential disparities in access, uptake, or clinical decision‐making around HRT. Ethnic differences in symptom burden and treatment are also evident: Asian women consistently report fewer VMS compared to White and Black women, and while they receive HRT less frequently than White women, their usage rates remain higher than those observed in Black women [110]. Our review has found that more affluent and educated women report more severe symptoms and this group is more likely to receive HRT [16, 110]. But levels of smoking and obesity are higher in areas of deprivation and our work has determined that obesity is associated with more severe VMS [113, 114]. These disparities highlight the need for equitable, culturally sensitive and individualised menopause care.

Differences in HRT use, healthcare access and cultural norms likely contribute further to the heterogeneity observed across studies. Women with obesity and Black women have lower rates of HRT uptake, meaning that populations already affected by socioeconomic disadvantage and depression may have fewer opportunities to benefit from effective symptom management. This reduced access can heighten the severity of their vasomotor symptoms, thereby reinforcing the associations we observed. In this context, inequitable access to treatment increases symptom burden rather than alleviating it. Cultural variation in symptom perception and help‐seeking may also influence reporting patterns [115]. These structural and cultural factors should therefore be considered when interpreting between‐study variability in symptom prevalence and severity.

Behavioural factors such as smoking and obesity were consistently correlated with increased vasomotor symptom severity, reinforcing the importance of lifestyle interventions in symptom management [16]. Although behavioural interventions such as smoking cessation and weight management are effective in reducing vasomotor symptom severity, access to these services remains inequitable [116, 117, 118]. Barriers including cost, limited geographic availability and poor integration into routine menopause care contribute to under‐utilisation, particularly among socioeconomically disadvantaged groups [119].

### 4.3 Strengths and Limitations

To our knowledge, this is the first systematic review and meta‐analysis of factors associated with menopause symptoms. We included studies from any country and in any language to maximise the generalisability of our findings. We combined data, using a robust statistical approach, employing the HKSJ random effects model to minimise the chances of having spuriously significant findings [108]. Duplicate screening and data extraction checking were used to minimise the risk of errors. Combining elements of the Newcastle‐Ottawa Scale and the QUIPS tool enabled a tailored quality assessment that captured both methodological rigour and risk of bias in prognostic studies, enhancing the relevance of the evaluation. We have followed methods recommended in the Cochrane Handbook throughout to ensure that analyses are reproducible and robust [17].

This review has several limitations. A quarter of included studies were published over 20 years ago, and most originated from the USA, Australia, India and Brazil, limiting generalisability, particularly to low‐ and middle‐income countries. Comparisons of additional menopausal symptoms (e.g., sexual, sleep‐related, or psychological symptoms) across ethnic groups were not undertaken, and based on the available data it is not possible to determine whether such analyses could have been meaningfully conducted, as most studies consistently reported only vasomotor symptoms, vaginal dryness, or joint symptoms in Black and White women, with few reporting the latter two outcomes. Age‐stratified analyses were also unfeasible due to substantial inter‐study differences, despite evidence that symptom patterns vary with age. The included studies did not apply standardised thresholds for severity of symptoms and most studies did not specify the recall period used to assess symptom presence or severity. This may affect the comparability and interpretation of symptom prevalence across studies which is a limitation of our work. Our primary analysis included both unadjusted and adjusted odds ratios for associations between factors and symptoms. We prioritised unadjusted odds ratios over adjusted values as these were most commonly reported and covariates that were adjusted for differed between each study. To account for this, we conducted sensitivity analyses to restrict analysis to unadjusted odds ratios only which determined that this did not change the overall findings for any of the outcomes or exposures tested. The number of studies in each comparison was relatively low, with only a few comparisons having more than 10 studies. This limited our ability to explore heterogeneity through subgroup analysis and meta‐regression [17].

### 4.4 Implications for Practice, Policy and Future Research

By combining data from 61 studies, this systematic review and meta‐analysis have determined that Black women are more likely to experience menopausal vasomotor symptoms than White women and their symptoms are more severe. We know that Black women in the UK are significantly less likely to use hormone replacement therapy than their White counterparts [110], and a recent systematic review and meta‐analysis of international studies has confirmed that this pattern is similar in other countries [16]. These findings have important implications for clinical practice, public health policy and future research on menopause symptom management to determine whether there are barriers to accessing healthcare or treatments in some communities or whether more information is needed on options for women during the menopause transition. A recent qualitative study also highlighted women's dissatisfaction with current choices and underscored the need for better communication about HRT benefits and risks in UK primary care [120]. Together with our systematic review of factors associated with HRT use [16] and cohort study of HRT prescribing patterns in UK primary care [110], these findings suggest potential gaps in menopause care for some women. However, the drivers of these disparities are likely multifactorial and context‐dependent. For instance, in the U.S., financial barriers may limit healthcare access for Black women, affecting symptom reporting and treatment uptake.

The findings of this paper may encourage clinicians to consider proactively discussing menopause or menopause treatments with women of Black ethnicity or low education or income, who are likely to experience a higher burden of menopause symptoms while remaining mindful of individual circumstances and structural barriers to care.

Behavioural factors such as smoking and obesity were linked to vasomotor symptom severity, reinforcing the value of lifestyle interventions in menopause care. Chung et al. [108] demonstrated that *smoking and elevated body weight are associated with increased vasomotor symptom burden; however, prospective and interventional studies are needed to determine whether modifying these risk factors can reduce* symptoms, especially among high‐risk groups.

The narrative synthesis broadly supports the meta‐analysis findings, reinforcing the role of socioeconomic, lifestyle and health‐related factors in shaping menopausal symptom burden. Several non‐meta‐analysed studies similarly indicated that lower education, poorer socioeconomic conditions, obesity, chronic illness and limited social support were associated with more severe symptoms, aligning with pooled evidence showing higher VMS presence among high‐income and university‐educated women, and greater VMS severity among obese women and smokers. The narrative evidence also echoed ethnic differences observed in the meta‐analysis, with some studies reporting heightened symptoms among marginalised or rural groups. Consistent with the pooled associations between depression and VMS severity, several descriptive studies highlighted strong links between psychological distress and symptom burden. Overall, findings from non‐meta‐analysed studies strengthened the direction and plausibility of the meta‐analytic results, despite variability in measurement and insufficient data for quantitative synthesis.

Expanding access through primary care and community programs could improve uptake and symptom management, reducing disparities. Future research should evaluate integrated care models that enhance accessibility and engagement, ensuring lifestyle interventions are feasible for all women with VMS.

Future research should examine longitudinal symptom patterns and test interventions addressing these influences, while healthcare systems should prioritise inclusive education, prevention and support services to improve quality of life during the menopausal transition.

These findings underscore the complex interplay of sociodemographic, behavioural and psychosocial factors in shaping menopause experiences and highlight the need for more inclusive, holistic, context and culturally sensitive approaches to care.

## Author Contributions

Jennifer Hirst, J.H.‐C., C.C., and S.H. contributed to the initiative of the research question, study design, interpretation, and obtaining funding for the study. Janice Hoang, D.A., E.T., G.G., W.M.M., and Jennifer Hirst participated in study screening and selection and data extraction. Janice Hoang, K.H., D.A., and W.M.M. performed the meta‐analysis. K.H., D.A. and Janice Hoang conducted the quality assessment of included papers. D.A. and Janice Hoang conducted the narrative synthesis for non‑meta‑analysable studies. Janice Hoang wrote the first draft of the manuscript. L.T. contributed to the Patient and Public Involvement and Engagement component of this study. All authors reviewed and commented on manuscript drafts and approved the submitted version. [Correction added on 13 May 2026, after first online publication: The Author Contribution section has been updated in this version.].

## Funding

This project is funded by the National Institute for Health and Care Research (NIHR) under its Research for Patient Benefit (RfPB) Programme (Grant Reference Number NIHR204901). The views expressed are those of the author(s) and not necessarily those of the NIHR or the Department of Health and Social Care.

## Disclosure

*Patient and public involvement*: A patient co‐applicant (LT) supported us to get the funding for this study and was involved throughout in study management. We recruited an ethnically and socially diverse group of five women who helped us make sense of the results and supported us with finding ways to communicate these back to the communities who needed it most.

## Conflicts of Interest

Dr. Sarah Hillman was sponsored by the pharmaceutical company Besins to attend the 2024 International Menopause Society Conference. No other authors declare competing interests.

## Supporting information


**Appendix S1:** Search strategy and history in Medline, EMBASE, CINAHL.
**Table S1:** A list of excluded studies and reasons.
**Table S2:** Sensitivity analysis findings.
**Table S3:** Summary of small study effect tests (Egger's regression).
**Table S4:** Overview of non‐pooled study findings and methodological incompatibilities.
**Figure S1:** The distribution of risk scores of biases in overall quality.
**Figure S2:** The distribution of mean scores across the six main items in our adapted tool.
**Figure S3a:** Association of education with presence of VMS.
**Figure S3b:** Association of education with severity of VMS.
**Figure S3c:** Association of education with presence of vaginal dryness.
**Figure S4a:** Association of ethnicity (Black vs. White) with presence of VMS.
**Figure S4b:** Association of ethnicity (Black vs. White) with severity of VMS.
**Figure S4c:** Association of ethnicity (Asian vs. White) with presence of VMS.
**Figure S4d:** Association of ethnicity (Asian vs. White) with severity of VMS.
**Figure S4e:** Association of ethnicity (Others vs. White) with presence of VMS.
**Figure S4f:** Association of ethnicity (Others vs. White) with severity of VMS.
**Figure S4g:** Association of ethnicity (Black vs. White) with presence of vaginal dryness.
**Figure S4h:** Association of ethnicity (Non‐White vs. White) with presence of vaginal dryness.
**Figure S4i:** Association of ethnicity (Black, Asian, Other vs. White) with presence of Joint Symptoms.
**Figure S5:** Association of income (high vs. low) with presence of VMS.
**Figure S6a:** Association of marital status (no married vs. married) with presence of VMS.
**Figure S6b:** Association of marital status (no married vs. married) with presence of vaginal dryness.
**Figure S7:** Association of employment (highest vs. lowest level) with presence of VMS.
**Figure S8a:** Association of alcohol (use vs. no use) with presence of VMS.
**Figure S8b:** Association of alcohol (use vs. no use) with severity of VMS.
**Figure S9a:** Association of physical activity with presence of VMS.
**Figure S9b:** Association of physical activity with presence of Vaginal dryness.
**Figure S10a:** Association of smoking (current vs. not current) with presence of VMS.
**Figure S10b:** Association of smoking (current vs. not current) with severity of VMS.
**Figure S10c:** Association of smoking (smoking vs. no smoking) with presence of Vaginal dryness.
**Figure S11a:** Association of overweight and obese BMI with presence of VMS.
**Figure S11b:** Association of overweight and obese BMI with severity of VMS.
**Figure S11c:** Association of obese BMI with presence of vaginal dryness.
**Figure S12:** Association of Parity (> 2 children vs. 0–2 children) with presence of vaginal dryness.
**Figure S13:** Association of depression (high vs. lowest level) with severity of VMS.
**Figure S14:** Association of diabetes with presence of vaginal dryness.
**Figure S15:** Funnel plot for Egger's test assessing small‑study effects for education (university/college vs school education) and presence of vasomotor symptoms (VMS).
**Figure S16:** Funnel plot for Egger's test assessing small‑study effects for ethnicity (other vs White) and presence of vasomotor symptoms (VMS).
**Figure S17:** Funnel plot for Egger's test assessing small‑study effects for ethnicity (other vs White) and severity of vasomotor symptoms (VMS).
**Figure S18:** Funnel plot for Egger's test assessing small‑study effects for ethnicity (non‑White vs White) and presence of vaginal dryness.
**Figure S19:** Funnel plot for Egger's test assessing small‑study effects for ethnicity (Asian vs White) and presence of joint symptoms.
**Figure S20:** Funnel plot for Egger's test assessing small‑study effects for overweight BMI and presence of vasomotor symptoms (VMS).
**Figure S21:** Funnel plot for Egger's test assessing small‑study effects for overweight BMI and severity of vasomotor symptoms (VMS).
**Figure S22:** Funnel plot for Egger's test assessing small‑study effects for obese BMI and presence of vasomotor symptoms (VMS).
**Figure S23:** Funnel plot for Egger's test assessing small‑study effects for obese BMI and severity of vasomotor symptoms (VMS).

**Appendix S2:** Outlining the responsibilities of each corresponding author.

## Data Availability

Data sharing not applicable to this article as no datasets were generated or analysed during the current study.
