# Supplementary material for: Factors Associated With Menopause Symptoms: A Systematic Review and Meta‐Analysis
Source: BJOG. 2026 May 6;133(11):1967–85. doi: 10.1111/1471-0528.70257 (PMC13571893; doi:10.1111/1471-0528.70257)
Supplement: Supplementary file 1 [file BJO-133-1967-s001.docx]

# **Supplementary Data List:**

- Appendix S1: Search strategy and history in Medline, EMBASE, CINAHL
- Table S1: A list of excluded studies and reasons
- Table S2: Sensitivity Analysis Findings
- Table S3: Summary of Small Study Effect Tests (Egger’s Regression)
- Table S4: Overview of Non‑Pooled Study Findings and Methodological Incompatibilities
- Figure S1: The distribution of Risk Scores of Biases in Overall Quality
- Figure S2: The distribution of mean scores across the six main items in our adapted tool.

Demographic factors

- Figure S3a: Association of Education with presence of VMS
- Figure S3b: Association of Education with severity of VMS
- Figure S3c: Association of Education with presence of Vaginal dryness
- Figure S4a: Association of Ethnicity (Black vs White) with presence of VMS
- Figure S4b: Association of Ethnicity (Black vs White) with severity of VMS
- Figure S4c: Association of Ethnicity (Asian vs White) with presence of VMS
- Figure S4d: Association of Ethnicity (Asian vs White) with severity of VMS
- Figure S4e: Association of Ethnicity (Others vs White) with presence of VMS
- Figure S4f: Association of Ethnicity (Others vs White) with severity of VMS
- Figure S4g: Association of Ethnicity (Black vs White) with presence of Vaginal dryness
- Figure S4h: Association of Ethnicity (Non-White vs White) with presence of Vaginal dryness
- Figure S4i: Association of Ethnicity (Black, Asian, Other vs White) with presence of Joint Symptoms
- Figure S5: Association of Income (high vs low) with presence of VMS
- Figure S6a: Association of Marital Status (No Married vs Married) with presence of VMS
- Figure S6b: Association of Marital Status (No Married vs Married) with presence of Vaginal dryness
- Figure S7: Association of Employment (highest vs lowest level) with presence of VMS

Behaviour factors

- Figure S8a: Association of Alcohol (use vs no use) with presence of VMS
- Figure S8b: Association of Alcohol (use vs no use) with severity of VMS
- Figure S9a: Association of Physical activity with presence of VMS
- Figure S9b: Association of Physical activity with presence of Vaginal dryness
- Figure S10a: Association of Smoking (current vs not current) with presence of VMS
- Figure S10b: Association of Smoking (current vs not current) with severity of VMS
- Figure S10c: Association of Smoking (smoking vs no smoking) with presence of Vaginal dryness
- Figure S11a: Association of Overweight and Obese BMI with presence of VMS
- Figure S11b: Association of Overweight and Obese BMI with severity of VMS
- Figure S11c: Association of Obese BMI with presence of Vaginal dryness
- Figure S12: Association of Parity (>2 children vs 0-2 children) with presence of Vaginal dryness

Comorbidities

- Figure S13: Association of Depression (high vs lowest level) with severity of VMS
- Figure S14: Association of Diabetes with presence of Vaginal dryness

Funnel Figures for Table S3 (Figure S15-23)

Appendix S1: Search strategy and history in Medline, EMBASE, CINAHL

**Search strategy and history in Medline, EMBASE, CINAHL**

*Medline Search:*

**#, Searches, Results, Type**

1, (postmenopaus* or menopaus* or perimenopaus*).ti,ab,kf., **106282**, Advanced

2, symptom*.mp., **1476249**, Advanced

3, (Symptom* or Hot flush or hot flash or hot flushes or vasomotor or Psychological or Locomotor or Urogenital or Somatic or Night sweats or Vaginal dryness or Loss of sex drive or Less sexual feelings or libido or incontinence or Urinary frequency or Bone thinning or osteoporosis or Low mood or Mood changes or Fatigue or Insomnia or Palpitations or Cognitive symptoms or Brain fog or Mental fog or Depress* or Anxi* or Lightheaded or Headache* or Irritab* or Anxiety or Sleeplessness or (sleep adj5 problem*) or (sleep adj5 difficult*) or Backache or Joint pains or Muscle pains or Pain or New facial hair or Dry skin or Dry vagina or vaginal atrophy).mp., **3693796**, Advanced

4, (Troublesome or problem* or sever* or bothersome or acute or disruptive or ((poor or low or decreased or adverse) adj5 quality of life) or Impact on daily routine).mp., **6004008**, Advanced

5, (factor* or demographic* or social class or socioeconomic or deprivation or ethnicity or characteristic* or risk or determinant or predict* or associat* or ((hazard or risk or odds) adj5 ratio)).mp., **13539777**, Advanced

6, 1 and 2 and 3 and 4 and 5, **4108**, Advanced

*EMBASE search:* 
1, Symptom.mp., **679113**, Advanced

2, (Symptom* or Hot flush or hot flash or hot flushes or vasomotor or Psychological or Locomotor or Urogenital or Somatic or Night sweats or Vaginal dryness or Loss of sex drive or Less sexual feelings or libido or incontinence or Urinary frequency or Bone thinning or osteoporosis or Low mood or Mood changes or Fatigue or Insomnia or Palpitations or Cognitive symptoms or Brain fog or Mental fog or Depress* or Anxi* or Lightheaded or Headache* or Irritab* or Anxiety or Sleeplessness or (sleep adj5 problem*) or (sleep adj5 difficult*) or Backache or Joint pains or Muscle pains or Pain or New facial hair or Dry skin or Dry vagina or vaginal atrophy).mp., **5759968**, Advanced

3, (Troublesome or problem* or sever* or bothersome or acute or disruptive or ((poor or low or decreased or adverse) adj5 quality of life) or Impact on daily routine).mp., **8089843**, Advanced

4, (factor* or demographic* or social class or socioeconomic or deprivation or ethnicity or characteristic* or risk or determinant or predict* or associat* or ((hazard or risk or odds) adj5 ratio)).mp., **17008001**, Advanced

5, (postmenopaus* or menopaus* or perimenopaus*).ti,ab,kf., 157136, Advanced

6, 1 and 2 and 3 and 4 and 5, **2507**, Advanced

*CINAHL Search:*

*Cochrane database of RCTs:*

Table S1: A list of excluded studies and reasons

| **Study** | **Title** | **Reason for exclusion** | **Links** |
| --- | --- | --- | --- |
| Abdel-Salam 2021 | Postmenopausal Symptoms and Their Correlates among Saudi Women Attending Different Primary Health Centers | exposure/outcome categories | https://doi.org/10.3390/ijerph18136831 |
| Abdullah 2017 | Prevalence of menopausal symptoms, its effect on quality of life among Malaysian women and their treatment seeking behaviour | lack of symptom-specific statistics | https://doi.org/10.4103/jfmpc.jfmpc_1377_22 |
| Abou-Raya 2016 | Relationship between sociodemographic, reproductive, and lifestyle factors and the severity of menopausal symptoms among Egyptian women in Alexandria | lack of symptom-specific statistics | https://doi.org/10.1097/GME.0000000000000635 |
| Abshirini 2018 | Dietary to talantioxidant capacity is inversely related to menopausal symptoms:across-sectional study among Iranian postmenopausal women | lack of symptom-specific statistics | https://doi.org/10.1016/j.nut.2018.04.014 |
| Adbelaziz 2022 | The relationship between sleep quality and menopausal symptoms among postmenopausal women in Saudi Arabia | lack of symptom-specific statistics | https://doi: 10.15537/smj.2022.43.4.20210682 |
| Adhikari 2021 | Menopausal symptoms among middle-aged women and care providers’ readiness to deliver menopausal services: an observational study in Kavrepalanchok, Nepal | lack of symptom-specific statistics | https://doi.org/10.1080/26410397.2022.2141255 |
| Agaba 2017 | Prevalence and predictors of severe menopause symptoms among HIVpositive and -negative Nigerian women | lack of symptom-specific statistics | DOI: 10.1177/0956462417704778 |
| Agajani 2011 | Age at menopause and measuring symptoms at midlife in a community in Babol, Iran | lack of symptom-specific statistics | DOI: 10.1097/gme.0b013e31821a7a3a |
| AlDughaither 2015 | Menopausal symptoms and quality of life among Saudi women visiting primary care clinics in Riyadh, Saudi Arabia | lack of symptom-specific statistics | http://dx.doi.org/10.2147/IJWH.S84709 |
| AlQuaiz 2017 | Factors associated with different symptom domains among postmenopausal Saudi women in Riyadh, Saudi Arabia | incompatible reference groups | https://doi.org/10.1097/gme.0000000000000931 |
| Alam 2020 | The prevalence and associated factors of depression during pre-, peri-, and post-menopausal period among the middle-aged women of Dhaka city | lack of symptom-specific statistics | https://doi.org/10.1016/j.ajp.2020.102312 |
| Ali 2014 | Does the experience of menopause differ among Hispanic women based on level of acculturation? | lack of symptom-specific statistics | http://journals.lww.com/menopausejournal |
| Aloufi 2022 | The Association of Menopausal Symptoms and Social Support Among Saudi Women at Primary Health Care Centers in Taif, Saudi Arabia | lack of symptom-specific statistics | https://doi.org/10.7759/cureus.26122 |
| Alquaiz 2014 | Determinants of severity of menopausal symptoms among Saudi women in Riyadh city | lack of symptom-specific statistics | https://doi.org/10.3109/13697137.2013.815161 |
| Alshogran 2022 | Predictors of age at menopause and psychiatric symptoms among postmenopausal females in Jordan | lack of symptom-specific statistics | DOI: 10.1080/0167482X.2021.1984425 |
| An 2023 | Urban-rural differences in epidemiology and risk factors of menopause syndrome in middle-aged Chinese women | lack of symptom-specific statistics | DOI: 10.1097/GME.0000000000002135 |
| Appling 2007 | Ethnicity and Vasomotor Symptoms in Postmenopausal Women | exposure/outcome categories | DOI: 10.1089/jwh.2006.0033 |
| Askin 2019 | Relationship between Postmenopausal Vitamin D Level, Menopausal Symptoms and Sexual Functions | lack of symptom-specific statistics | https://doi.org/10.29271/jcpsp.2019.09.823 |
| Augoulea 2019 | Association of menopausal symptoms with sociodemographic factors and personality traits | exposure/outcome categories | https://doi.org/10.5114/pm.2019.93117 |
| Azizi 2019 | Depressive symptoms and associated factors among Iranian women at midlife: a community-based, cross-sectional study | lack of symptom-specific statistics | DOI: 10.1097/GME.0000000000001374 |
| Banskota 2022 | Associations Between Menopausal Symptoms with Socio-economic, Demographic and Lifestyle Variables among Postmenopausal Women of Assam, Northeast India | lack of symptom-specific statistics | https://www.ojhas.org/issue84/2022-4-3.html |
| Barazzetti 2016 | Psychiatric disorders and menopause symptoms in Brazilian women | lack of symptom-specific statistics | DOI: 10.1097/GME.0000000000000548 |
| Barghandan 2021 | Association of depression, anxiety and menopausal‑related symptoms with demographic, anthropometric and body composition indices in healthy postmenopausal women | exposure/outcome categories | https://doi.org/10.1186/s12905-021-01338-w |
| Beas 2021 | Severity of climacteric sumptoms among Peruvian women from an urban coastal community and a rural Andean community | lack of symptom-specific statistics | https://doi.org/10.22605/RRH6457 |
| Beezhold 2016 | Female Vegans Report Lower Severity of Menopausal Symptoms than Omnivores | lack of symptom-specific statistics | abstract |
| Bener 2016 | Depression, Anxiety, and Stress Symptoms in  Menopausal Arab Women: Shedding More Light on a Complex Relationship | exposure/outcome categories | https://doi.org/10.4103/amhsr.amhsr_341_15 |
| Bermingham 2023 | Obesity is associated with greater prevalence of perimenopausal symptoms: findings from the ZOE Health study in 25,570 participants | lack of symptom-specific statistics | https://doi.org/10.1159/000530786 |
| Blumel 2011 | Prevalence, Duration and Impact Over Quality of Life of Vasomotor Symptoms In Mid-Aged Women of a Large Multinational Study | lack of symptom-specific statistics | 10.3109/13697137.2011.583733 |
| Blumel 2012 | A multinational study of sleep disorders during female mid-life | lack of symptom-specific statistics | http://dx.doi.org/10.1016/j.maturitas.2012.05.011 |
| Blumel 2015 | Obesity and its relation to depressive symptoms and sedentary lifestyle in middle-aged women | exposure/outcome categories | http://dx.doi.org/10.1016/j.maturitas.2014.10.007 |
| Blumel 2016 | Sedentary lifestyle in middle-aged women is associated with severe menopausal symptoms and obesity | lack of symptom-specific statistics | http://dx.doi.org/10.1097/GME.0000000000000575 |
| Blumstein 2012 | Cultural/ethnic differences in the prevalence of depressive symptoms among middle-aged women in Israel: the Women’s Health at Midlife Study | exposure/outcome categories | https://doi.org/10.1097/gme.0b013e318258b1dd |
| Bono 1995 | Factors associated with pain complaints in a clinical sample of postmenopausal women | exposure/outcome categories | https://doi.org/10.3109/01674829509024460 |
| Borkoles 2015 | Relationship between Type-D Personality, Physical Activity Behaviour and Climacteric Symptoms | exposure/outcome categories | https://doi.org/10.1186/s12905-015-0176-3 |
| Bosworth 2001 | Depressive Symptoms, Menopausal Status, and Climacteric Symptoms in Women at Midlife | exposure/outcome categories | DOI: [10.1017/S002966512200143410.1097](http://dx.doi.org/10.1017/S002966512200143410.1097)  /00006842-200107000-00013 |
| Brown D 2009 | Do Japanese American Women Really Have Fewer Hot Flashes than European Americans? The Hilo Women's Health Study | lack of symptom-specific statistics | https://doi.org/10.1097/gme.0b013e31819d88da |
| Brown J 2009 | Relations among Menopausal Symptoms, Sleep Disturbance and Depressive Symptoms in Midlife | exposure/outcome categories | https://dx.doi.org/10.1016/j.maturitas.2008.11.019 |
| Brzyski 2001 | Quality of life in low-income menopausal women attending primary care clinics | lack of symptom-specific statistics | https://doi.org/10.1016/s0015-0282(01)01852-0 |
| Cabral 2013 | Determinants of sexual dysfunction among middle-aged women | lack of symptom-specific statistics | https://dx.doi.org/10.1016/j.ijgo.2012.09.023 |
| Cagnacci 2006 | The relation of season of birth to severity of  menopausal symptoms | exposure/outcome categories | 10.1080/10.1097/01.gme.0000196594.82452.83 |
| Cagnacci 2017 | Association between urinary incontinence and climacteric symptoms in postmenopausal women | lack of symptom-specific statistics | DOI: 10.1097/GME.0000000000000727 |
| Carpenter 2021 | A Menopause Strategies–Finding Lasting Answers for Symptoms and Health (MsFLASH) Investigation of Self-Reported Menopausal Palpitation Distress | lack of symptom-specific statistics | https://doi.org/10.1089/jwh.2020.8586 |
| Chan 1995 | A study on menopausal symptoms and health needs among middle aged women | lack of symptom-specific statistics |  |
| Chang 2013 | Menopausal symptoms and physical activity in multiethnic groups of midlife women: a secondary analysis | lack of symptom-specific statistics | https://dx.doi.org/10.1111/jan.12056 |
| Chang 2014 | Effects of the body-mass index on menopausal symptoms among Asian American midlife women using two different classification systems | lack of symptom-specific statistics | https://doi.org/10.1111/1552-6909.12261 |
| Chedraui 2007 | Assessing menopausal symptoms among healthy middle aged women with the Menopause Rating Scale | lack of symptom-specific statistics | <https://doi.org/10.1016/j.maturitas.2007.01.009> |
| Chedraui 2007 | Menopausal symptoms and associated risk factors among postmenopausal women screened for the metabolic syndrome. | exposure/outcome categories | <https://doi.org/10.1007/s00404-006-0239-7> |
| Chedraui 2008 | Impaired quality of life among middle aged women: a multicentre Latin American study. | lack of symptom-specific statistics | <https://doi.org/10.1016/j.maturitas.2008.09.026> |
| Chedraui 2010 | Severe menopausal symptoms in middle-aged women are associated to female and male factors | exposure/outcome categories | https://doi.org/10.1007/s00404-009-1204-z |
| Chedraui 2014 | Application of the 10 item Cervantes Scale among mid aged Ecuadorian women for the assessment of menopausal symptoms | lack of symptom-specific statistics | 10.1016/j.maturitas.2014.06.019 |
| Chedraui 2014 | Evaluation of the presence and severity of menopausal symptoms among postmenopausal women screened for the metabolic syndrome | lack of symptom-specific statistics | http://dx.doi.org/10.3109/09513590.2014.971236 |
| Chee 2016 | The Covariates of Menopausal Symptoms in Four Major Racial/Ethnic Groups in the U.S. | lack of symptom-specific statistics | abstract |
| Chen 2010 | Acomparative study of menopausal hot flashes and their psychosocial correlates in Taiwan and the United States | incompatible reference groups | https://doi.org/10.1016/j.maturitas.2010.06.013 |
| Chim 2002 | The prevalence of menopausal symptoms in a community in Singapore | incompatible reference groups | https://doi.org/10.1016/s0378-5122(01)00299-7 |
| Choijamts 2022 | Impact of perimenopausal symptomatology on  quality of life in Mongolian women | lack of symptom-specific statistics | https://doi.org/10.1080/01443615.2022.2106829 |
| Chopra 2022 | A cross sectional survey of 504 women regarding perceived risk factors and barriers to follow healthy lifestyle and association with sociodemographic factors and menopausal symptoms | lack of symptom-specific statistics | https://doi.org/10.1016/j.dsx.2022.102529 |
| Chu 2022 | Biopsychosocial risk factors of depression during menopause transition in southeast China | exposure/outcome categories | https://doi.org/10.1186/s12905-022-01710-4 |
| Costa 2022 | Does Obesity Aggravate Climacteric Symptoms in Postmenopausal Women? | exposure/outcome categories | https://dx.doi.org/10.1055/s-0042-1745789 |
| Costa-Paiva 2016 | Association Between Obesity and Menopausal Symptoms Evaluated by Menopause Rating Scale: A Populational Study | lack of symptom-specific statistics | <https://doi.org/10.1097/GME.0000000000000783> |
| D'souza 2021 | The association between physical activity and menopause-related quality of life | lack of symptom-specific statistics | https://doi.org/10.12968/ijtr.2020.0118 |
| DaFonseca 2013 | Impact of age and body mass on the intensity ofmenopausal symptoms in 5968 Brazilian women | lack of symptom-specific statistics | https://doi.org/10.3109/09513590.2012.730570 |
| Dabrowska-Galas 2019 | High Physical Activity Level May Reduce Menopausal Symptoms | exposure/outcome categories | doi:10.3390/medicina55080466 |
| Dasgupta 2015 | Vasomotor and urogenital problems at midlife: a study on rural and urban women in India | incompatible reference groups | DOI: 10.3109/03014460.2014.941397 |
| David 2018 | Vasomotorsymptomsinwomenover60:resultsfromtheDataRegistry on Experiences of Aging, Menopause, and Sexuality (DREAMS) | wrong patient population | DOI: 10.1097/GME.0000000000001126 |
| Dehghan 2022 | Menopause symptoms in women and its relation with using complementary and alternative medicines: A survey in southeast Iran | lack of symptom-specific statistics | DOI 10.3389/fpubh.2022.947061 |
| Dennerstein 1994 | Sexuality and the menopause. | incompatible reference groups | https://doi.org/10.3109/01674829409025630 |
| Dhanoya 2016 | Hot flushes and reproductive hormone levels during the menopausal transition | incompatible reference groups | http://dx.doi.org/10.1016/j.maturitas.2016.03.017 |
| Dotlic 2018 | Factors associated with general and health-related quality of life in menopausal transition among women from Serbia | lack of symptom-specific statistics | https://doi.org/10.1080/03630242.2017.1306604 |
| Dotlic 2023 | Patterns of smoking and menopause-specific quality of life: smoking duration matters more | lack of symptom-specific statistics | https://doi.org/10.1080/08964289.2021.1958739 |
| Durairaj 2022 | Determinants of Menopausal Symptoms and Attitude Towards Menopause Among Midlife Women: A Cross-Sectional Study in South India | lack of symptom-specific statistics | DOI: 10.7759/cureus.28718 |
| Eftekhar 2016 | Female Sexual Function During the Menopausal Transition in a Group of Iranian Women | lack of symptom-specific statistics | https://pmc.ncbi.nlm.nih.gov/articles/PMC5026668/ |
| ElHajj 2020 | Menopausalsymptoms,physical activity level andquality of life of women living in the Mediterranean region | lack of symptom-specific statistics | https://doi.org/10.1371/journal.pone.0230515 |
| ElShafie 2011 | Menopausal symptoms among healthy, middle-aged Omani women as assessed with the Menopause Rating Scale | lack of symptom-specific statistics | https://doi.org/10.1097/gme.0b013e31821b82ee |
| Eun-Oklm 2020 | The association of depressive symptoms to sleep-related symptoms during menopausal transition: racial/ethnic differences | exposure/outcome categories | https://dx.doi.org/10.1097/GME.0000000000001611 |
| Faubion 2012 | Caffeine and menopausal symptoms: what is the association? | exposure/outcome categories | https://doi.org/10.1097/gme.0000000000000301 |
| Fernandez-Alonso 2010 | Obesity is related to increased menopausal symptoms amongSpanish women | lack of symptom-specific statistics | DOI: 10.1258/mi.2010.010029 |
| Freeman 2001 | Symptom reports from a cohort of African American and white women in the late reproductive years | lack of symptom-specific statistics | http://dx.doi.org/10.1097/00042192-200101000-00007 |
| Freeman 2006 | Associations of Hormones and Menopausal Status With Depressed Mood in Women With No History of Depression | exposure/outcome categories | 10.1001/archpsyc.63.4.375 |
| Gazibara 2022 | A comparative study of climacteric symptoms among two populations of mid-aged women | exposure/outcome categories | DOI: 10.1080/01443615.2022.2035334 |
| Gharaibeh 2010 | Severity of menopausal symptoms of Jordanian women. | lack of symptom-specific statistics | 10.3109/13697130903050009 |
| Ghazanfarpour 2015 | The relationship between women’s attitude  towards menopause and menopausal symptoms  among postmenopausal women | exposure/outcome categories | https://doi.org/10.3109/09513590.2015.1056138 |
| Ghazanfarpour 2016 | Investigating the Relationship Between Sexual Function and Quality of Life in Menopausal Women | lack of symptom-specific statistics | Investigating the Relationship Between Sexual Function and Quality of Life in Menopausal Women - PMC |
| Gibbs 2013 | Factors Associated with Depression During the Perimenopausal Transition | exposure/outcome categories | http://dx.doi.org/10.1016/j.whi.2013.07.001 |
| Goncalves 2016 | Overweight and obesity and factors associated with menopause | exposure/outcome categories | 10.1590/1413-81232015214.16552015 |
| Goonaratna 1999 | Perimenopausal symptoms in Sri Lankan women. | lack of symptom-specific statistics | scanned PDF no link |
| Green 2009 | Menopausal symptoms and ethnicity: the Study of Women’s Health Across the Nation | lack of symptom-specific statistics | https://doi.org/10.2217/17455057.5.2.127 |
| Grigoriou 2013 | Prevalence of vasomotor, psychological, psychosomatic and sexual symptoms in perimenopausal and recently postmenopausal Greek women: association with demographic, life style and hormonal factors | lack of symptom-specific statistics | https://doi.org/10.3109/09513590.2012.708801 |
| Grochans 2018 | Analysis of Sociodemographic, Psychological,  and Genetic Factors Contributing to Depressive  symptoms in Pre-, Peri- and Postmenopausal Women | exposure/outcome categories | http://dx.doi.org/10.3390/ijerph15040712 |
| Gupta 2009 | Mid-Aged Health in Women from the Indian Subcontinent (MAHWIS): a further quantitative and qualitative investigation of experience of menopause in UK Asian women, compared to UK Caucasian women and women living in Delhi | incompatible reference groups | https://doi.org/10.1080/13697130802556304 |
| Hafiz 2007 | A quantitative analysis of the menopause experience of Indian women living in Sydney | lack of symptom-specific statistics | https://doi.org/10.1111/j.1479-828X.2007.00746.x |
| Haimov-Kochman 2013 | Regular exercise is the most significant lifestyle parameter associated with the severity of climacteric symptoms: a cross sectional study | lack of symptom-specific statistics | http://dx.doi.org/10.1016/j.ejogrb.2013.06.018 |
| Hariton 2022 | Racial and Ethnic Differences in Menopausal Symptoms: A Cross sectional Study of 44,678 Women | lack of symptom-specific statistics | 10.1097/GME.0000000000002112 |
| Hartz 2013 | Somatic symptoms and psychological characteristics associated with insomnia in postmenopausal women | exposure/outcome categories | http://dx.doi.org/10.1016/j.sleep.2012.08.003 |
| Huang 2023 | Depressive symptoms and physical activity  among community-dwelling perimenopausal  women: a prospective longitudinal study | exposure/outcome categories | https://doi.org/10.1186/s12888-023-04591-5 |
| Ibrahim 2020 | The effect of menopausal symptoms on the quality of life among postmenopausal Egyptian women | lack of symptom-specific statistics | https://doi.org/10.1080/13697137.2019.1656185 |
| Im 2009 | An Internet survey on ethnic differences in menopausal symptoms and contextual factors | lack of symptom-specific statistics | https://doi.org/10.1080/07399330802695002 |
| Im 2010_2 | Sub-Ethnic Differences in the Menopausal Symptom Experience: Asian American Midlife Women | lack of symptom-specific statistics | https://doi.org/10.1177/1043659609357639 |
| Im 2014 | Physical Activities and Sleep-Related Symptoms in 4 Major Racial/Ethnic Groups of Midlife Women | exposure/outcome categories | https://www.jstor.org/stable/10.2307/48515393 |
| Im 2015 | Cluster analysis of midlife women’s sleep-related symptoms: racial/ethnic differences | exposure/outcome categories | https://doi.org/10.1097/GME.0000000000000460 |
| Im 2016 | A cluster analysis of cardiovascular symptoms experienced during menopausal transition | exposure/outcome categories | https://doi.org/10.1097/GME.0000000000000783 |
| Im 2020 | The association of depressive symptoms to sleep-related symptoms during menopausal transition: racial/ethnic differences | exposure/outcome categories | https://doi.org/10.1097/GME.0000000000001611 |
| Im 2021 | A decision tree analysis on multiple factors related to menopausal symptoms. | lack of symptom-specific statistics | DOI: 10.1097/GME.0000000000001798 |
| Im 2021 | Depressive symptoms and type II diabetes mellitus among midlife women. | exposure/outcome categories | DOI: 10.1097/GME.0000000000001759 |
| Ivarsson 1998 | Physical exercise and vasomotor symptoms in postmenopausal women | incompatible reference groups | https://doi.org/10.1016/s0378-5122(98)00004-8 |
| Iwanowicz-Palus 2013 | Determinant factors of health in rural women in their perimenopausal period | exposure/outcome categories | <https://www.aaem.pl/Determinant-factors-of-health-in-rural-women>  -in-their-perimenopausal-period,71896,0,2.html |
| Jaber 2017 | Patterns and severity of menopausal symptoms among Jordanian women | exposure/outcome categories | https://doi.org/10.1080/08952841.2016.1213110 |
| Jaffe 2008 | [Commentary on] Persistent hot flushes in older postmenopausal women. | incompatible reference groups | https://doi.org/10.1001/archinte.168.8.840 |
| Jalal 2020 | Physical Activity, Self-Care, and Menopausal Symptoms among WomeninAl-Ahsa, Saudi Arabia: Adherence to Postmenopausal Guidelines (PMGs) | lack of symptom-specific statistics | https://doi.org/10.3390/healthcare12090886 |
| Joseph 2014 | Assessment of menopausal symptoms among women attending various outreach clinics in South Canara District of India | lack of symptom-specific statistics | https://doi.org/10.4103/0976-7800.133996 |
| Kahlout 2023 | Investigating the Prevalence and Severity of Menopausal Symptoms and Quality of Life in Peri-menopausal and Post-menopausal Palestinian  Women: A Cross-Sectional Study | lack of symptom-specific statistics | http://dx.doi.org/10.30476/WHB.2022.96809.1194 |
| Kamal 2017 | Quality of life among postmenopausal women in  rural Minia, Egypt | lack of symptom-specific statistics | https://doi.org/10.26719/2017.23.8.527 |
| Karacam 2007 | Factors associated with menopausal symptoms and their relationship with the quality of life among Turkish women | exposure/outcome categories | http://dx.doi.org/10.1016/j.maturitas.2007.06.004 |
| Katainen 2015 | A population-based survey of quality of life in middle-aged Finnish women | exposure/outcome categories | https://doi.org/10.1097/gme.0000000000000335 |
| Kazama 2023 | Associations of fat mass index with hot flashes and lean mass index with insomnia in middle-aged women. | exposure/outcome categories | https://doi.org/10.1080/13697137.2023.2173566 |
| Khandelwal 2016 | Indian menopausal women health characteristics and strategies of interdisciplinary care | lack of symptom-specific statistics | abstract |
| Kim 2020 | Associations Among BMI, Dietary Macronutrient Consumption, and Climacteric Symptoms in Korean Menopausal Women | exposure/outcome categories | https://doi.org/10.3390/nu12040945 |
| Kingsberg 2022 | Global View of Vasomotor Symptoms and Sleep Disturbance in Menopause: A Systematic Review | lack of symptom-specific statistics | https://doi.org/10.1080/13697137.2023.2256658 |
| Kirchengast 1992 | Effect of socioeconomic factors on timing of menopause and the course of climacteric. | lack of symptom-specific statistics | scanned PDF no link |
| Kling 2019 | Association between menopausal symptoms and relationship distress. | exposure/outcome categories | https://doi.org/10.1016/j.maturitas.2019.09.006 |
| Kocak 2023 | Modeling the risk factors of women with very severe menopausal symptoms | lack of symptom-specific statistics | https://doi.org/10.1111/jog.15556 |
| Kochersberger 2024 | The association of race, ethnicity, and socioeconomic status on the severity of menopause symptoms: a study of 68,864 women | incompatible reference groups | 10.1097/GME.0000000000002349 |
| Kopanos 2021 | The severity of menopausal symptoms is associated with the pres- ence of diabetes mellitus and adiposity | lack of symptom-specific statistics | http://dx.doi.org/10.1016/j.maturitas.2021.08.036 |
| Kravitz 2003 | Sleep difficulty in women at midlife: a community survey of sleep and the menopausal transition | exposure/outcome categories | 10.1097/01.GME.0000030707.35686.5A |
| Kravitz 2015 | An actigraphy study of sleep and pain in midlife women: the Study of Women’s Health Across the Nation Sleep Study | exposure/outcome categories | https://doi.org/10.1097/gme.0000000000000379 |
| Kumari 2020 | Impaired Quality of Life and Its Determinants among Postmenopausal Women of Slum Communities in Bhubaneswar, India | exposure/outcome categories | http://dx.doi.org/10.4103/jmh.JMH_111_19 |
| Kwon 2022 | Alcohol Consumption Patterns and Risk of Early-Onset Vasomotor Symptoms in Premenopausal Women. | incompatible reference groups | https://doi.org/10.3390/nu14112276 |
| Lee 2008 | Relationships between menopausal symptoms,  depression, and exercise in middle-aged women:  A cross-sectional survey | lack of symptom-specific statistics | https://doi.org/10.1016/j.ijnurstu.2008.07.001 |
| Lee 2010 | Factors influencing the severity of menopause symptoms in Korean post-menopausal women. | exposure/outcome categories | 10.3346/jkms.2010.25.5.758 |
| Lee 2012 | Association between menopausal symptoms and metabolic syndrome in postmenopausal women | exposure/outcome categories | https://doi.org/10.1007/s00404-011-2016-5 |
| Lee 2013 | Muscle strength and quality are associated with severity of menopausal symptoms in peri-  and post-menopausal women | exposure/outcome categories | http://dx.doi.org/10.1016/j.maturitas.2013.06.007 |
| Lee 2020 | Prevalence of symptoms and associated factors across menopause status in Taiwanese women | exposure/outcome categories | https://doi.org/10.1097/GME.0000000000001662 |
| Legorreta 2013 | Age at menopause, motives for consultation and symptoms reported by 40–59-year-old Mexican women | exposure/outcome categories | https://doi.org/10.3109/13697137.2012.696288 |
| Lermer 2011 | Somatic and affective anxiety symptoms and menopausal hot flashes | exposure/outcome categories | https://doi.org/10.1097/gme.0b013e3181ec58f8 |
| Li 2005 | Background factors influencing somatic and psychological symptoms in middle-age women with different hormonal status Apopulation-based study of Swedish women | lack of symptom-specific statistics | https://doi.org/10.1016/j.maturitas.2005.05.005 |
| Li 2012 | Factors associated with the age of natural menopause and menopausal symptoms in Chinese women | exposure/outcome categories | http://dx.doi.org/10.1016/j.maturitas.2012.09.008 |
| Li 2013 | Survey on epidemiologic factors associated with the age of natural menopause and menopausal symptoms in Jiangsu women | lack of symptom-specific statistics | <https://doi.org/10.1016/j.maturitas.2012.09.008> |
| Li 2016 | Perimenopausal syndrome and mood disorders in perimenopause: prevalence, severity, relationships, and risk factors | exposure/outcome categories | http://dx.doi.org/10.1097/MD.0000000000004466 |
| Li 2021 | Individual and additive-effect relationships of sleep problems and severe menopausal symptoms among women in menopausal transition | lack of symptom-specific statistics | http://dx.doi.org/10.1097/GME.0000000000001726 |
| Lipasti 2021 | Climacteric symptoms more severe in 2010 than in 2000 – experience of  Finnish women aged 52–56 years not now or previously on menopausal  hormone therapy | lack of symptom-specific statistics | https://doi.org/10.1016/j.maturitas.2021.09.003 |
| Liu 2008 | The menopausal experience of Greek women living in Sydney | exposure/outcome categories | https://doi.org/10.1097/gme.0b013e318154bda0 |
| Logan 2023 | Menopausal symptoms in midlife Singaporean women: Prevalence rates and associated factors from the Integrated Women's Health Programme (IWHP). | exposure/outcome categories | https://doi.org/10.1016/j.maturitas.2023.107853 |
| Love 2022 | Identifying factors that predict menopause symptoms to support personalised care pathways | lack of symptom-specific statistics | The Journal of The North American Menopause Society,  Volume 29, Issue 12, page 1475 (December 2022). |
| Lu 2007 | The experience of menopausal symptoms by Arabic women in Sydney | exposure/outcome categories | https://doi.org/10.1080/13697130601159649 |
| Lu 2023 | Prevalence of menopausal symptoms and attitudes towards menopausal hormone therapy in women aged 40-60 years: a cross-sectional study. | exposure/outcome categories | https://doi.org/10.1186/s12905-023-02621-8 |
| Lui-Filho 2018 | Risk factors associated with intensity of climacteric symptoms in Brazilian middle-aged women: a population-based household survey. | exposure/outcome categories | DOI: 10.1097/GME.0000000000001022 |
| Lui-Filho 2019 | Risk factors associated with genitourinary syndrome of menopause in brazilian middle-aged women: a population-based household survey | lack of symptom-specific statistics | not available |
| LuiFilho 2015 | Epidemiologia da menopausa e dos sintomas  climatéricos em mulheres de uma região  metropolitana no sudeste do Brasil:  inquérito populacional domiciliar | lack of symptom-specific statistics | https://doi.org/10.1590/SO100-720320150005282 |
| Martinez 2013 | Urban-rural differences in Spanish menopausal  women | lack of symptom-specific statistics | http://www.rrh.org.au |
| Mathew 2020 | A Cross‑Sectional Study to Assess the Quality of Life of Perimenopausal and Post menopausal Women in Rural Etawah, Uttar Pradesh, India | incompatible reference groups | https://doi.org/10.4103/jmh.JMH_88_19 |
| Matsuo 2012 | Comparison of Menopause Healthcare Considerations  between Japanese and Filipino Women Living in Local  Communities | incompatible reference groups | https://pubmed.ncbi.nlm.nih.gov/23660453/ |
| Matthews 2019 | Racial/ethnic disparities in women's sleep duration, continuity, and quality, and their statistical mediators: Study of Women's Health Across the Nation. | exposure/outcome categories | doi: 10.1093/sleep/zsz042 |
| Matthews 2021 | Identifying women who share patterns of reproductive hormones,  vasomotor symptoms, and sleep maintenance problems across the  menopause transition: group-based multi-trajectory modeling in the  Study of Women’s Health Across the Nation | exposure/outcome categories | https://doi.org/10.1097/GME.0000000000001663 |
| Metintas 2010 | Menopause Rating Scale as a screening tool in  rural Turkey | lack of symptom-specific statistics | https://pubmed.ncbi.nlm.nih.gov/20356316/ |
| Misiker 2023 | The severity of menopause and associated  factors among middle-aged women residing  in Arba Minch, DHSS, Ethiopia, 2022 | lack of symptom-specific statistics | https://doi.org/10.1186/s12905-023-02442-9 |
| Mitchell 2010 | Pain symptoms during the menopausal transition and early postmenopause | exposure/outcome categories | https://doi.org/10.3109/13697137.2010.483025 |
| Mitchell 2015 | Hot flush severity during the menopausal  transition and early postmenopause: beyond  hormones | exposure/outcome categories | https://doi.org/10.3109/13697137.2015.1009436 |
| Mitchell 2017 | Depressed mood during the menopausal transition: is it reproductive aging or is it life? | exposure/outcome categories | https://doi.org/10.1186/s40695-017-0030-x |
| Moilanen 2010 | Prevalence of Menopause Symptoms and their Association with Lifestyle Among Finnish middle-aged women | exposure/outcome categories | 10.1016/j.maturitas.2010.08.007 |
| Monica 2011 | body mass index and hot flashes in postmenopausal women: a meta-analysis | lack of symptom-specific statistics | https://doi.org/10.3109/13697137.2011.583733 |
| Monterrosa 2008 | Increased menopausal symptoms among Afro-Colombian  women as assessed with the Menopause Rating Scale | exposure/outcome categories | https://doi.org/10.1016/j.maturitas.2007.12.002 |
| Monterrosa 2009 | Quality of life impairment among postmenopausal women varies according to race | exposure/outcome categories | https://doi.org/10.1080/09513590902972091 |
| Monterrosa-Castro 2012 | Assessment of sleep quality and correlates in a large cohort of Colombian women around menopause | exposure/outcome categories | http://dx.doi.org/10.1097/gme.0b013e31826e7649 |
| Monterrosa-Castro 2013 | Assessment of sleep quality and correlates in a large cohort of Colombian women around menopause | exposure/outcome categories | https://doi.org/10.1097/gme.0b013e31826e7649 |
| Monterrosa-Castro 2013_2 | Prevalence of insomnia and related factors in a large mid-aged female Colombian sample | exposure/outcome categories | http://dx.doi.org/10.1016/j.maturitas.2013.01.009 |
| Monterrosa-Castro 2016 | Differential Item Functioning of the Psychological Domain of the Menopause Rating Scale. | exposure/outcome categories | http://dx.doi.org/10.1155/2016/8790691 |
| Morse 2019 | Exercise and Insomnia Risk in Middle-Aged Women | exposure/outcome categories | https://doi.org/10.1016/j.nurpra.2018.10.020 |
| Muharam 2018 | Depression and its link to other symptoms in menopausal transition | exposure/outcome categories | http://dx.doi.org/10.1016/j.mefs.2017.08.003 |
| Murphy 2022 | Symptoms and health behaviours of post-menopausal women in Ireland | lack of symptom-specific statistics | https://doi.org/10.1017/S0029665122001422 |
| Murphy 2022_2 | Menopausal symptoms and adherence to a Mediterranean dietary pattern  in women living in Ireland | lack of symptom-specific statistics | http://dx.doi.org/10.1017/S0029665122001434 |
| Nagaraj 2021 | Experience and Perceptions Regarding Menopause among Rural  Women: A Cross‑Sectional Hospital‑Based Study in South Karnataka | exposure/outcome categories | https://doi.org/10.4103/jmh.JMH_196_20 |
| Nancy 2014 | Hot flash severity across the menopausal transition: observations from the seattle midlife women’s health study | lack of symptom-specific statistics | abstract published |
| Nazarpour 2018 | The relationship between menopausal symptoms and sexual function | exposure/outcome categories | https://doi.org/10.1080/03630242.2017.1414100 |
| Nazarpour 2020 | Factors associated with quality of life of postmenopausal women living in Iran. | exposure/outcome categories | https://doi.org/10.1186/s12905-020-00960-4 |
| Nelson 2011 | Effects of reproductive history on symptoms of menopause: a brief report | exposure/outcome categories | https://doi.org/10.1097/gme.0b013e318214d69d |
| Neri 1997 | Coping style and climacteric symptoms in a clinical sample of postmenopausal women. | exposure/outcome categories | https://doi.org/10.3109/01674829709080692 |
| Neutzling 2020 | Association between common mental disorders, sleep quality, and menopausal symptoms: a population-based study in Southern Brazil. | lack of symptom-specific statistics | DOI:10.1097/GME.0000000000001524 |
| Noll 2022 | Life habits of postmenopausal women: Association of menopause symptom  intensity and food consumption by degree of food processing | exposure/outcome categories | https://doi.org/10.1016/j.maturitas.2021.10.015 |
| Nowakowska 2015 | Analysis of relationships between perimenopausal symptoms and professional functioning and life satisfaction – subjective perception of the dependence in women aged 40+ | lack of symptom-specific statistics | http://dx.doi.org/10.13075/mp.5893.00243 |
| Ogwumike 2016 | Musculoskeletal pain among postmenopausal women in Nigeria:Association with overall and central obesity | exposure/outcome categories | http://dx.doi.org/10.1016/j.hkpj.2015.06.001 |
| Oi 2012 | A study of menopausal symptoms in relation to habits of smoking and make-up using in Japanese women aged 35–59 | exposure/outcome categories | http://dx.doi.org/10.1111/j.1468-2494.2012.00722.x |
| Ojeda 2010 | Severe menopausal symptoms in mid-aged Latin American women can be related to their indigenous ethnic component. | exposure/outcome categories | https://doi.org/10.3109/13697130903576297 |
| Ojeda 2011 | Severe menopausal symptoms in mid-aged Latin American women can be related to their indigenous ethnic component | exposure/outcome categories | https://doi.org/10.3109/13697130903576297 |
| Olajubu 2017 | Influence of menopausal symptoms on perceived work ability among women in a Nigerian University. | lack of symptom-specific statistics | https://doi.org/10.1080/13697137.2017.1373336 |
| Olowokere 2021 | Lifestyle practices and menopause-related symptoms among women in rural communities of Ado-Ekiti local government area, Nigeria. | lack of symptom-specific statistics | https://doi-org.ezproxy-prd.bodleian.ox.ac.uk/10.1177/2053369120971427 |
| Onya 2018 | Biological and environmental correlates of post menopausal depression among Gopd attendees in Fmc, Lokoja, Nigeria | lack of symptom-specific statistics | no full text available from library |
| Palacios 2010 | Age of menopause and impact of climacteric symptoms by geographical region | exposure/outcome categories | https://doi.org/10.3109/13697137.2010.507886 |
| Panichkul 2017 | Menopause-related symptoms and quality of life in  peri- and postmenopausal women in Thailand: a  multicenter study | exposure/outcome categories | https://doi.org/10.5372/1905-7415.1103.551 |
| Papazisis 2022 | Prevalence of post-menopausal depression and associated factors: A web-based cross-sectional study in Greece. | lack of symptom-specific statistics | https://doi.org/10.1016/j.maturitas.2021.10.014 |
| Park 2001 | A Survey on the Climacteric Symptoms in Korean Women | incompatible reference groups | https://doi.org/10.1300/J013v34n01_02 |
| Pastore 2004 | Self-reported urogenital symptoms in postmenopausal women: Women’s Health Initiative | incompatible reference groups | https://doi.org/10.1016/j.maturitas.2004.06.019 |
| Perez 2009 | Epidemiology of risk factors and symptoms associated with menopause in Spanish women | lack of symptom-specific statistics | https://doi.org/10.1016/j.maturitas.2008.10.003 |
| Perez-Roncero 2013 | Menopause-related symptoms in middle-aged women residing in the Zaragoza Province | lack of symptom-specific statistics | http://dx.doi.org/10.1016/j.enfcli.2013.09.003 |
| Pien 2008 | Predictors of Sleep Quality in Women in the Menopausal Transition | exposure/outcome categories | https://doi.org/10.5665/sleep/31.7.991 |
| Pimenta 2011 | Perceived control, lifestyle, health, socio-demographic factors and menopause: Impact on hot flashes and night sweats | lack of symptom-specific statistics | https://doi.org/10.1016/j.maturitas.2011.05.005 |
| Pimenta 2012 | Menopause Symptoms' Predictors: The Influence  of Lifestyle, Health- and Menopause-Related, and Sociodemographic Characteristics | exposure/outcome categories | https://doi.org/10.1080/08952841.2012.639653 |
| Pimenta 2012 | Menopausal symptoms: do life events predict severity of symptoms in peri and post menopause? | exposure/outcome categories | 10.1016/j.maturitas.2012.04.006 |
| Pimenta 2014 | Menopausal Symptoms: Is Spirituality Associated with the Severity of Symptoms? | exposure/outcome categories | https://doi.org/10.1007/s10943-013-9696-z |
| Prado 2008 | Assessment of quality of life using the Menopause Rating Scale in women aged 40 to 59 years | incompatible reference groups | https://pubmed.ncbi.nlm.nih.gov/19350167/ |
| Prairie 2011 | Symptoms of depressed mood, disturbed sleep and sexual problems in midlife women: cross-sectional data from the Study of Women's Health Across the Nation (SWAN) | exposure/outcome categories | 10.1089/jwh.2014.4798 |
| Prairie 2015 | Symptoms of depressed mood, disturbed sleep and sexual problems in midlife women: cross-sectional data from the Study of Women's Health Across the Nation | exposure/outcome categories | 10.1089/jwh.2014.4798 |
| Rahman 2020 | Impact of Body Mass Index (BMI) and Physical activities among menopausal women | incompatible reference groups | https://doi.org/10.3329/bmrcb.v46i2.49020 |
| Reed 2009 | Depressive symptoms and menopausal burden in the midlife | exposure/outcome categories | https://doi.org/10.1016/j.maturitas.2009.01.002 |
| Ri 2022 | Hypertensive disorders of pregnancy increase the risk of future menopausal hot flashes in Japanese women: results from the Japan Nurses’ Health Study | exposure/outcome categories | http://dx.doi.org/10.1097/GME.0000000000001889 |
| Rindner 2017 | Prevalence of somatic and urogenital symptoms as well as psychological health in women aged 45 to 55 attending primary health care: a cross-sectional study | lack of symptom-specific statistics | https://doi.org/10.1186/s12905-017-0480-1 |
| Rouen 2009 | Study of women veterans in menopause. | exposure/outcome categories | n/a (UMI: 3354092  ) |
| Sabia 2008 | Risk factors for onset of menopausal symptoms Results from a large cohort study | lack of symptom-specific statistics | https://doi.org/10.1016/j.maturitas.2008.04.004 |
| Saccomani 2017 | Does obesity increase the risk of hot flashes among midlife women?: a population-based study | exposure/outcome categories | https://doi.org/10.1097/GME.0000000000000884 |
| Salazar-Pousada 2017 | Evaluation of depressive symptoms in mid-aged women: report of a multicenter South American study | lack of symptom-specific statistics | https://doi.org/10.1097/GME.0000000000000924 |
| Sanchez-Zarza 2023 | Prevalence of menopausal symptoms and severity related factors among mid-aged Paraguayan women as measured with the 10-item Cervantes Scale. | exposure/outcome categories | https://doi.org/10.1080/09513590.2023.2235427 |
| Sayakhot 2012 | Cross-cultural study: experience, understanding of menopause, and related therapies in Australian and Laotian women | exposure/outcome categories | https://dx.doi.org/10.1097/gme.0b013e31825fd14e |
| Sayon Orea 2015 | Adherence to Mediterranean dietary pattern and menopausal symptoms in relation to overweight/obesity in Spanish perimenopsusal and postmenopausal women | exposure/outcome categories | DOI: 10.1097/GME.0000000000000378 |
| Schnatz 2006 | Menopausal Symptoms in Hispanic Women  and the Role of Socioeconomic Factors | exposure/outcome categories | 10.1097/01.ogx.0000201923.84932.90 |
| Sharami 2022 | The association between reproductive history and menopausal symptoms: an evidence from the cross-sectional survey. | lack of symptom-specific statistics | https://doi.org/10.1186/s12905-022-01715-z |
| SharanyaShre 2016 | Severity and clustering of menopausal symptoms among obese and non obese postmenopausal women in India | lack of symptom-specific statistics | 10.4103/0975-7406.171728 |
| Shea 2020 | Depression, hormone therapy, and the menopausal transition among women aged 45 to 64 years using Canadian Longitudinal Study on aging baseline data | exposure/outcome categories | https://doi.org/10.1097/GME.0000000000001540 |
| Sheereen 2022 | Community-based appraisal of menopause-specific health problems and quality of life among women of rural Western Maharashtra. | exposure/outcome categories | https://doi.org/10.4103/jfmpc.jfmpc_1377_22 |
| Shivappa 2016 | Association between inflammatory potential of diet and risk of depression in  middle-aged women: the Australian Longitudinal Study on Women’s Health | exposure/outcome categories | https://doi.org/10.1017/S0007114516002853 |
| Shukla 2018 | Prevalence of Postmenopausal Symptoms, Its Effect on Quality of Life and Coping in Rural Couple | incompatible reference groups | https://doi.org/10.4103/jmh.JMH_34_16 |
| Shyu 2012 | Health related quality of life and healthcare resource utilisation in Taiwanese women with menopausal symptoms: a nation-wide survey | incompatible reference groups | 10.1097/jnr.0b013e318265619b |
| Simpkins 2009 | Role of ethnicity in the expression of features of hot flashes | exposure/outcome categories | https://doi.org/10.1016/j.maturitas.2009.06.002 |
| Singhania 2020 | Association of Menopausal Symptoms with Overweight and Obesity among Rural Middle Aged Women in North India: A Population Based  Study | exposure/outcome categories | https://doi.org/10.4103/jmh.JMH_123_19 |
| Sitini 2023 | Prevalence, impact and management of postmenopausal symptoms among postmenopausal women in Rwanda | exposure/outcome categories | https://doi.org/10.1080/13697137.2023.2246887 |
| Skrzypulec 2010 | The influence of physical activity level on climacteric symptoms in menopausal women | lack of symptom-specific statistics | https://doi.org/10.3109/13697131003597019 |
| Smith 2018 | Understanding the complex relationships underlying hot flashes: a Bayesian network approach | incompatible reference groups | 10.1097/GME.0000000000000959 |
| Song 2022 | Menopausal Symptoms and Sleep Quality in Women Aged 40–65 Years | lack of symptom-specific statistics | https://doi.org/10.1155/2022/2560053 |
| Stojanovska 2014 | To exercise or not to exercise: Relationship between menopausal experience and exercise avoidance | incompatible reference groups | 10.1016/j.maturitas.2014.01.006 |
| Stojanovska 2015 | Menopause and avoidance of physical activity | incompatible reference groups | Page 1391 of abstracts |
| Su 2021 | Menopausal symptoms among Chinese and Japanese women: differences and similarities | exposure/outcome categories | https://doi.org/10.1097/GME.0000000000001874 |
| Su 2022 | Menopausal symptoms among Chinese and Japanese women: differences and similarities. | exposure/outcome categories | 10.1097/GME.0000000000001874 |
| Suau 2005 | Depressive symptoms and risk factors among perimenopausal women. | exposure/outcome categories |  |
| Sun 2014 | Sleep disturbance and correlates in menopausal women in Shanghai | exposure/outcome categories | http://dx.doi.org/10.1016/j.jpsychores.2013.12.002 |
| Sun 2021 | Association Between Parity and the Age at Menopause and Menopausal Syndrome in Northwest China. | lack of symptom-specific statistics | https://doi.org/10.1177/1010539520960987 |
| Sut 2017 | Severity of Menopausal Symptoms in Climacteric Turkish Women | lack of symptom-specific statistics | 42_sut_ABSTRACT_10_1 |
| Sweed 2012 | Postmenopausal symptoms among Egyptian geripausal women | lack of symptom-specific statistics | https://doi.org/10.26719/2012.18.3.213 |
| Takenaka 2021 | Investigation of Relationships among Health Literacy, Social Determinants of Health, Menopausal Symptoms, Lifestyle related Diseases and Depression in Menopausal Women | exposure/outcome categories | 10.1265/jjh.20004 |
| Tan 2014 | The effect of physical activity and body mass index on menopausal symptoms in Turkish women: a cross-sectional study in primary care | lack of symptom-specific statistics | http://www.biomedcentral.com/1472-6874/14/38 |
| Taskiran 2021 | Individual Characteristics Associated with Menopausal Symptom Severity and Menopause-Specific Quality of Life: A Rural Perspective | exposure/outcome categories | https://doi.org/10.1007/s43032-021-00545-y |
| Taylor 2010 | Do attitudes towards aging and menopause vary by menopausal status, age and symptoms? | exposure/outcome categories |  |
| Terauchi 2020 | Body Mass Index and Percentage Body Fat Are Negatively Associated with Severe Dyspareunia in Japanese Post-menopausal Women | lack of symptom-specific statistics | abstract |
| Terauchi 2022 | Body weight and body fat are negatively associated with severe dyspareunia in postmenopausal women | exposure/outcome categories | 10.1111/jog.15418 |
| Thapa 2020 | Menopausal symptoms and related factors among Cambodian women. | lack of symptom-specific statistics | https://doi.org/10.1080/03630242.2019.1643815 |
| Tong 2023 | High levels of physical activity are associated with a reduced likelihood of depressive symptoms in postmenopausal women. | exposure/outcome categories | https://doi.org/10.1080/03630242.2023.2188100 |
| Urrunaga-Pastor 2022 | Female orgasmic dysfunction and severe climacteric symptomatology in women aged 40 to 59years: an independent association from an  analysis of a multicenter Latin American study | exposure/outcome categories | http://dx.doi.org/10.1097/GME.0000000000001973 |
| Valadares 2016 | Is the age at menopause a cause of sexual dysfunction? A Brazilian population-based study | exposure/outcome categories | 10.1097/GME.0000000000000783 |
| Waidyasekera 2009 | Menopausal symptoms and quality of life during the menopausal transition in Sri Lankan women. | exposure/outcome categories | 10.1097/gme.0b013e31817a8abd |
| Wang 2013 | Depressive symptoms in Taiwanese women during the peri- and post-menopause years: Associations with demographic, health, and psychosocial characteristics | exposure/outcome categories | http://dx.doi.org/10.1016/j.maturitas.2013.04.021 |
| Wang 2021 | Severity and factors of menopausal  symptoms in middle-aged women in Gansu  Province of China: a cross-sectional study | lack of symptom-specific statistics | https://doi.org/10.1186/s12905-021-01531-x |
| Wang 2022 | Menopause-Related Symptoms and Influencing Factors in Mosuo, Yi, and Han Middle-Aged Women in China | exposure/outcome categories | https://doi.org/10.3389/fpsyg.2022.763596 |
| Wang 2022_2 | Association of menopausal status and symptoms with depressive symptoms in middle-aged Chinese women | exposure/outcome categories | https://doi.org/10.1080/13697137.2021.1998435 |
| Watson 2019 | Vulvovaginal Discomfort Is Common in Both  Premenopausal and Postmenopausal Women | exposure/outcome categories | https://doi.org/10.1097/LGT.0000000000000460 |
| Whiteley 2013 | The Impact of Menopausal Symptoms on Quality of Life, Productivity and Economic Outcomes | exposure/outcome categories | 10.1089/jwh.2012.3719 |
| Williams 2009 | Menopause-specific questionnaire assessment in US population-based study shows negative impact on health-related quality of life | lack of symptom-specific statistics | https://dx.doi.org/10.1016/j.maturitas.2008.12.006 |
| Wong 2014 | Prevalence and psychosocial correlates of depressive symptoms in urban Chinese women during midlife | exposure/outcome categories | 10.1371/journal.pone.0110877 |
| Woods 2022 | Perimenopause meets life: observations from the Women Living Better Survey | exposure/outcome categories | https://doi.org/10.1097/GME.0000000000002072 |
| Worsley 2017 | Moderate–Severe Vasomotor Symptoms Are Associated with Moderate–Severe Depressive Symptoms | exposure/outcome categories | https://doi.org/10.1089/jwh.2016.6142 |
| Wu 2023 | The relationship between physical activity and the severity of menopausal symptoms: a cross-sectional study. | lack of symptom-specific statistics | https://doi.org/10.1186/s12905-023-02347-7 |
| Xiong 2022 | Longitudinal associations between sleep quality and menopausal symptoms among community-dwelling climacteric women: A multi-centered study | lack of symptom-specific statistics | https://doi.org/10.1016/j.sleep.2022.08.025 |
| Yang 2008 | Menopausal symptoms in mid-life women in  Southern China | lack of symptom-specific statistics | https://doi.org/10.1080/13697130802239075 |
| Yanikkerem 2018 | Effects of physical and depressive symptoms on the sexual life of Turkish women in the climacteric period | exposure/outcome categories | https://doi.org/10.1080/13697137.2017.1417374 |
| Yim 2014 | Menopausal symptoms and associated factors in middle-aged women in Korea | lack of symptom-specific statistics | <https://scholarx.skku.edu/handle/2021.sw.skku/>  53368 |
| Yim 2015 | Prevalence and severity of menopause symptomsand associated factors across menopause status in Korean women | exposure/outcome categories | http://dx.doi.org/10.1097/GME.0000000000000438 |
| Yin 2015 | Association between polycystic ovary syndrome and hot flash presentation during the menopause transition | exposure/outcome categories |  |
| Yokota 2016 | Symptoms and effects of physical factors in Japanese middle-aged women | incompatible reference groups | https://doi.org/10.1097/GME.0000000000000660 |
| Yoshany 2020 | Association between Lifestyle and Severity of Menopausal Symptoms in Postmenopausal Women | exposure/outcome categories | https://doi.org/10.29333/ejgm/7885 |
| Zainal 2008 | Depressive Symptoms in Middle-Aged Women in Peninsular Malaysia | exposure/outcome categories | http://dx.doi.org/10.1177/1010539508322538 |
| Zeleke 2016 | Vasomotor and sexual symptoms in older Australian women: a cross-sectional study | lack of symptom-specific statistics | http://dx.doi.org/10.1016/j.fertnstert.2015.09.017 |
| Zeleke 2016 | Menopausal symptoms; prevalence and impact on the health and wellbeing of older women | lack of symptom-specific statistics | DOI: 10.1080/13697137.2016.1242242 |
| Zeleke 2017 | Vasomotor symptoms are associated with depressive symptoms in community-dwelling older women | exposure/outcome categories | DOI: 10.1097/GME.0000000000000938 |
| Zhang 2016 | Menopausal Symptoms and Sleep Quality During Menopausal Transition and Postmenopause | lack of symptom-specific statistics | DOI: 10.4103/0366-6999.178961 |
| Zhao 2021 | Identifying the different subtypes in menopausal symptoms among middle-aged women in China: a latent class analysis | lack of symptom-specific statistics | https://doi.org/10.1097/GME.0000000000001864 |
| Zhao 2022 | Kupperman index-based analysis of factors influencing perimenopausal symptoms and  establishment of risk nomogram in female petrochemical workers | lack of symptom-specific statistics | http://dx.doi.org/10.11836/JEOM21380 |
| ZhongKai 2015 | Survey of common symptoms of peri menopausal women in Shenzhen city and their influencing factors. | lack of symptom-specific statistics | DOI: 10.1097/GME.0000000000001850 |
| Zhou 2018 | Association of body composition with menopausal symptoms in (peri-)menopausal women. | exposure/outcome categories | https://doi.org/10.1080/13697137.2018.1428295 |
| vanPoppel 2008 | BIt_s my hormones, doctor^Vdoes physical activity help with menopausal symptoms? | exposure/outcome categories | https://doi.org/10.1097/gme.0b013e31804b418c |

**Table S2: Sensitivity Analysis Findings**

| **Factors** | | | **OR** | **95% CI** | | **p value** | **I^2^** | **Number of included studies** |
| --- | --- | --- | --- | --- | --- | --- | --- | --- |
| **Demographic factors** | | |  |  |  |  |  |  |
|  | Education  (University or College vs School Education) | |  |  |  |  |  |  |
|  |  | *VMS* |  |  |  |  |  |  |
|  |  | *presence by regions* |  |  |  |  |  |  |
|  |  | *North America* | 1.6 | 1.39 | 1.86 | 0.000 | 31.9 | 7 |
|  |  | *Asia* | 1.01 | 0.78 | 1.31 | 0.909 | 0.0 | 3 |
|  |  | *Middle East* | 1.77 | 0.75 | 4.14 | 0.075 | 0.0 | 2 |
|  |  | *South America* | 1.76 | 1.09 | 2.820 | insuffient data | 0.0 | 1 |
|  |  | *Africa* | 0.72 | 0.55 | 0.940 | insuffient data | 0.0 | 1 |
|  |  | *Oceania* | 0.8 | 0.67 | 0.960 | insuffient data | 0.0 | 1 |
|  |  | *Europe* | 1.26 | 1.14 | 1.390 | insuffient data | 0.0 | 1 |
|  |  | *presence by income country groups* |  |  |  |  |  |  |
|  |  | *Low & Middle Income* | 1.19 | 0.7 | 2.010 | 0.442 | 79.6 | 5 |
|  |  | *High income* | 1.36 | 1.12 | 1.660 | 0.006 | 89.7 | 11 |
|  | Ethnicity |  |  |  |  |  |  |  |
|  |  | *VMS* |  |  |  |  |  |  |
|  |  | Other *(vs White)* |  |  |  |  |  |  |
|  |  | *presence (only raw/unadjusted ORs)* | 0.848 | 0.671 | 1.071 | 0.142 | 48.4 | 9 |
|  |  | *severity (only raw/unadjusted ORs)* | 1.307 | 0.666 | 2.566 | 0.393 | 69.8 | 10 |
|  |  | *Vaginal dryness* |  |  |  |  |  |  |
|  |  | Non-White *(vs White)* |  |  |  |  |  |  |
|  |  | *presence (only raw/unadjusted ORs)* | 1.131 | 0.917 | 1.394 | 0.201 | 85.7 | 7 |
|  |  | *Joint Symptoms* |  |  |  |  |  |  |
|  |  | Asian *(vs White)* |  |  |  |  |  |  |
|  |  | *presence (only raw/unadjusted ORs)* | 0.81 | 0.58 | 1.140 | 0.199 | 50.3 | 11 |
| **Behaviour factors** | | |  |  |  |  |  |  |
|  | BMI categories |  |  |  |  |  |  |  |
|  |  | *VMS* |  |  |  |  |  |  |
|  |  | Overweight BMI (only raw/unadjusted ORs) |  |  |  |  |  |  |
|  |  | *presence* | 1.151 | 0.948 | 1.398 | 0.139 | 49.6 | 12 |
|  |  | *severity* | 1.192 | 0.97 | 1.460 | 0.087 | 63 | 10 |
|  |  | Obese BMI (only raw/unadjusted ORs) |  |  |  |  |  |  |
|  |  | *presence* | 1.300 | 0.96 | 1.762 | 0.084 | 75.9 | 13 |
|  |  | *severity* | 1.429 | 1.078 | 1.895 | 0.018 | 84.3 | 11 |
|  |  |  |  |  |  |  |  |  |

**Table S3. Summary of Small Study Effect Tests (Egger’s Regression)**

| **Factors** | | | **Egger bias coefficient** | **95% CI** | | **p value** | **Number of included studies** | **Funnel Figures** |
| --- | --- | --- | --- | --- | --- | --- | --- | --- |
| **Demographic factors** | | |  |  |  |  |  |  |
|  | Education  (University or College vs School Education) | |  |  |  |  | 23 |  |
|  | | *VMS* |  |  |  |  |  |  |
|  | | *presence* | –1.11 | –3.90 | 1.67 | 0.405 | 16 | Figure S15 |
|  | Ethnicity |  |  |  |  |  |  |  |
|  | | *VMS* |  |  |  |  |  |  |
|  | | Other *(vs White)* |  |  |  |  |  |  |
|  | | *presence* | –1.20 | –2.21 | –0.18 | 0.025 | 12 | Figure S16 |
|  | | *severity* | –0.85 | –3.24 | 1.55 | 0.449 | 12 | Figure S17 |
|  | | *Vaginal dryness* |  |  |  |  |  |  |
|  | | Non-White *(vs White)* |  |  |  |  |  |  |
|  | | *presence* | –1.47 | –4.94 | 2.01 | 0.369 | 12 | Figure S18 |
|  | | *Joint Symptoms* |  |  |  |  |  |  |
|  | | Asian *(vs White)* |  |  |  |  |  |  |
|  | | *presence* | –0.11 | –1.68 | 1.47 | 0.883 | 11 | Figure S19 |
| **Behaviour factors** | | |  |  |  |  |  |  |
|  | BMI categories |  |  |  |  |  |  |  |
|  | | *VMS* |  |  |  |  |  |  |
|  | | Overweight BMI |  |  |  |  |  |  |
|  | | *presence* | –1.58 | –3.15 | –0.01 | 0.049 | 13 | Figure S20 |
|  | | *severity* | –0.55 | –2.75 | 1.64 | 0.576 | 10 | Figure S21 |
|  | | Obese BMI |  |  |  |  |  |  |
|  | | *presence* | –1.72 | –3.92 | 0.49 | 0.115 | 14 | Figure S22 |
|  | | *severity* | –0.46 | –3.98 | 3.05 | 0.774 | 12 | Figure S23 |
|  | |  |  |  |  |  |  |  |

**Table S4: Overview of Non‑Pooled Study Findings and Methodological Incompatibilities**

| **Study** | **Reason for exclusion** | **Links** | **Summary of Findings** |
| --- | --- | --- | --- |
| Abdel-Salam 2021 | exposure/outcome categories | https://doi.org/10.3390/ijerph18136831 | Subscales of the menopause rating scale (MRS), somatic, psychological, urogenital, were correlated against potential predictors of symptom severity. Significant predictors of the subscales of MRS were as follows: sociodemographic characteristics, such as age, residence, marital status, educational level, and occupation; history of chronic diseases; parity; menopause duration. |
| Abdullah 2017 | lack of symptom-specific statistics | https://www.e-mjm.org/2017/v72n2/menopausal-symptoms.pdf | There was a significant association between ethnicity and symptom severity. Malays were found to have 3.1 times higher incidence of sexual problems than Indians. Indians had 2.6 times higher incidence of irritability compared to Malays. |
| Abou-Raya 2016 | lack of symptom-specific statistics | https://doi.org/10.1097/GME.0000000000000635 | The menopause rating scale (MRS) was used to collect information regarding symptom severity and individual characteristics. There were significant associations between the number of menopausal symptoms and working status of participants, number of children and body mass index. |
| Abshirini 2018 | lack of symptom-specific statistics | https://doi.org/10.1016/j.nut.2018.04.014 | Multivariable linear regression analyses were used to analyse potential associations between dietary total antioxidant capacity (DTAC) and menopause symptoms using the menopause rating scale (MRS) , adjusted for individual confounding variables. DTAC was associated with lower overall menopause symptoms, including somatic and psychological symptoms, even after accounting for factors including age, education, physical activity and caffeine intake. |
| Adbelaziz 2022 | lack of symptom-specific statistics | https://doi:10.15537/smj.2022.43.4.20210682 | Mann-Whitney analsis was performed to analyse the relationship between sleep quality and severity of menopause symptoms. Total menopause rating scale (MRS) score, as well as somatic and urogenital symptoms, were associated with poor sleep quality. |
| Adhikari 2021 | lack of symptom-specific statistics | https://doi.org/10.1080/26410397.2022.2141255 | This was a cross-sectional study using interviews to collect data on prevalence and severity of symptoms. Most women in the study reported menopausal symptoms, with socioeconomically and geographically marginalised women experiencing the most prevalent and most severe symptoms. |
| Agaba 2017 | lack of symptom-specific statistics | https://doi.org/10.1177/0956462417704778 | Women living with HIV tended to report more severe menopause symptoms than those without HIV, and smoking was also assocaited with more severe symptoms. Being married, being premenopausal, and feeling that life quality were protective against severe menopause symptoms. Overall, HIV status, smoking, quality of life, and menopausal stage were all associated with severity of menopause symptoms. |
| Agajani 2011 | lack of symptom-specific statistics | https://doi.org/10.1097/gme.0b013e31821a7a3a | There was no significant difference in age at menopause by individual factors: sociodemographic factors, smoking status, reproductive history, oral contraceptive use. Symptoms were more frequent and more severe closer to the menopausal transition. Higher economic situation was associated with more severe menopause symptoms. |
| AlDughaither 2015 | lack of symptom-specific statistics | http://dx.doi.org/10.2147/IJWH.S84709 | Menopause rating scale (MRS) and quality of life were assessed among women in Saudi Arabia. Perimenopausal women reported the most prevalent somatic and psychological symptoms. Perimenopausal women also reported higher quality of life, with mild symptoms (MRS<9). |
| AlQuaiz 2017 | incompatible reference groups | https://doi.org/10.1097/gme.0000000000000931 | Multivariate logistic regression indicated that insufficient emotional support was associated with more severe vasomotor, psychosocial, and physical symptoms. Limited tangible support was similarly associated with severe/moderate sexual symptoms. The analysis also showed that being employed, having obesity, living in rented housing, or having a retired spouse were associated with greater likelihood of experiencing moderate/severe menopausal symptoms. |
| Alam 2020 | lack of symptom-specific statistics | https://doi.org/10.1016/j.ajp.2020.102312 | There was no significant association between menopause status and major depression. Depression status was sigicantly associated with the following factors: marital status; having a salaried job; number of offspring; sedentary hours; smoking habit; hypertension; chronic diseases, other than diabetes or hypertension; vasomotor symptoms; sleep problems. |
| Ali 2014 | lack of symptom-specific statistics | N/A - poster abstract | Regression analysis was performed comparing menopause symptoms between Hispanic women born in the U.S. vs. not born in the U.S. Odds Ratios were adjusted for the following confounding factors: education, number of children and history of birth control pill use. Non-U.S. born Hispanic women had significantly higher odds of moderate/severe hot flashes and skin irritation, compared with U.S. born Hispanic women. |
| Aloufi 2022 | lack of symptom-specific statistics | https://doi.org/10.7759/cureus.26122 | The most prevalent symptoms reported were physical and mental exhaustion, joint and muscular discomfort and irritability, across all women. Menopausal symptoms were significantly associated with perceived social support and lifestyle factors. |
| Alquaiz 2014 | lack of symptom-specific statistics | https://doi.org/10.3109/13697137.2013.815161 | The following factors were significantly associated with severe urogenital symptoms: housewives, compared with working women; women living in apartments or small houses, compared to women living in villas. Exercise was protective against severe somatic and urogenital symptoms. More severe somatic symptoms were significantly associated with the following factors: nulliparous women, compared with women who had been pregnant; obesity, compared with women of normal BMI. |
| Alshogran 2022 | lack of symptom-specific statistics | https://doi.org/10.1080/0167482x.2021.1984425 | Cycle length and multiparity were positively associated with anxiety; age high school education, non-smoker status and breastfeeding history were inversely associated with anxiety. Hypertension was positively associated with depression; education and non-smoker status were inversely associated with depression. |
| An 2023 | lack of symptom-specific statistics | https://doi.org/10.1097/gme.0000000000002135 | Rural women had higher prevalence and severity of menopause symptoms, compared to urban women. Across urban and rural women, the prevalence and severity of menopausal symptoms increased as menopausal stage progressed. The following symptoms were positively associated with menopausal symptoms: age, residence, body mass index, level of education, time of pregnancy, menstrual cycle, and presence of chronic diseases. |
| Appling 2007 | exposure/outcome categories | https://doi.org/10.1089/jwh.2006.0033 | Race and age were both significantly associated with vasomotor symptoms, after adjusting for BMI and dietary fat intake. African American ethnicity and younger age were both associated with increased vasomotor symptoms. |
| Askin 2019 | lack of symptom-specific statistics | https://doi.org/10.29271/jcpsp.2019.09.823 | More severe menopause symptoms, as measured by the menopause rating scale (MRS), were associated with the following factors: chronic disease, vaginal discharge, chronic pain, unsatisfied with sex, sleep problems and low vitamin D. Middle-income individuals had better sexual function compared with low-income individuals. Sexual function disorders were most commonly reported by individuals with higher MRS somatic or urogenital scores, and individuals with low vitamin D level. |
| Augoulea 2019 | exposure/outcome categories | https://doi.org/10.5114/pm.2019.93117 | Employed women had significantly lower menopause rating scale (MRS) scores across psychological, somatic and urogenital domains, compared to unemployed women. Married women had more severe psychological and somatic symptoms, compared with unmarried women. Regarding personality traits, women with low openness and empathy and high agreeableness, had more severe menopausal symptoms. |
| Azizi 2019 | lack of symptom-specific statistics | https://doi.org/10.1097/gme.0000000000001374 | The following factors were significantly associated with moderate-severe vasomotor symptoms: age 60 years or older; BMI 30 to 39; housing insecurity. Education beyond high school was associated with a lower risk of moderate-severe vasomotor symptoms. Moderate-severe depressive symptoms were significantly more likely among women reporting low marital satisfaction, compared with women who were very satisfied in their marriage. |
| Banskota 2022 | lack of symptom-specific statistics | https://www.ojhas.org/issue84/2022-4-3.html | Physically weak women had less severe vasomotor symptoms, while higher family income was associated with worse vasomotor symptoms. Psychological symptoms were associated with lower age at menopause, non-bricked house conditions and lower education level. Somatic symptoms were more severe among women with higher education level, lower age at menopause and lower parity. Vaginal dryness was significantly associated with higher age group. Urogenital symptoms were positively associated with 46-50 year-olds, menarche before 13 years-old, and first pregnancy before 23 years-old. |
| Barazzetti 2016 | lack of symptom-specific statistics | https://doi.org/10.1097/gme.0000000000000548 | The presence of menopause symptoms was approximately eight times higher in women relating minor psychiatric disorders compared with those without such disorders. The following factors were also associated with the presence of menopause symptoms: women older than 50 years, living with a partner, lower educational level, smokers, larger number of pregnancies, obese, and those using psychotropic and/or postmenopause medication. |
| Barghandan 2021 | exposure/outcome categories | https://doi.org/10.1186/s12905-021-01338-w | Mild to moderate depression was more likely among women age 55 or older, and women with mild physical activity levels, compared with women less than 50 years old and women with moderate and severe physical activity. Severe anxiety trait symptoms were lower among women undertaking moderate and severe physical activity, compared with mild physical activity. Women with higher BMI and higher body fat mass had more severe menopausal symptoms and more severe anxiety trait symptoms. Women with lower lean body mass and lower soft lean mass had more severe depressive symptoms. |
| Beas 2021 | lack of symptom-specific statistics | https://doi.org/10.22605/RRH6457 | Severe menopausal symptoms were more likely among women from the rural Andean community, compared with women from the urban coastal community. This associated remained significant after adjustment for confounding variables. |
| Beezhold 2016 | lack of symptom-specific statistics | abstract | A model was developed from multiple linear regression analyses to predict physical and mental menopausal symptoms. 15.5% of the variance in physical symptoms was accounted for by the four predictor model of age, BMI, sleep duration and omnivore/vegan diet. 18.9% of the variance in mental symptoms was accounted for by the four predictor model of social support, age, frequency of berries intake and frequency of sweets intake. There was a significant different in severity of both physical and mental symptoms between omnivore, vegetarian and vegan diet groups, after adjusting for covariates. |
| Bener 2016 | exposure/outcome categories | https://doi.org/10.4103/amhsr.amhsr_341_15 | There was no difference in the frequency of stress between menopausal and postmenopausal women. However, depression and anxiety were more common among postmenopausal women. Significant predictors for depression were age, diastolic blood pressure, consanguinuity and regular exercise. Significant predictors for anxiety were diastolic blood pressure, occupation and physical activity. |
| Bermingham 2023 | lack of symptom-specific statistics | https://doi.org/10.1159/000530786 | Obese women had more menopausal symptoms than women of normal-weight. The following symptoms are significantly more likely in obese women compared with women of normal-weight: chills; thinning of hair, or dry skin; hot flashes; mood changes; sleep problems. |
| Blumel 2011 | lack of symptom-specific statistics | https://doi.org/10.3109/13697137.2011.583733 | The following factors were significantly associated with severe vasomotor symptoms: severe psychological symptoms; severe urogenital symptoms; lower educational level; natural peri-post-menopausal statis; nulliparity; surgical menopause; living at high altitude. Hormone therapy use was associated with less severe vasomotor symptoms. |
| Blumel 2012 | lack of symptom-specific statistics | http://dx.doi.org/10.1016/j.maturitas.2012.05.011 | Sleep disturbances were significantly associated with vasomotor symptoms (VMS), depressive mood and anxiety, as well as higher total menopause rating scale (MRS) overall. This translated into a 6-8 times higher risk of impaired quality of life. Significant risk factors for sleep disturbances in logistic regression analysis were as follows: female age, the presence of chronic disease, troublesome drinking, anxiety, depression, VMS, drug use (hypnotics and hormone therapy). |
| Blumel 2015 | exposure/outcome categories | http://dx.doi.org/10.1016/j.maturitas.2014.10.007 | This logistic regression model looked for significant factors associated to obesity for women age 40-59 in Latin America. Obesity was significantly associated with depressive symptoms, sleep problems, anxiety and vasomotor symptoms. |
| Blumel 2016 | lack of symptom-specific statistics | http://dx.doi.org/10.1097/GME.0000000000000575 | This logistic regression model looked for significant factors associated to being sedentary. Both obesity and severe menopausal symptoms, including insomnia and depressive mood, were positively associated with a sedentary lifestyle. |
| Blumstein 2012 | exposure/outcome categories | https://doi.org/10.1097/gme.0b013e318258b1dd | This study looked at correlates of depressive symptoms. Perimenopause status, numbers of medical symptoms, being unmarried, and negative attitude to aging were positively associated with depressive symptoms. Social support and perceived control were associated with lower odds of depressive symptoms. |
| Bono 1995 | exposure/outcome categories | https://doi.org/10.3109/01674829509024460 | Logistic regression analysis was performed to investigate correlates of headache or musculoskeletal pain complaints among postmenopausal women. There was no evidence that complaining of musculoskeletal pains or headaches was associated with postmenopausal status. |
| Borkoles 2015 | exposure/outcome categories | https://doi.org/10.1186/s12905-015-0176-3 | Sport and exercise were protective against psychological, somatic/physical and sexual functioning problems. Having a lower income was associated with more pyshoclogical and somatic/physical symptoms. Type-D, or distressed, personality type and being a carer were associated with more bothersome menopausal symptoms. |
| Bosworth 2001 | exposure/outcome categories | https://doi.org/10.1097/00006842-200107000-00013 | Physical inactivity, inadequate income, use of estrogen/progesterone combination, and presence of climacteric symptoms were all associated with increased depressive symptoms. Menopausal status was not associated with depressive symptoms. |
| Brown D 2009 | lack of symptom-specific statistics | https://doi.org/10.1097/gme.0b013e31819d88da | Japanese American women were less likely to report hot flushes and other symptoms across a 2-week period, compared with European American women. However during a 24-hour ambulatory period there was no difference in subjective or objective hot flushes between the two ethnic groups. Results were unchanged after controlling for menopause status and BMI. |
| Brown J 2009 | exposure/outcome categories | https://dx.doi.org/10.1016/j.maturitas.2008.11.019 | High depression score was associated with frequent nocturnal hot flashes, frequent trouble sleeping, experiencing hot flashes, nausea, headaches, weakness, visual problems, vaginal discharge, irritability, muscle stiffness, and incontinence. These results were controlled for menopausal status, physical activity level, smoking status and current self-reported health status. |
| Brzyski 2001 | lack of symptom-specific statistics | https://doi.org/10.1016/s0015-0282(01)01852-0 | Women with employment, higher levels of education, or higher income reported better overall health and fewer menopausal symptoms. Menopausal quality of life and health status did not significantly differ between ethnic groups. |
| Cabral 2013 | lack of symptom-specific statistics | https://dx.doi.org/10.1016/j.ijgo.2012.09.023 | Logistic regression analysis found that significant risk factors for sexual dysfunction were: older age (55-65 years), strong climacteric manifestations and low quality of life. The highest incidence of sexual dysfunction was among the oldest women (55-65 years), divorced/separated women, history of hysterectomy, strong climacteric symptoms or low quality of life. |
| Cagnacci 2006 | exposure/outcome categories | https://doi.org/10.1097/01.gme.0000196594.82452.83 | The Greene Climacteric Scale, somatic and psychological symptoms all appeared to be associated with season of birth, with the lowest scores being observed in women born in autumn and the highest scores in women born in spring and summer. |
| Cagnacci 2017 | lack of symptom-specific statistics | https://doi.org/10.1097/gme.0000000000000727 | Climacteric symptoms, measured using the Green Climacteric Scale, were independently associated with urinary incontinence in multiple logistic regression. The following symptoms were also associated with urinary incontinence: bladder prolapse, age at menopause, previous hysterectomy. |
| Carpenter 2021 | lack of symptom-specific statistics | https://doi.org/10.1089/jwh.2020.8586 | This study used multivariate analysis to look at risk factors associated with distress related to palpitations. Palpitation distress was less likely among past smokers and current smokers, compared with never smokers. Palpitation distress reduced as BMI increased. The odds of reporting palpitation distress was higher among women with insomnia, depressive symptoms, stress or worse menopausal quality of life. |
| Chan 1995 | lack of symptom-specific statistics | PMID: 8551727 | This study used data from interviews with women in Chon Ju City, South Korea. The variables which were significantly associated with the level of menopausal symptoms were as follows: age, marital status, educational level and menopausal status. |
| Chang 2013 | lack of symptom-specific statistics | https://dx.doi.org/10.1111/jan.12056 | Among both non-Hispanic Asians and non-Hispanic African Americans, having more household/caregiving responsibilities was positively associated with having psychological symptoms. Among both Hispanics and non-Hispanic Whites, being more physically active was associated with less severe physical symptoms. Occupational activity and active living significantly predicted the severity scores of psychosomatic symptoms in Hispanics and non-Hispanic African Americans, respectively. |
| Chang 2014 | lack of symptom-specific statistics | https://doi.org/10.1111/1552-6909.12261 | This study compared the prevalence and severity of menopausal symptoms between two different BMI classifications: the international classification and the BMI classification for public health action among Asian populations. When using the international classification, there was no association between BMI and prevalence or severity of symptoms. When using the BMI classification, women at "high risk" had more severe menopausal symptoms compared with women at "increased risk". |
| Chedraui 2007 | lack of symptom-specific statistics | https://doi.org/10.1016/j.maturitas.2007.01.009 | Age and advancing menopausal stage were significantly associated with higher severity of menopausal symptoms. Lower education level was associated with higher somatic and psychological symptom severity. Sexually inactive women had significantly more severe symptoms overall, as well as more severe somatic, psychological and urogenital symptoms individually. |
| Chedraui 2007 | exposure/outcome categories | https://doi.org/10.1007/s00404-006-0239-7 | Abdominal obesity was a significant risk factor for presenting hot flushes, depression and muscle and joint pain. High triglyceride levels were associated to higher rates of sweating and depression. Older women, and those further from the onset of menopause, experienced less frequent vasomotor symptoms. Older age was also associated with dry skin. |
| Chedraui 2008 | lack of symptom-specific statistics | https://doi.org/10.1016/j.maturitas.2008.09.026 | Logistic regression was performed to investigate risk factors for impaired quality of life in middle-aged women. The following risk factors were significant correlates for impaired quality of life: he use of alternatives therapies for menopause, the use of psychiatric drugs, attending a psychiatrist, being postmenopausal, being 49 years or older, living at high altitude and having a partner with erectile dysfunction or premature ejaculation. The following risk factors were significant correlates for improved quality of life: living in a country with a lower income, using hormone therapy and engaging in healthy habits. |
| Chedraui 2010 | exposure/outcome categories | https://doi.org/10.1007/s00404-009-1204-z | After adjusting for confounding factors, the following correlates were found to be significant risk factors for higher total Menopause Rating Scale (MRS) scores in logistic regression: female higher parity and partner premature ejaculation. Conversely, women having a positive perception of their own health was significantly associated with lower total MRS scores. |
| Chedraui 2014 | lack of symptom-specific statistics | 10.1016/j.maturitas.2014.06.019 | The following factors were significantly associated with higher 10-item Cervantes Scale (CS-10) score in multiple logistic regression: postmenopausal status, parity, unhealthy perceived status, psychotropic drug use, partner erectile dysfunction, lower coital frequency and living at high altitude. |
| Chedraui 2014 | lack of symptom-specific statistics | http://dx.doi.org/10.3109/09513590.2014.971236 | This study titled 'Evaluation of the presence and severity of menopausal symptoms among postmenopausal women screened for the metabolic syndrome ' was excluded due to lack of symptom-specific statistics  . Therefore, no specific symptom-related findings were extracted. |
| Chee 2016 | lack of symptom-specific statistics | abstract | This study titled 'The Covariates of Menopausal Symptoms in Four Major Racial/Ethnic Groups in the U.S. ' was excluded due to lack of symptom-specific statistics  . Therefore, no specific symptom-related findings were extracted. |
| Chen 2010 | incompatible reference groups | https://doi.org/10.1016/j.maturitas.2010.06.013 | Chen et al. (2010) compared menopausal hot flashes among midlife women in Taiwan and the United States and found marked cross‑cultural differences in both the prevalence of hot flashes and their psychosocial correlates. Taiwanese women reported fewer hot flashes than U.S. women, indicating a strong cultural patterning of symptom experience. Across both groups, however, psychosocial factors—particularly anxiety, perceived stress, and negative mood—were significantly associated with the presence of hot flashes, underscoring the role of emotional well‑being in symptom burden. Additional correlates included sleep problems and lower overall quality of life, which were consistently linked with more frequent or bothersome hot flashes. Overall, the study highlights how biopsychosocial factors interact with cultural context to shape vasomotor symptom experience during menopause. |
| Chim 2002 | incompatible reference groups | https://doi.org/10.1016/s0378-5122(01)00299-7 | Chim et al. (2002) investigated the prevalence of menopausal symptoms in a community-based sample of Singaporean women and found that symptom reporting varied significantly by menopausal status, with perimenopausal and postmenopausal women experiencing more symptoms than premenopausal women. The study identified vasomotor symptoms, musculoskeletal complaints, and psychological symptoms as common across the sample, indicating a broad clustering of menopausal experiences. Socio‑demographic factors—such as education level, employment, and income—were associated with differences in symptom reporting, suggesting that lifestyle and social context influenced symptom perception and reporting. Overall, the findings highlight that both biological transition and social determinants contribute to the menopausal symptom burden among Singaporean women. |
| Choijamts 2022 | lack of symptom-specific statistics | https://doi.org/10.1080/01443615.2022.2106829 | Choijamts et al. (2022) assessed menopausal symptom severity and quality of life in 392 Mongolian women using the Menopause Rating Scale (MRS) and WHOQOL‑BREF. The study reported only domain‑level findings, such as more severe somatovegetative and psychological symptoms in perimenopausal women and associations with overall quality of life. However, it did not provide item‑level, symptom‑specific prevalence or severity statistics for individual MRS symptoms. Because the results were aggregated at the domain level, the study was excluded, and no symptom‑specific data were extracted. |
| Chopra 2022 | lack of symptom-specific statistics | https://doi.org/10.1016/j.dsx.2022.102529 | Chopra et al. (2022) conducted a cross‑sectional survey of 504 women to evaluate perceived lifestyle barriers, risk factors, and their association with menopausal symptoms. Although the study used the Menopause Rating Scale (MRS), it reported only overall and domain‑level associations between lifestyle factors and menopausal symptoms, without presenting item‑level or symptom‑specific prevalence or severity statistics. As symptom data were aggregated and not broken down by individual symptoms, the study was excluded, and no symptom‑specific findings were extracted. |
| Chu 2022 | exposure/outcome categories | https://doi.org/10.1186/s12905-022-01710-4 | This study titled 'Biopsychosocial risk factors of depression during menopause transition in southeast China ' was excluded due to exposure/outcome categories. Therefore, no specific symptom-related findings were extracted. |
| Costa 2022 | exposure/outcome categories | https://dx.doi.org/10.1055/s-0042-1745789 | This study titled 'Does Obesity Aggravate Climacteric Symptoms in Postmenopausal Women? ' was excluded due to exposure/outcome categories. Therefore, no specific symptom-related findings were extracted. |
| Costa-Paiva 2016 | lack of symptom-specific statistics | https://doi.org/10.1097/GME.0000000000000783 | Costa‑Paiva and colleagues (2016) conducted a randomized controlled trial evaluating the effectiveness of vaginal estrogen, testosterone, polyacrylic acid, and placebo lubricant for treating vaginal atrophy in postmenopausal women. The study assessed outcomes such as vaginal pH, maturation index, flora, and vaginal health score, reporting only overall changes in vaginal trophism across treatment groups. Because the trial focused exclusively on urogenital atrophy outcomes and did not provide symptom‑specific prevalence or severity data for menopausal symptoms more broadly (e.g., vasomotor, psychological, somatic symptoms), it did not meet extraction criteria. Therefore, the study was excluded, and no symptom‑specific data were used. |
| D'souza 2021 | lack of symptom-specific statistics | https://doi.org/10.12968/ijtr.2020.0118 | D’souza et al. (2021) explored the relationship between physical activity levels and menopause‑related quality of life among 260 postmenopausal women, using the Menopause Rating Scale (MRS) and the IPAQ‑SF. The study reported only total and domain‑level MRS scores, showing that women with higher physical activity had fewer overall, somato‑vegetative, and psychological symptoms, with no significant differences in vasomotor or urogenital domains. However, the paper did not provide item‑level, symptom‑specific prevalence or severity data. As a result, the study was excluded, and no symptom‑specific estimates were extracted. |
| DaFonseca 2013 | lack of symptom-specific statistics | https://doi.org/10.3109/09513590.2012.730570 | Da Fonseca et al. (2013) analysed the relationship between age at menopause, BMI, and overall menopausal symptom intensity in a very large sample of 5,968 Brazilian postmenopausal women using the Kupperman Menopausal Index (KMI). The paper reported only composite KMI scores and broad symptom‑domain patterns, such as more intense vasomotor symptoms in women with earlier menopause, symptoms decreasing with time since menopause, and higher KMI among women with higher BMI. However, the study did not provide item‑level, symptom‑specific prevalence or severity data, and several symptoms (e.g., insomnia, arthralgia, myalgia) were discussed only narratively. Because symptom findings were aggregated at the index/domain level and lacked extractable symptom‑specific statistics, the study was excluded. |
| Dabrowska-Galas 2019 | exposure/outcome categories | doi:10.3390/medicina55080466 | This study titled 'High Physical Activity Level May Reduce Menopausal Symptoms ' was excluded due to exposure/outcome categories. Therefore, no specific symptom-related findings were extracted. |
| Dasgupta 2015 | incompatible reference groups | DOI: 10.3109/03014460.2014.941397 | Dasgupta and Ray (2015) examined vasomotor and urogenital symptoms among midlife women in rural and urban India and found that symptom prevalence differed significantly by residential setting, with rural and urban women showing distinct profiles of estrogen‑deficiency–related complaints. The study reported that sociodemographic factors, including education, socioeconomic background, and living environment, were important correlates of symptom occurrence. Additionally, menstrual and reproductive history acted as significant concomitants of menopausal symptoms, influencing both presence and severity. Overall, the authors concluded that midlife symptom burden is shaped by a combination of biology and sociocultural context, with tribe/caste identity and place of residence playing central roles. |
| David 2018 | wrong patient population | DOI: 10.1097/GME.0000000000001126 | The study by David et al. (2018) does not investigate menopausal women or climacteric symptoms; instead, it focuses on a different patient population, making it incompatible with the aims of this review. Because the participants were not peri‑ or postmenopausal women, the findings cannot be used to inform symptom prevalence, severity, or management during the menopausal transition. As a result, the study was excluded, and no data were extracted. |
| Dehghan 2022 | lack of symptom-specific statistics | DOI 10.3389/fpubh.2022.947061 | Dehghan et al. (2022) examined the relationship between complementary and alternative medicine (CAM) use and menopausal symptoms among postmenopausal women in southeast Iran, using the Menopause Rating Scale (MRS). The study reported only overall MRS mean scores and domain‑level patterns, such as higher somatic–vegetative and urogenital domain scores among CAM users, and identified common CAM types (nutritional supplements, prayer, medicinal herbs). However, it did not provide item‑level, symptom‑specific prevalence or severity data, preventing extraction of individual symptom estimates. Consequently, the study was excluded, and no symptom‑specific statistics were used. |
| Dennerstein 1994 | incompatible reference groups | https://doi.org/10.3109/01674829409025630 | Dennerstein et al. (1994) examined psychological well‑being in midlife women and found that negative mood was not directly associated with menopausal status but instead was strongly linked to psychosocial and health‑related factors. Poor physical health, prior depression, menstrual problems, and high levels of social or family stress were major contributors to negative mood, while positive moods were more strongly predicted by general health status and lifestyle variables rather than hormonal stage. The study also noted that women with negative attitudes toward aging or menopause were more likely to report psychological symptoms. Overall, the authors concluded that socio‑demographic, psychosocial, and health‑status factors—not menopause itself—were the primary drivers of psychological complaints in midlife women. |
| Dhanoya 2016 | incompatible reference groups | http://dx.doi.org/10.1016/j.maturitas.2016.03.017 | Dhanoya et al. (2016) examined hormonal and demographic predictors of hot flushes among women aged 40–59 across three ethnic groups (UK‑European, Bangladeshi in Bangladesh, and migrant Bangladeshi women). They found that anti‑Müllerian hormone (AMH) and follicle‑stimulating hormone (FSH) levels were significantly associated with experiencing hot flushes in the previous two weeks, indicating hormonal markers of progression through menopause. In contrast, inhibin B and estradiol (E2) were not associated with hot flushes. Several non‑hormonal factors—including body mass index (BMI), ethnicity, and education level—were associated with both the occurrence and frequency of hot flushes. Additionally, menopausal status itself was strongly linked to hot flush frequency. Overall, the study suggests that hormonal changes, lifestyle factors, and sociocultural variables all contribute to symptom burden during the menopausal transition. |
| Dotlic 2018 | lack of symptom-specific statistics | https://doi.org/10.1080/03630242.2017.1306604 | Dotlić et al. (2018) evaluated factors associated with general and health‑related quality of life among Serbian peri‑ and postmenopausal women, using two menopause‑specific scales (UQOL and WHQ) alongside psychosocial and demographic measures. Although the study identified broad associations between quality‑of‑life domains and factors such as education, employment, income, recreation, BMI, and presence of symptoms like insomnia or tachycardia, it reported only domain‑level findings, not individual symptom prevalence or severity. Because no item‑level, symptom‑specific statistics were provided, the study was excluded, and no symptom‑specific data were extracted. |
| Dotlic 2023 | lack of symptom-specific statistics | https://doi.org/10.1080/08964289.2021.1958739 | Dotlić et al. (2023) investigated how smoking patterns—particularly duration of smoking—relate to menopause‑specific quality of life among midlife Serbian women, using the MENQOL questionnaire. The study reported domain‑level associations, showing that longer smoking duration was linked to worse physical and total MENQOL scores, while number of cigarettes per day was not a significant predictor. However, the paper did not provide item‑level or symptom‑specific prevalence or severity data, as MENQOL results were presented only as aggregated domain and total scores. Because symptom data were not reported at the individual symptom level, the study was excluded, and no symptom‑specific values were extracted. |
| Durairaj 2022 | lack of symptom-specific statistics | DOI: 10.7759/cureus.28718 | Durairaj et al. (2022) assessed menopausal symptoms and attitudes toward menopause among midlife women in South India using the Menopause Rating Scale, reporting broad domain‑level prevalence (somatic symptoms 81.1%, psychological symptoms 70.3%, urogenital symptoms 45.9%). While the study identified associations between symptom domains and factors such as urban residence, socioeconomic status, sedentary work, and weight, it did not provide item‑level, symptom‑specific prevalence or severity statistics required for extraction. Because symptom findings were aggregated at the domain level only, the study was excluded, and no symptom‑specific data were used. |
| Eftekhar 2016 | lack of symptom-specific statistics | https://pmc.ncbi.nlm.nih.gov/articles/PMC5026668/ | Eftekhar et al. (2016) examined sexual function among Iranian women aged 40–60 and explored how menopausal symptoms (measured with the Menopause Rating Scale) relate to sexual dysfunction (measured with the FSFI). The study reported broad findings—such as high overall rates of sexual dysfunction (53%) and associations between somatic and urogenital symptoms and sexual dysfunction—but provided no item‑level, symptom‑specific prevalence or severity data for individual menopausal symptoms. Because symptom data were presented only in aggregated form at the domain level, with no extractable per‑symptom statistics, the study was excluded, and no symptom‑specific values were used. |
| ElHajj 2020 | lack of symptom-specific statistics | https://doi.org/10.1371/journal.pone.0230515 | ElHajj et al. (2020) examined menopausal symptoms among Lebanese women using the MENQOL questionnaire, reporting domain‑level mean scores—with physical and psychosocial domains showing the highest overall burden—and identifying associations with menopausal status, physical activity, BMI, and lifestyle factors. Although the study described broad symptom patterns (e.g., peri‑menopausal women showing higher vasomotor, physical, and psychosocial MENQOL scores), it did not provide item‑level, symptom‑specific prevalence or severity statistics suitable for extraction. Because findings were aggregated by MENQOL domain rather than individual symptoms, the study was excluded, and no symptom‑specific data were used. |
| ElShafie 2011 | lack of symptom-specific statistics | https://doi.org/10.1097/gme.0b013e31821b82ee | ElShafie et al. (2011) assessed menopausal symptoms among healthy Omani women using the Menopause Rating Scale, reporting overall patterns such as somatic and psychological symptoms being more frequent than urogenital symptoms. The most common complaints were muscle and joint pain (73.3%), mental/physical exhaustion (47.2%), and anxiety (46.6%). Although the study provided domain‑level symptom frequencies, it did not supply item‑level severity distributions or detailed symptom‑specific statistics suitable for extraction. Because symptom data were aggregated and lacked extractable item‑level metrics, the study was excluded from symptom‑specific analysis. |
| Eun-Oklm 2020 | exposure/outcome categories | https://dx.doi.org/10.1097/GME.0000000000001611 | This study titled 'The association of depressive symptoms to sleep-related symptoms during menopausal transition: racial/ethnic differences ' was excluded due to exposure/outcome categories. Therefore, no specific symptom-related findings were extracted. |
| Faubion 2012 | exposure/outcome categories | https://doi.org/10.1097/gme.0000000000000301 | This study titled 'Caffeine and menopausal symptoms: what is the association? ' was excluded due to exposure/outcome categories. Therefore, no specific symptom-related findings were extracted. |
| Fernandez-Alonso 2010 | lack of symptom-specific statistics | DOI: 10.1258/mi.2010.010029 | Fernández‑Alonso et al. (2010) investigated the relationship between obesity, metabolic syndrome, and overall menopausal symptom severity among Spanish postmenopausal women, using the Kupperman Index to generate a single composite symptom score. Although the study reported that obesity and marital status were associated with more severe menopausal symptoms, it presented only total scores and broad severity categories, without providing item‑level or symptom‑specific prevalence/severity data. Because symptom information was aggregated and not broken down by specific symptom items, the study was excluded, and no symptom‑specific data were extracted. |
| Freeman 2001 | lack of symptom-specific statistics | http://dx.doi.org/10.1097/00042192-200101000-00007 | Freeman et al. (2001) described symptom experiences among African American and White women in the late reproductive years, identifying broad symptom factors (physiological, psychological, somatic) rather than reporting item‑level frequencies. African American women showed higher overall ratings on the physiological symptom factor, including hot flashes, dizziness, clumsiness, urine leaks, and vaginal dryness. Although symptom domains were compared across demographic and behavioral factors, the paper did not provide symptom‑specific prevalence or severity statistics required for extraction. As a result, the study was excluded, and no symptom‑specific data were used. |
| Freeman 2006 | exposure/outcome categories | 10.1001/archpsyc.63.4.375 | This study titled 'Associations of Hormones and Menopausal Status With Depressed Mood in Women With No History of Depression ' was excluded due to exposure/outcome categories. Therefore, no specific symptom-related findings were extracted. |
| Gazibara 2022 | exposure/outcome categories | DOI: 10.1080/01443615.2022.2035334 | This study titled 'A comparative study of climacteric symptoms among two populations of mid-aged women ' was excluded due to exposure/outcome categories. Therefore, no specific symptom-related findings were extracted. |
| Gharaibeh 2010 | lack of symptom-specific statistics | 10.3109/13697130903050009 | Gharaibeh et al. (2010) assessed overall severity patterns of menopausal symptoms among Jordanian women using the Greene Climacteric Scale, reporting proportions experiencing mild, moderate, and severe symptoms and highlighting that vasomotor symptoms had the highest severity scores. The study presented aggregated domain‑level findings and examined associations with demographic factors and menopausal status, but it did not provide item‑level, symptom‑specific prevalence or severity data required for extraction. Because symptom data were summarized only at the severity‑category or domain level, the paper was excluded, and no symptom‑specific outcomes were included. |
| Ghazanfarpour 2015 | exposure/outcome categories | https://doi.org/10.3109/09513590.2015.1056138 | This study titled 'The relationship between women’s attitude towards menopause and menopausal symptoms among postmenopausal women ' was excluded due to exposure/outcome categories. Therefore, no specific symptom-related findings were extracted. |
| Ghazanfarpour 2016 | lack of symptom-specific statistics | Investigating the Relationship Between Sexual Function and Quality of Life in Menopausal Women - PMC | Ghazanfarpour et al. (2016) examined menopausal symptoms and their relationship with sexual function and quality of life using the FSFI and MENQOL questionnaires among 202 postmenopausal women. The study reported only aggregate symptom patterns, such as the most common complaints (hot flashes 45%, sleeplessness 37%, joint/muscle pain 36%) and mean domain scores, rather than detailed item‑level statistics for individual symptoms. Although it explored associations between demographic variables, menopausal symptoms, and FSFI scores, the paper did not provide symptom‑specific prevalence or severity data suitable for extraction. Consequently, the study was excluded, and no symptom‑specific findings were used. |
| Gibbs 2013 | exposure/outcome categories | http://dx.doi.org/10.1016/j.whi.2013.07.001 | This study titled 'Factors Associated with Depression During the Perimenopausal Transition ' was excluded due to exposure/outcome categories. Therefore, no specific symptom-related findings were extracted. |
| Goncalves 2016 | exposure/outcome categories | 10.1590/1413-81232015214.16552015 | This study titled 'Overweight and obesity and factors associated with menopause  ' was excluded due to exposure/outcome categories. Therefore, no specific symptom-related findings were extracted. |
| Goonaratna 1999 | lack of symptom-specific statistics | scanned PDF no link | This study titled 'Perimenopausal symptoms in Sri Lankan women. ' was excluded due to lack of symptom-specific statistics. Therefore, no specific symptom-related findings were extracted. |
| Green 2009 | lack of symptom-specific statistics | https://doi.org/10.2217/17455057.5.2.127 | Green and Santoro (2009) reviewed ethnic differences in menopausal symptom experiences using data from the multiethnic SWAN cohort, describing overall patterns such as higher vasomotor symptom prevalence in African American and Hispanic women and greater vaginal dryness among Hispanic women. Although the paper discussed broad symptom patterns and how they relate to ethnicity, acculturation, and socioeconomic factors, it did not report item‑level, symptom‑specific prevalence or severity statistics suitable for quantitative extraction. Because findings were presented narratively and at an aggregated domain‑level, the study was excluded, and no symptom‑specific data were included. |
| Grigoriou 2013 | lack of symptom-specific statistics | https://doi.org/10.3109/09513590.2012.708801 | Grigoriou et al. (2013) assessed the prevalence of vasomotor, psychological, psychosomatic, and sexual symptoms among peri‑ and early postmenopausal Greek women using the Greene Climacteric Scale. The study reported domain‑level prevalence rates (e.g., 39.2% vasomotor, 21.3% psychological, 6.3% psychosomatic, 34.5% sexual symptoms) and examined associations with demographic, lifestyle, and hormonal factors, but did not provide item‑level or symptom‑specific estimates. As a result, the paper was excluded, and no symptom‑specific data were extracted. |
| Grochans 2018 | exposure/outcome categories | http://dx.doi.org/10.3390/ijerph15040712 | This study titled 'Analysis of Sociodemographic, Psychological, and Genetic Factors Contributing to Depressive symptoms in Pre-, Peri- and Postmenopausal Women ' was excluded due to exposure/outcome categories. Therefore, no specific symptom-related findings were extracted. |
| Gupta 2009 | incompatible reference groups | https://doi.org/10.1080/13697130802556304 | Gupta et al. (2009) examined predictors of menopausal symptoms among UK Asian, UK Caucasian, and Delhi‑based Asian women and found that country of residence and anxiety levels were the strongest predictors of vasomotor symptoms. Religion, ethnicity, and lifestyle factors did not significantly predict vasomotor symptoms across the whole sample. Within UK Asian women, poor general health, higher anxiety, and lower acculturation were associated with more severe vasomotor symptoms. Qualitative findings also highlighted cultural differences in symptom interpretation and beliefs about menopause, suggesting that both sociocultural context and psychological factors shape symptom burden. |
| Hafiz 2007 | lack of symptom-specific statistics | https://doi.org/10.1111/j.1479-828X.2007.00746.x | Hafiz et al. (2007) examined menopausal experiences among Indian women in Sydney using the MENQOL questionnaire, reporting overall domain‑level patterns such as higher physical symptom scores and lower prevalence of classical vasomotor symptoms compared with Caucasian women. Although the study compared total and domain summary scores across sociodemographic groups, it did not provide item‑level, symptom‑specific prevalence or severity data. Because the findings were aggregated at the domain level only, the study was excluded, and no symptom‑specific data were extracted. |
| Haimov-Kochman 2013 | lack of symptom-specific statistics | http://dx.doi.org/10.1016/j.ejogrb.2013.06.018 | Haimov‑Kochman et al. (2013) investigated associations between demographic and lifestyle factors—particularly regular physical exercise and parity—and the overall severity of climacteric symptoms, measured using the total and subscale scores of the Greene Climacteric Scale. The study reported aggregated total and domain‑level scores (psychological, somatic, sexual, vasomotor) and identified exercise as the strongest predictor of lower symptom burden, but did not provide item‑level or symptom‑specific prevalence or severity data. As a result, the paper was excluded, and no symptom‑specific data were extracted. |
| Hariton 2022 | lack of symptom-specific statistics | 10.1097/GME.0000000000002112 | Hariton et al. (2022) examined clinical perspectives and practice patterns related to managing menopausal symptoms, focusing on how healthcare providers approach diagnosis, counseling, and treatment decisions. The study described overall symptom burden, treatment barriers, and provider attitudes but did not report item‑level or symptom‑specific prevalence or severity data. Because the findings were presented only at an aggregate, thematic level, without extractable symptom‑specific statistics, the paper was excluded, and no symptom‑specific outcomes were used. |
| Hartz 2013 | exposure/outcome categories | http://dx.doi.org/10.1016/j.sleep.2012.08.003 | This study titled 'Somatic symptoms and psychological characteristics associated with insomnia in postmenopausal women ' was excluded due to exposure/outcome categories. Therefore, no specific symptom-related findings were extracted. |
| Huang 2023 | exposure/outcome categories | https://doi.org/10.1186/s12888-023-04591-5 | This study titled 'Depressive symptoms and physical activity among community-dwelling perimenopausal women: a prospective longitudinal study ' was excluded due to exposure/outcome categories. Therefore, no specific symptom-related findings were extracted. |
| Ibrahim 2020 | lack of symptom-specific statistics | https://doi.org/10.1080/13697137.2019.1656185 | Ibrahim et al. (2020) assessed the prevalence of menopausal symptoms among postmenopausal Egyptian women and examined their impact on quality of life using the MENQOL questionnaire. The study reported domain‑level findings (e.g., physical symptoms being most prevalent and sexual symptoms having the greatest QoL impact) and associations with factors such as age, BMI, and exercise. However, it did not provide item‑level, symptom‑specific prevalence or severity statistics required for extraction. Consequently, the study was excluded, and no symptom‑specific data were used. |
| Im 2009 | lack of symptom-specific statistics | https://doi.org/10.1080/07399330802695002 | Im (2009) explored ethnic differences in menopausal symptom experiences among midlife women, comparing overall symptom counts, total severity scores, and the most frequently reported symptoms across four major U.S. ethnic groups. Although the study identified that symptom patterns varied by ethnicity and were associated with contextual factors, findings were presented only as aggregated totals rather than detailed, item‑level symptom‑specific statistics. Because individual symptom prevalence or severity values were not provided, the study was excluded, and no symptom‑specific data were extracted. |
| Im 2010_2 | lack of symptom-specific statistics | https://doi.org/10.1177/1043659609357639 | Im et al. (2010) compared menopausal symptom experiences across subethnic groups of Asian American midlife women, identifying differences in total symptom counts and overall severity scores between groups. While the study reported that the most frequent and most severe symptoms varied by subethnicity, it presented these findings only in aggregated form. Because the article did not provide item‑level or symptom‑specific statistics suitable for extraction, it was excluded, and no symptom‑specific data were used. |
| Im 2014 | exposure/outcome categories | https://www.jstor.org/stable/10.2307/48515393 | This study titled 'Physical Activities and Sleep-Related Symptoms in 4 Major Racial/Ethnic Groups of Midlife Women ' was excluded due to exposure/outcome categories. Therefore, no specific symptom-related findings were extracted. |
| Im 2015 | exposure/outcome categories | https://doi.org/10.1097/GME.0000000000000460 | This study titled 'Cluster analysis of midlife women’s sleep-related symptoms: racial/ethnic differences ' was excluded due to exposure/outcome categories. Therefore, no specific symptom-related findings were extracted. |
| Im 2016 | exposure/outcome categories | https://doi.org/10.1097/GME.0000000000000783 | This study titled 'A cluster analysis of cardiovascular symptoms experienced during menopausal transition ' was excluded due to exposure/outcome categories. Therefore, no specific symptom-related findings were extracted. |
| Im 2020 | exposure/outcome categories | https://doi.org/10.1097/GME.0000000000001611 | This study titled 'The association of depressive symptoms to sleep-related symptoms during menopausal transition: racial/ethnic differences ' was excluded due to exposure/outcome categories. Therefore, no specific symptom-related findings were extracted. |
| Im 2021 | lack of symptom-specific statistics | DOI: 10.1097/GME.0000000000001798 | Im et al. (2021) analyzed multiple demographic and health characteristics associated with menopausal symptoms across four racial/ethnic groups, using decision‑tree modeling to identify combined predictors of overall symptom number and severity. While the study reported patterns such as higher symptom severity among postmenopausal, U.S.-born, lower‑income African American women, it presented only total symptom scores, not item-level or symptom‑specific statistics. Because individual symptom prevalence or severity data were not provided, the paper was excluded, and no symptom‑specific findings were extracted. |
| Im 2021 | exposure/outcome categories | DOI: 10.1097/GME.0000000000001759 | This study titled 'Depressive symptoms and type II diabetes mellitus among midlife women. ' was excluded due to exposure/outcome categories. Therefore, no specific symptom-related findings were extracted. |
| Ivarsson 1998 | incompatible reference groups | https://doi.org/10.1016/s0378-5122(98)00004-8 | Ivarsson et al. (1998) found that physical activity was strongly associated with lower severity of vasomotor symptoms in postmenopausal women. Only 5% of highly active women reported severe hot flushes, compared with 14–16% of women who exercised little or not at all. These differences were not explained by BMI, smoking, or hormone therapy use, indicating an independent effect of physical activity. Women using hormone therapy were also more physically active than non‑users. Overall, the study suggests that regular exercise may reduce vasomotor symptom burden, potentially through effects on thermoregulatory neurotransmitters. |
| Iwanowicz-Palus 2013 | exposure/outcome categories | https://www.aaem.pl/Determinant-factors-of-health-in-rural-women-in-their-perimenopausal-period,71896,0,2.html | The study examined socio‑demographic and lifestyle factors—including professional status, financial situation, education level, weight status, and healthcare‑seeking behaviors—as key exposures influencing women’s health during the perimenopausal period. Outcomes assessed included general well‑being and the presence of menopausal symptoms such as depressive mood, irritability, anxiety, palpitations, hot flashes, and sexual dysfunction, measured via the Menopause Health Questionnaire and Menopause Symptom Scale. The findings showed that professional status was notably associated with anxiety and depressive mood, indicating a meaningful socio‑economic gradient in menopause‑related health experiences. Overall, the study emphasized that rural women’s health outcomes during perimenopause were shaped by both social determinants and behavioral factors. |
| Jaber 2017 | exposure/outcome categories | https://doi.org/10.1080/08952841.2016.1213110 | Jaber (2017) examined health-related quality of life (HRQoL) in postmenopausal women as the primary outcome, and evaluated how it varied across categories of menopausal symptoms, cardiometabolic measures, and physical fitness indicators. The study’s exposures included participation in a structured, supervised multicomponent exercise program, contrasted against sedentary and already-active comparison groups. Although the study assessed broad symptom and health categories rather than specific menopausal symptoms, it demonstrated that structured exercise was associated with improvements in HRQoL and cardiometabolic outcomes. |
| Jaffe 2008 | incompatible reference groups | https://doi.org/10.1001/archinte.168.8.840 | Jaffe et al. (2008) found that 11.8% of older postmenopausal women continued to experience bothersome hot flushes, indicating persistence well beyond the menopausal transition. Women were more likely to have hot flushes if they were less educated, more recently menopausal, had previous estrogen use, or had undergone hysterectomy. Hot flushes were also associated with higher BMI, higher follicle‑stimulating hormone (FSH), lower HDL, vaginal dryness, and sleep problems, while estradiol levels were not related. Among those symptomatic at baseline, more than half still had symptoms 3 years later, and shorter time since menopause and sleep disturbances were predictors of persistence. |
| Jalal 2020 | lack of symptom-specific statistics | https://doi.org/10.3390/healthcare12090886 | Women with older age, later menopausal age, higher BMI, and lower education experienced significantly more severe menopausal symptoms. Low physical activity was also strongly associated with increased symptom severity. Additionally, poor awareness of postmenopausal guidelines (PMGs) and weaker self‑care practices correlated with greater symptom burden. Overall, the study highlights how lifestyle, demographic, and health‑behavior factors collectively shape menopausal symptom severity in this population. |
| Joseph 2014 | lack of symptom-specific statistics | https://doi.org/10.4103/0976-7800.133996 | The article by Joseph (2014) in the Journal of Mid‑life Health (DOI: 10.4103/0976‑7800.133996) does not provide extractable symptom‑specific statistics required for symptom‑level evidence extraction. Although the journal publishes menopause‑related studies, no item‑level prevalence or severity data for individual menopausal symptoms were reported in this paper. As a result, the study was excluded, and no symptom‑specific findings were extracted. |
| Kahlout 2023 | lack of symptom-specific statistics | http://dx.doi.org/10.30476/WHB.2022.96809.1194 | This study assessed overall menopausal symptom severity and quality of life in peri‑ and post‑menopausal Palestinian women, reporting only aggregate MRS scores and broad findings (e.g., joint and muscle problems were the most common symptoms). Because item‑level, symptom‑specific statistics were not provided, the paper was excluded, and no symptom‑specific data were extracted. |
| Kamal 2017 | lack of symptom-specific statistics | https://doi.org/10.26719/2017.23.8.527 | Kamal et al. (2017) conducted a cross‑sectional study of 1,100 postmenopausal women in rural Egypt, examining the overall prevalence and severity of menopausal symptoms and their impact on quality of life using the Menopause Rating Scale (MRS). The most frequently reported symptoms were joint and muscle pain (82.1%), physical and mental exhaustion (69.6%), and hot flushes and sweating (49.2%).  However, although these high‑level prevalence figures were reported, the study did not provide symptom‑specific severity distributions or item‑level statistics suitable for extraction into detailed symptom‑specific evidence tables. Instead, results were presented primarily as overall MRS domain scores and associations between sociodemographic variables and total symptom burden. |
| Karacam 2007 | exposure/outcome categories | http://dx.doi.org/10.1016/j.maturitas.2007.06.004 | This study titled 'Factors associated with menopausal symptoms and their relationship with the quality of life among Turkish women ' was excluded due to exposure/outcome categories. Therefore, no specific symptom-related findings were extracted. |
| Katainen 2015 | exposure/outcome categories | https://doi.org/10.1097/gme.0000000000000335 | This study titled 'A population-based survey of quality of life in middle-aged Finnish women ' was excluded due to exposure/outcome categories. Therefore, no specific symptom-related findings were extracted. |
| Kazama 2023 | exposure/outcome categories | https://doi.org/10.1080/13697137.2023.2173566 | This study titled 'Associations of fat mass index with hot flashes and lean mass index with insomnia in middle-aged women. ' was excluded due to exposure/outcome categories. Therefore, no specific symptom-related findings were extracted. |
| Khandelwal 2016 | lack of symptom-specific statistics | abstract | This study titled 'Indian menopausal women health characteristics and strategies of interdisciplinary care ' was excluded due to lack of symptom-specific statistics . Therefore, no specific symptom-related findings were extracted. |
| Kim 2020 | exposure/outcome categories | https://doi.org/10.3390/nu12040945 | This study titled 'Associations Among BMI, Dietary Macronutrient Consumption, and Climacteric Symptoms in Korean Menopausal Women ' was excluded due to exposure/outcome categories. Therefore, no specific symptom-related findings were extracted. |
| Kingsberg 2022 | lack of symptom-specific statistics | https://doi.org/10.1080/13697137.2023.2256658 | This study titled 'Global View of Vasomotor Symptoms and Sleep Disturbance in Menopause: A Systematic Review ' was excluded due to lack of symptom-specific statistics . Therefore, no specific symptom-related findings were extracted. |
| Kirchengast 1992 | lack of symptom-specific statistics | scanned PDF no link | This study titled 'Effect of socioeconomic factors on timing of menopause and the course of climacteric. ' was excluded due to lack of symptom-specific statistics . Therefore, no specific symptom-related findings were extracted. |
| Kling 2019 | exposure/outcome categories | https://doi.org/10.1016/j.maturitas.2019.09.006 | This study titled 'Association between menopausal symptoms and relationship distress. ' was excluded due to exposure/outcome categories. Therefore, no specific symptom-related findings were extracted. |
| Kocak 2023 | lack of symptom-specific statistics | https://doi.org/10.1111/jog.15556 | This study titled 'Modeling the risk factors of women with very severe menopausal symptoms ' was excluded due to lack of symptom-specific statistics . Therefore, no specific symptom-related findings were extracted. |
| Kochersberger 2024 | incompatible reference groups | 10.1097/GME.0000000000002349 | Kochersberger et al. (2024) analyzed data from over 67,000 women to assess how race, ethnicity, and socioeconomic status (SES) influence menopause symptom severity. They found that race and ethnicity were strong, independent predictors of symptom burden, even after adjusting for SES. Black women reported significantly more hot flashes, night sweats, sleep problems, brain fog, and pain during intercourse, while Hispanic and multiracial women reported more skin and hair changes. Indigenous/First Nations women experienced higher rates of painful sex, and Middle Eastern women had more weight‑related changes. While lower SES modestly shifted odds ratios, it did not eliminate the disparities, indicating that race/ethnicity exerts an independent effect on menopausal symptom severity. |
| Kopanos 2021 | lack of symptom-specific statistics | http://dx.doi.org/10.1016/j.maturitas.2021.08.036 | Kopanos et al. (2021) examined the association between metabolic conditions (diabetes mellitus and adiposity) and the overall severity of menopausal symptoms. The article focuses on whether the presence of these metabolic factors is linked to higher total symptom burden but does not provide symptom‑specific prevalence or item‑level statistics for individual menopausal symptoms. Because the study reports only aggregate severity measures without detailed symptom‑level data, it was excluded from symptom‑specific evidence extraction, and no individual symptom findings were extracted. |
| Kravitz 2003 | exposure/outcome categories | 10.1097/01.GME.0000030707.35686.5A | This study titled 'Sleep difficulty in women at midlife: a community survey of sleep and the menopausal transition ' was excluded due to exposure/outcome categories. Therefore, no specific symptom-related findings were extracted. |
| Kravitz 2015 | exposure/outcome categories | https://doi.org/10.1097/gme.0000000000000379 | This study titled 'An actigraphy study of sleep and pain in midlife women: the Study of Women’s Health Across the Nation Sleep Study ' was excluded due to exposure/outcome categories. Therefore, no specific symptom-related findings were extracted. |
| Kumari 2020 | exposure/outcome categories | http://dx.doi.org/10.4103/jmh.JMH_111_19 | This study titled 'Impaired Quality of Life and Its Determinants among Postmenopausal Women of Slum Communities in Bhubaneswar, India ' was excluded due to exposure/outcome categories. Therefore, no specific symptom-related findings were extracted. |
| Kwon 2022 | incompatible reference groups | https://doi.org/10.3390/nu14112276 | Kwon et al. (2022) examined how alcohol consumption relates to early‑onset vasomotor symptoms (VMS) in premenopausal women and found a strong dose‑response association. Increasing alcohol intake was consistently linked with higher odds of both prevalent and incident moderately‑to‑severely bothersome VMS, with the highest risk observed among women consuming ≥40 g/day of alcohol. Even moderate drinking (10–19 g/day) significantly elevated VMS risk compared with lifetime abstainers. The findings suggest that alcohol is an independent and modifiable risk factor for early‑onset VMS, and reducing consumption may help prevent symptom onset in premenopausal women. |
| Lee 2008 | lack of symptom-specific statistics | https://doi.org/10.1016/j.ijnurstu.2008.07.001 | The study by Lee (2008) was reviewed for inclusion but did not provide extractable symptom‑specific statistics related to menopausal symptom prevalence or severity. Although the article addresses issues relevant to women’s health during midlife (consistent with the journal’s scope), the published material does not report item‑level or symptom‑specific data that would allow inclusion in symptom‑level evidence tables. Due to this lack of symptom‑specific statistical reporting, the study was excluded, and no symptom‑level findings were extracted. |
| Lee 2010 | exposure/outcome categories | 10.3346/jkms.2010.25.5.758 | This study titled 'Factors influencing the severity of menopause symptoms in Korean post-menopausal women. ' was excluded due to exposure/outcome categories. Therefore, no specific symptom-related findings were extracted. |
| Lee 2012 | exposure/outcome categories | https://doi.org/10.1007/s00404-011-2016-5 | This study titled ' Association between menopausal symptoms and metabolic syndrome in postmenopausal women ' was excluded due to exposure/outcome categories. Therefore, no specific symptom-related findings were extracted. |
| Lee 2013 | exposure/outcome categories | http://dx.doi.org/10.1016/j.maturitas.2013.06.007 | This study titled 'Muscle strength and quality are associated with severity of menopausal symptoms in peri- and post-menopausal women ' was excluded due to exposure/outcome categories. Therefore, no specific symptom-related findings were extracted. |
| Lee 2020 | exposure/outcome categories | https://doi.org/10.1097/GME.0000000000001662 | This study titled 'Prevalence of symptoms and associated factors across menopause status in Taiwanese women ' was excluded due to exposure/outcome categories. Therefore, no specific symptom-related findings were extracted. |
| Legorreta 2013 | exposure/outcome categories | https://doi.org/10.3109/13697137.2012.696288 | This study titled 'Age at menopause, motives for consultation and symptoms reported by 40–59-year-old Mexican women ' was excluded due to exposure/outcome categories. Therefore, no specific symptom-related findings were extracted. |
| Lermer 2011 | exposure/outcome categories | https://doi.org/10.1097/gme.0b013e3181ec58f8 | This study titled 'Somatic and affective anxiety symptoms and menopausal hot flashes ' was excluded due to exposure/outcome categories. Therefore, no specific symptom-related findings were extracted. |
| Li 2005 | lack of symptom-specific statistics | https://doi.org/10.1016/j.maturitas.2005.05.005 | Li et al. (2005) conducted a large population‑based study of 6,917 Swedish women to examine how sociodemographic, lifestyle, and health factors relate to overall somatic and psychological symptom burden. The analysis identified several predictors of higher symptom risk—such as unemployment, lack of exercise, unmarried status, high body weight, smoking, and cardiovascular disease—but focused on aggregate symptom categories rather than item‑level reporting. Although the study addressed menopausal symptom burden broadly, it did not provide symptom‑specific prevalence or severity statistics for individual symptoms. As a result, it was excluded from symptom‑level evidence extraction. |
| Li 2012 | exposure/outcome categories | http://dx.doi.org/10.1016/j.maturitas.2012.09.008 | This study titled 'Factors associated with the age of natural menopause and menopausal symptoms in Chinese women ' was excluded due to exposure/outcome categories. Therefore, no specific symptom-related findings were extracted. |
| Li 2013 | lack of symptom-specific statistics | https://doi.org/10.1016/j.maturitas.2012.09.008 | Li et al. conducted a large cross‑sectional study of 20,275 Chinese women aged 40–65 years, examining demographic and health factors associated with age at natural menopause and overall menopausal symptom severity using the Kupperman Menopause Index (KMI). The study reported general patterns—for example, sexual problems (57.05%), joint/muscle pain (53.29%), and insomnia (51.02%) were the most common symptoms in postmenopausal women—but focused primarily on associations with sociodemographic factors and total KMI scores. |
| Li 2016 | exposure/outcome categories | http://dx.doi.org/10.1097/MD.0000000000004466 | This study titled 'Perimenopausal syndrome and mood disorders in perimenopause: prevalence, severity, relationships, and risk factors ' was excluded due to exposure/outcome categories. Therefore, no specific symptom-related findings were extracted. |
| Li 2021 | lack of symptom-specific statistics | http://dx.doi.org/10.1097/GME.0000000000001726 | Li et al. (2021) investigated associations between sleep problems and the likelihood of having severe menopausal symptoms, using the Menopause Rating Scale to classify women as having severe vs. non‑severe symptoms. The study identified several sleep‑related factors (“feeling too hot,” pain, restless legs syndrome, use of sleep medication, difficulty staying awake) that were statistically associated with severe menopausal symptoms after propensity score matching. However, the article did not provide symptom‑specific prevalence, severity scores, or item‑level statistics for individual menopausal symptoms. Instead, symptoms were aggregated into a binary severity category (severe vs. non‑severe). Because of this lack of extractable symptom‑specific data, the paper was excluded from symptom‑level evidence extraction. |
| Lipasti 2021 | lack of symptom-specific statistics | https://doi.org/10.1016/j.maturitas.2021.09.003 | Lipasti et al. (2021) conducted a nationwide comparison of Finnish women aged 52–56 in 2000 vs. 2010 and found that the overall severity of climacteric symptoms increased over time, with women in 2010 reporting more moderate or severe symptoms. Although the study assessed 12 menopausal symptoms and presented trends in severity, it did not provide extractable, item‑level symptom‑specific prevalence or severity statistics required for symptom‑level evidence extraction. The authors focused on cohort differences and sociodemographic associations rather than detailed symptom‑specific reporting. As a result, the study was excluded for lack of symptom‑specific statistics, and no individual symptom findings were extracted. |
| Liu 2008 | exposure/outcome categories | https://doi.org/10.1097/gme.0b013e318154bda0 | This study titled 'The menopausal experience of Greek women living in Sydney ' was excluded due to exposure/outcome categories. Therefore, no specific symptom-related findings were extracted. |
| Logan 2023 | exposure/outcome categories | https://doi.org/10.1016/j.maturitas.2023.107853 | This study titled 'Menopausal symptoms in midlife Singaporean women: Prevalence rates and associated factors from the Integrated Women's Health Programme (IWHP). ' was excluded due to exposure/outcome categories. Therefore, no specific symptom-related findings were extracted. |
| Love 2022 | lack of symptom-specific statistics | The Journal of The North American Menopause Society, Volume 29, Issue 12, page 1475 (December 2022). | This study titled 'Identifying factors that predict menopause symptoms to support personalised care pathways ' was excluded due to lack of symptom-specific statistics . Therefore, no specific symptom-related findings were extracted. |
| Lu 2007 | exposure/outcome categories | https://doi.org/10.1080/13697130601159649 | This study titled 'The experience of menopausal symptoms by Arabic women in Sydney ' was excluded due to exposure/outcome categories. Therefore, no specific symptom-related findings were extracted. |
| Lu 2023 | exposure/outcome categories | https://doi.org/10.1186/s12905-023-02621-8 | This study titled 'Prevalence of menopausal symptoms and attitudes towards menopausal hormone therapy in women aged 40-60 years: a cross-sectional study. ' was excluded due to exposure/outcome categories. Therefore, no specific symptom-related findings were extracted. |
| Lui-Filho 2018 | exposure/outcome categories | DOI: 10.1097/GME.0000000000001022 | This study titled 'Risk factors associated with intensity of climacteric symptoms in Brazilian middle-aged women: a population-based household survey. ' was excluded due to exposure/outcome categories. Therefore, no specific symptom-related findings were extracted. |
| Lui-Filho 2019 | lack of symptom-specific statistics | not available | This study titled 'Risk factors associated with genitourinary syndrome of menopause in brazilian middle-aged women: a population-based household survey ' was excluded due to lack of symptom-specific statistics . Therefore, no specific symptom-related findings were extracted. |
| LuiFilho 2015 | lack of symptom-specific statistics | https://doi.org/10.1590/SO100-720320150005282 | This study titled 'Epidemiologia da menopausa e dos sintomas climatéricos em mulheres de uma região metropolitana no sudeste do Brasil: inquérito populacional domiciliar ' was excluded due to lack of symptom-specific statistics . Therefore, no specific symptom-related findings were extracted. |
| Martinez 2013 | lack of symptom-specific statistics | http://www.rrh.org.au | This study titled 'Urban-rural differences in Spanish menopausal women ' was excluded due to lack of symptom-specific statistics . Therefore, no specific symptom-related findings were extracted. |
| Mathew 2020 | incompatible reference groups | https://doi.org/10.4103/jmh.JMH_88_19 | Mathew et al. (2020) investigated morbidity patterns and symptom burden among menopausal women in rural Uttar Pradesh, India, identifying several factors associated with symptom severity. The study found high prevalence of musculoskeletal problems, psychological symptoms, vasomotor complaints, and genitourinary issues in this population. Symptom burden was closely linked to sociodemographic factors, with age, low educational status, and lower socioeconomic position contributing to poorer health outcomes. Lifestyle‑related factors such as physical inactivity and high parity further increased symptom severity. Overall, the study highlights the combined influence of socioeconomic disadvantage, limited health awareness, and lifestyle patterns on menopausal morbidity in rural Indian women. |
| Matsuo 2012 | incompatible reference groups | https://pubmed.ncbi.nlm.nih.gov/23660453/ | Matsuo et al. (2012) compared menopausal symptoms and psychosocial factors among Japanese and Filipino women and found distinct cultural patterns in symptom presentation. Japanese women reported higher levels of mental symptoms, whereas Filipino women more frequently experienced motor‑neurological and psychoneurological symptoms. Stressors also differed: Japanese women were more likely to report interpersonal stressors (e.g., caregiving burdens), while Filipino women more often reported household or financial stress. In both groups, stress severity was significantly associated with menopausal symptom (SMI) scores, and poorer marital relationships were linked to worse symptoms—particularly among Japanese women. Overall, the study highlights that psychological and social factor, shaped by cultural context, play a major role in symptom burden during menopause. |
| Matthews 2019 | exposure/outcome categories | doi: 10.1093/sleep/zsz042 | This study titled 'Racial/ethnic disparities in women's sleep duration, continuity, and quality, and their statistical mediators: Study of Women's Health Across the Nation. ' was excluded due to exposure/outcome categories. Therefore, no specific symptom-related findings were extracted. |
| Matthews 2021 | exposure/outcome categories | https://doi.org/10.1097/GME.0000000000001663 | This study titled 'Identifying women who share patterns of reproductive hormones, vasomotor symptoms, and sleep maintenance problems across the menopause transition: group-based multi-trajectory modeling in the Study of Women’s Health Across the Nation ' was excluded due to exposure/outcome categories . Therefore, no specific symptom-related findings were extracted. |
| Metintas 2010 | lack of symptom-specific statistics | https://pubmed.ncbi.nlm.nih.gov/20356316/ | Metintas et al. (2010) evaluated the Menopause Rating Scale (MRS) as a screening tool to identify rural Turkish women with severe menopausal symptoms, comparing it with the Kupperman Index. The study focused on validity, reliability, and appropriate cutoff scores, finding strong correlation between the two scales and identifying an MRS threshold score of 16 for predicting care‑seeking due to symptom severity. Although the study assessed severity classifications and associated factors (age, chronic disease, perceived poor health), it did not provide symptom‑specific prevalence values or item‑level statistics for individual menopausal symptoms. Therefore, it was excluded from symptom‑specific evidence extraction. |
| Misiker 2023 | lack of symptom-specific statistics | https://doi.org/10.1186/s12905-023-02442-9 | This study assessed overall severity of menopausal symptoms among middle‑aged women in Ethiopia, finding a high prevalence of menopausal symptoms (88.7%), with most participants classified as asymptomatic or mildly affected according to the Menopause Rating Scale. Although the authors identified associated factors (e.g., age and chronic disease history), the study did not report symptom‑specific prevalence or item‑level statistics, except noting that sexual problems were the most severe symptom category. Because of this absence of extractable symptom‑specific data, the paper was excluded from symptom‑level evidence extraction, and no individual symptom findings were included. |
| Mitchell 2010 | exposure/outcome categories | https://doi.org/10.3109/13697137.2010.483025 | This study titled 'Pain symptoms during the menopausal transition and early postmenopause ' was excluded due to exposure/outcome categories . Therefore, no specific symptom-related findings were extracted. |
| Mitchell 2015 | exposure/outcome categories | https://doi.org/10.3109/13697137.2015.1009436 | This study titled 'Hot flush severity during the menopausal transition and early postmenopause: beyond hormones ' was excluded due to exposure/outcome categories. Therefore, no specific symptom-related findings were extracted. |
| Mitchell 2017 | exposure/outcome categories | https://doi.org/10.1186/s40695-017-0030-x | This study titled 'Depressed mood during the menopausal transition: is it reproductive aging or is it life? ' was excluded due to exposure/outcome categories. Therefore, no specific symptom-related findings were extracted. |
| Moilanen 2010 | exposure/outcome categories | 10.1016/j.maturitas.2010.08.007 | This study titled 'Prevalence of Menopause Symptoms and their Association with Lifestyle Among Finnish middle-aged women ' was excluded due to exposure/outcome categories. Therefore, no specific symptom-related findings were extracted. |
| Monica 2011 | lack of symptom-specific statistics | https://doi.org/10.3109/13697137.2011.583733 | This study titled 'body mass index and hot flashes in postmenopausal women: a meta-analysis ' was excluded due to lack of symptom-specific statistics . Therefore, no specific symptom-related findings were extracted. |
| Monterrosa 2008 | exposure/outcome categories | https://doi.org/10.1016/j.maturitas.2007.12.002 | This study titled 'Increased menopausal symptoms among Afro-Colombian women as assessed with the Menopause Rating Scale ' was excluded due to exposure/outcome categories. Therefore, no specific symptom-related findings were extracted. |
| Monterrosa 2009 | exposure/outcome categories | https://doi.org/10.1080/09513590902972091 | This study titled 'Quality of life impairment among postmenopausal women varies according to race ' was excluded due to exposure/outcome categories. Therefore, no specific symptom-related findings were extracted. |
| Monterrosa-Castro 2012 | exposure/outcome categories | http://dx.doi.org/10.1097/gme.0b013e31826e7649 | This study titled 'Assessment of sleep quality and correlates in a large cohort of Colombian women around menopause ' was excluded due to exposure/outcome categories. Therefore, no specific symptom-related findings were extracted. |
| Monterrosa-Castro 2013 | exposure/outcome categories | https://doi.org/10.1097/gme.0b013e31826e7649 | This study titled 'Assessment of sleep quality and correlates in a large cohort of Colombian women around menopause ' was excluded due to exposure/outcome categories. Therefore, no specific symptom-related findings were extracted. |
| Monterrosa-Castro 2013_2 | exposure/outcome categories | http://dx.doi.org/10.1016/j.maturitas.2013.01.009 | This study titled 'Prevalence of insomnia and related factors in a large mid-aged female Colombian sample ' was excluded due to exposure/outcome categories. Therefore, no specific symptom-related findings were extracted. |
| Monterrosa-Castro 2016 | exposure/outcome categories | http://dx.doi.org/10.1155/2016/8790691 | This study titled 'Differential Item Functioning of the Psychological Domain of the Menopause Rating Scale. ' was excluded due to exposure/outcome categories. Therefore, no specific symptom-related findings were extracted. |
| Morse 2019 | exposure/outcome categories | https://doi.org/10.1016/j.nurpra.2018.10.020 | This study titled 'Exercise and Insomnia Risk in Middle-Aged Women ' was excluded due to exposure/outcome categories. Therefore, no specific symptom-related findings were extracted. |
| Muharam 2018 | exposure/outcome categories | http://dx.doi.org/10.1016/j.mefs.2017.08.003 | This study titled 'Depression and its link to other symptoms in menopausal transition ' was excluded due to exposure/outcome categories. Therefore, no specific symptom-related findings were extracted. |
| Murphy 2022 | lack of symptom-specific statistics | https://doi.org/10.1017/S0029665122001422 | Murphy et al. (2022) surveyed 238 symptomatic postmenopausal women in Ireland to examine their menopausal symptoms, BMI patterns, and health behaviours. The most severe symptoms reported were low sex drive (33.2%), vaginal dryness (26.5%), insomnia (26.1%), and hot flushes (26%), and most women (67%) said symptoms interfered with daily activities. The mean modified Kupperman Index score was 22.55 ± 8.48, with higher symptom scores in overweight and obese women compared with those of normal BMI. Although the study highlights relative severity rankings, it does not provide full symptom‑specific prevalence distributions, and thus is appropriately categorised as lacking detailed extractable symptom‑level statistics. |
| Murphy 2022_2 | lack of symptom-specific statistics | http://dx.doi.org/10.1017/S0029665122001434 | Murphy et al. (2022) examined whether adherence to a Mediterranean dietary pattern was associated with menopausal symptom burden among peri‑ and postmenopausal women in Ireland. The study included 304 women, who completed the Menopause Rating Scale (MRS) and the Mediterranean Diet Assessment Tool (MDAT), with most reporting moderate adherence to the diet. Mean total MRS scores were 15.6 ± 6.9 (perimenopausal) and 11.6 ± 7.9 (postmenopausal), indicating modest symptom severity overall. While the study explored associations between dietary pattern and symptom intensity, it did not report symptom‑specific prevalence or item‑level statistics, so it is classified as lacking extractable symptom‑specific data. |
| Nagaraj 2021 | exposure/outcome categories | https://doi.org/10.4103/jmh.JMH_196_20 | This study titled 'Experience and Perceptions Regarding Menopause among Rural Women: A CrossSectional HospitalBased Study in South Karnataka ' was excluded due to exposure/outcome categories. Therefore, no specific symptom-related findings were extracted. |
| Nancy 2014 | lack of symptom-specific statistics | abstract published | This study titled 'Hot flash severity across the menopausal transition: observations from the seattle midlife women’s health study ' was excluded due to lack of symptom-specific statistics . Therefore, no specific symptom-related findings were extracted. |
| Nazarpour 2018 | exposure/outcome categories | https://doi.org/10.1080/03630242.2017.1414100 | This study titled 'The relationship between menopausal symptoms and sexual function ' was excluded due to exposure/outcome categories. Therefore, no specific symptom-related findings were extracted. |
| Nazarpour 2020 | exposure/outcome categories | https://doi.org/10.1186/s12905-020-00960-4 | This study titled 'Factors associated with quality of life of postmenopausal women living in Iran. ' was excluded due to exposure/outcome categories. Therefore, no specific symptom-related findings were extracted. |
| Nelson 2011 | exposure/outcome categories | https://doi.org/10.1097/gme.0b013e318214d69d | This study titled ' Effects of reproductive history on symptoms of menopause: a brief report ' was excluded due to exposure/outcome categories. Therefore, no specific symptom-related findings were extracted. |
| Neri 1997 | exposure/outcome categories | https://doi.org/10.3109/01674829709080692 | This study titled 'Coping style and climacteric symptoms in a clinical sample of postmenopausal women. ' was excluded due to exposure/outcome categories. Therefore, no specific symptom-related findings were extracted. |
| Neutzling 2020 | lack of symptom-specific statistics | DOI:10.1097/GME.0000000000001524 | Neutzling et al. (2020) examined the relationship between common mental disorders (CMDs), sleep quality, and menopausal symptoms in a population-based sample of midlife women in Southern Brazil. The study found a strong association: women with CMDs and poor sleep quality reported significantly more severe menopausal symptoms compared with those without these conditions. Although key symptom domains were assessed, the paper did not provide detailed symptom‑specific prevalence statistics, limiting extraction of item‑level data. Overall, the findings highlight the importance of addressing mental health and sleep disturbances when managing menopausal symptom burden. |
| Noll 2022 | exposure/outcome categories | https://doi.org/10.1016/j.maturitas.2021.10.015 | This study titled 'Life habits of postmenopausal women: Association of menopause symptom intensity and food consumption by degree of food processing ' was excluded due to exposure/outcome categories. Therefore, no specific symptom-related findings were extracted. |
| Nowakowska 2015 | lack of symptom-specific statistics | http://dx.doi.org/10.13075/mp.5893.00243 | Nowakowska et al. (2015) found a statistically significant association between the severity of perimenopausal symptoms (Kupperman Index) and women’s workplace functioning, gynecological care access, use of hormone therapy, and overall life satisfaction. Using Pareto‑Lorenz analysis, women identified hot flashes, irritability, reduced concentration/coordination, sleep disturbances, and excessive sweating as the symptoms most disruptive to work performance. The authors observed that symptom burden was meaningfully related to life satisfaction and workplace effectiveness but did not provide detailed symptom‑specific prevalence statistics, only highlighting which symptoms women perceived as most impactful. This makes the study appropriate to classify as lacking extractable, symptom‑level quantitative data. |
| Ogwumike 2016 | exposure/outcome categories | http://dx.doi.org/10.1016/j.hkpj.2015.06.001 | This study titled 'Musculoskeletal pain among postmenopausal women in Nigeria:Association with overall and central obesity ' was excluded due to exposure/outcome categories. Therefore, no specific symptom-related findings were extracted. |
| Oi 2012 | exposure/outcome categories | http://dx.doi.org/10.1111/j.1468-2494.2012.00722.x | This study titled ' A study of menopausal symptoms in relation to habits of smoking and make-up using in Japanese women aged 35–59 ' was excluded due to exposure/outcome categories. Therefore, no specific symptom-related findings were extracted. |
| Ojeda 2010 | exposure/outcome categories | https://doi.org/10.3109/13697130903576297 | This study titled 'Severe menopausal symptoms in mid-aged Latin American women can be related to their indigenous ethnic component. ' was excluded due to exposure/outcome categories. Therefore, no specific symptom-related findings were extracted. |
| Ojeda 2011 | exposure/outcome categories | https://doi.org/10.3109/13697130903576297 | This study titled 'Severe menopausal symptoms in mid-aged Latin American women can be related to their indigenous ethnic component ' was excluded due to exposure/outcome categories. Therefore, no specific symptom-related findings were extracted. |
| Olajubu 2017 | lack of symptom-specific statistics | https://doi.org/10.1080/13697137.2017.1373336 | Olajubu et al. (2017) found a very high prevalence of menopausal symptoms (96.5%) among working women aged ≥45 in a Nigerian university. The most common symptoms were muscle pain (81.5%), night sweating (80%), and hot flushes, which were also rated the most severe. Only 27% of participants reported excellent work ability, and symptom severity was significantly negatively correlated with perceived work ability (r = –0.311, p < .001). While the study identifies key symptoms and broad prevalence patterns, it does not provide granular symptom‑specific statistics (e.g., frequency distributions across all MRS items), making it appropriate to classify as lacking detailed extractable symptom‑specific data. |
| Olowokere 2021 | lack of symptom-specific statistics | https://doi-org.ezproxy-prd.bodleian.ox.ac.uk/10.1177/2053369120971427 | Olowokere et al. (2021) found that most rural Nigerian women experienced moderate menopausal symptoms (66.4%), with smaller proportions reporting severe (15.5%) or very severe (0.4%) symptoms. The majority of participants (58.3%) had poor lifestyle practices, and regression analysis showed that exercise, nutrition, and substance use were significant predictors of symptom severity. Although the study assessed symptom burden using a modified MRS, it did not provide symptom‑specific prevalence rates, making it unsuitable for detailed symptom‑level extraction. It concluded that improving lifestyle behaviours may help reduce menopause‑related symptoms in rural populations. |
| Onya 2018 | lack of symptom-specific statistics | no full text available from library | This study titled 'Biological and environmental correlates of post menopausal depression among Gopd attendees in Fmc, Lokoja, Nigeria ' was excluded due to lack of symptom-specific statistics. Therefore, no specific symptom-related findings were extracted. |
| Palacios 2010 | exposure/outcome categories | https://doi.org/10.3109/13697137.2010.507886 | This study titled 'Age of menopause and impact of climacteric symptoms by geographical region ' was excluded due to exposure/outcome categories. Therefore, no specific symptom-related findings were extracted. |
| Panichkul 2017 | exposure/outcome categories | https://doi.org/10.5372/1905-7415.1103.551 | This study titled 'Menopause-related symptoms and quality of life in peri- and postmenopausal women in Thailand: a multicenter study ' was excluded due to exposure/outcome categories. Therefore, no specific symptom-related findings were extracted. |
| Papazisis 2022 | lack of symptom-specific statistics | https://doi.org/10.1016/j.maturitas.2021.10.014 | Papazisis et al. (2022) reported that 27.1% of post‑menopausal women screened positive for moderate‑to‑severe depressive symptoms (BDI‑II ≥ 20) in a large web‑based sample of 502 Greek women. The study found that age <55, unemployment, smoking, and a prior history of depression were significant independent predictors of depression, with smokers having notably higher odds of moderate depressive symptoms. In subgroup analyses, obesity and lack of health insurance further increased the risk of severe depressive symptoms. As the study focused on depression risk rather than menopause‑related symptom prevalence, it lacks symptom‑specific menopausal data and is thus categorized accordingly. |
| Park 2001 | incompatible reference groups | https://doi.org/10.1300/J013v34n01_02 | Park et al. (2001) examined climacteric symptoms among 2,807 naturally postmenopausal Korean women and assessed associations with lifestyle and demographic factors. The most commonly reported symptoms were hot flashes, backaches, fatigue, and memory lapses, occurring in both peri‑ and postmenopausal groups. Analysis of associated factors showed no significant differences in vasomotor symptom severity across lifestyle behaviors, including smoking and physical activity. However, alcohol use and a preference for wheat were linked to higher levels of physical symptoms, indicating limited but specific lifestyle–symptom associations. Overall, symptom burden was influenced only modestly by lifestyle factors in this population. |
| Pastore 2004 | incompatible reference groups | https://doi.org/10.1016/j.maturitas.2004.06.019 | Pastore et al. (2004) examined urogenital symptoms—dryness, itching/irritation, discharge, and dysuria—among more than 98,000 postmenopausal women in the Women’s Health Initiative. Hispanic ethnicity, obesity, and treated diabetes were significantly associated with higher odds of multiple urogenital symptoms. Use of vaginal cream hormone therapy was also associated with increased reports of severe dryness and irritation/itching. Conversely, age, years since menopause, sexual activity, smoking, marital status, gravidity, and type of menopause were not associated with symptom prevalence. Overall, the study identified metabolic factors and ethnicity as key correlates of urogenital symptom burden in postmenopausal women. |
| Perez 2009 | lack of symptom-specific statistics | https://doi.org/10.1016/j.maturitas.2008.10.003 | Pérez et al. (2009) examined over 10,500 Spanish women aged 45–65 and found that the most common menopausal symptoms were hot flushes (51.4%), insomnia (45.7%), and irritability (42.2%). Symptom severity varied, with 3.3% experiencing severe symptoms, while nearly half (44.8%) reported none. Risk factors for more severe symptoms included higher age, higher BMI, smoking, lower social class, and low dairy intake, and postmenopausal women generally had significantly higher symptom rates than perimenopausal women. Because this paper reports only broad symptom prevalence and severity categories—not granular symptom‑specific statistical detail—it is classified as lacking extractable, symptom‑specific quantitative data. |
| Perez-Roncero 2013 | lack of symptom-specific statistics | http://dx.doi.org/10.1016/j.enfcli.2013.09.003 | Pérez‑Roncero et al. (2013) found that the most prevalent menopausal symptoms among middle‑aged women in Zaragoza were musculoskeletal symptoms, followed by hot flushes and perspiration. Symptom severity (somatic, psychological, and urogenital) was significantly higher in postmenopausal women and increased with age, BMI, age at menopause, and partner age. Multivariable regression showed that more severe symptoms were associated with menopausal stage, psychiatric treatment, sexual relationship difficulties, urinary incontinence, partner alcohol abuse, and a history of gender violence. As the study focused on correlates and predictors rather than providing detailed symptom‑specific prevalence values, it is considered to have insufficient extractable symptom‑specific statistics. |
| Pien 2008 | exposure/outcome categories | https://doi.org/10.5665/sleep/31.7.991 | This study titled 'Predictors of Sleep Quality in Women in the Menopausal Transition ' was excluded due to exposure/outcome categories. Therefore, no specific symptom-related findings were extracted. |
| Pimenta 2011 | lack of symptom-specific statistics | https://doi.org/10.1016/j.maturitas.2011.05.005 | Pimenta et al. (2011) found that perceived control was the strongest predictor of lower self‑reported severity, frequency, and intensity of hot flashes and night sweats among symptomatic midlife women. The study also identified hormone therapy use and coffee intake as additional predictors associated with less severe vasomotor symptoms. Using structural equation modelling across 20 lifestyle, reproductive, and sociodemographic variables, the model explained 67–72% of the variance in hot‑flash and night‑sweat severity. As this paper focuses on predictors rather than prevalence, it does not provide symptom‑specific frequency statistics, making it appropriate to classify as lacking symptom‑specific data. |
| Pimenta 2012 | exposure/outcome categories | https://doi.org/10.1080/08952841.2012.639653 | This study titled 'Menopause Symptoms' Predictors: The Influence of Lifestyle, Health- and Menopause-Related, and Sociodemographic Characteristics ' was excluded due to exposure/outcome categories. Therefore, no specific symptom-related findings were extracted. |
| Pimenta 2012 | exposure/outcome categories | 10.1016/j.maturitas.2012.04.006 | This study titled 'Menopausal symptoms: do life events predict severity of symptoms in peri and post menopause? ' was excluded due to exposure/outcome categories. Therefore, no specific symptom-related findings were extracted. |
| Pimenta 2014 | exposure/outcome categories | https://doi.org/10.1007/s10943-013-9696-z | This study titled 'Menopausal Symptoms: Is Spirituality Associated with the Severity of Symptoms? ' was excluded due to exposure/outcome categories. Therefore, no specific symptom-related findings were extracted. |
| Prado 2008 | incompatible reference groups | https://pubmed.ncbi.nlm.nih.gov/19350167/ | Prado and colleagues reported that the mean age of natural menopause was 47.5 years among Brazilian women, while the mean menarche age was 12.7 years. An earlier onset of menopause (before age 47) was associated with higher follicle‑stimulating hormone (FSH) levels, which increased the likelihood of earlier menopausal timing. In contrast, age and luteinizing hormone (LH) were negatively associated with early menopause. The study found no significant associations between menopausal age and sociodemographic factors, ethnicity, anxiety, or age at menarche. Overall, FSH was the only strong biomarker linked to earlier menopause in this cohort. |
| Prairie 2011 | exposure/outcome categories | 10.1089/jwh.2014.4798 | This study titled 'Symptoms of depressed mood, disturbed sleep and sexual problems in midlife women: cross-sectional data from the Study of Women's Health Across the Nation (SWAN) ' was excluded due to exposure/outcome categories. Therefore, no specific symptom-related findings were extracted. |
| Prairie 2015 | exposure/outcome categories | 10.1089/jwh.2014.4798 | This study titled 'Symptoms of depressed mood, disturbed sleep and sexual problems in midlife women: cross-sectional data from the Study of Women's Health Across the Nation ' was excluded due to exposure/outcome categories. Therefore, no specific symptom-related findings were extracted. |
| Rahman 2020 | incompatible reference groups | https://doi.org/10.3329/bmrcb.v46i2.49020 | Rahman et al. (2020) found that age, BMI, and physical activity levels were important factors associated with menopausal symptoms in Bangladeshi women. Somato‑vegetative symptoms—such as hot flashes, heart discomfort, and sleep problems—were common and tended to vary by both age and BMI. Irritability and heart discomfort were significantly associated with age, while joint and muscular discomfort, hot flushes, sweating, and heart discomfort showed significant associations with BMI (p < 0.05). Psychological and urogenital symptoms were also prevalent and mild to moderate in most respondents, indicating that both physiological and lifestyle factors shape overall symptom burden. |
| Reed 2009 | exposure/outcome categories | https://doi.org/10.1016/j.maturitas.2009.01.002 | This study titled 'Depressive symptoms and menopausal burden in the midlife ' was excluded due to exposure/outcome categories. Therefore, no specific symptom-related findings were extracted. |
| Ri 2022 | exposure/outcome categories | http://dx.doi.org/10.1097/GME.0000000000001889 | This study titled 'Hypertensive disorders of pregnancy increase the risk of future menopausal hot flashes in Japanese women: results from the Japan Nurses’ Health Study ' was excluded due to exposure/outcome categories. Therefore, no specific symptom-related findings were extracted. |
| Rindner 2017 | lack of symptom-specific statistics | https://doi.org/10.1186/s12905-017-0480-1 | Rindner et al. (2017) found that women aged 45–55 attending primary care frequently reported exhaustion, depressive mood, muscle and joint problems, sleep issues, and sexual problems. About half of the women experienced heart discomfort, highlighting the need for cardiovascular risk assessment in this age group. Depression and increasing age were the strongest predictors of more severe menopausal symptoms. The authors recommended proactive screening for musculoskeletal, sexual, sleep, cardiovascular, and mental health concerns in midlife women presenting to primary care. |
| Rouen 2009 | exposure/outcome categories | n/a (UMI: 3354092) | This study titled 'Study of women veterans in menopause. ' was excluded due to exposure/outcome categories. Therefore, no specific symptom-related findings were extracted. |
| Sabia 2008 | lack of symptom-specific statistics | https://doi.org/10.1016/j.maturitas.2008.04.004 | Sabia et al. (2008) identified multiple reproductive, hormonal, lifestyle, and medical factors associated with the onset of menopausal symptoms in over 28,000 participants of the French E3N cohort. The risk of developing symptoms was lower among women with higher education, longer menstrual cycles, higher parity, and current oral contraceptive use, as well as among underweight, overweight, and obese women during post‑menopause. Symptom risk was higher among smokers, alcohol consumers, and women with conditions such as depression, migraine, benign thyroid disease, and atopy; diets high in rapidly absorbed sugars and frequent snacking also increased risk. Because the paper focuses on factors influencing risk of onset, it does not provide symptom‑specific prevalence statistics, and is therefore categorized as lacking symptom‑level data for evidence tables. |
| Saccomani 2017 | exposure/outcome categories | https://doi.org/10.1097/GME.0000000000000884 | This study titled 'Does obesity increase the risk of hot flashes among midlife women?: a population-based study ' was excluded due to exposure/outcome categories. Therefore, no specific symptom-related findings were extracted. |
| Salazar-Pousada 2017 | lack of symptom-specific statistics | https://doi.org/10.1097/GME.0000000000000924 | Salazar‑Pousada et al. reported a high prevalence of menopausal symptoms among low‑income mid‑aged women in Ecuador, with muscle–joint pain (75%), changes in skin texture (74.3%), and vaginal dryness (71.4%) being the most common complaints. The sample showed marked socioeconomic vulnerability, including very low awareness of menopause‑related information—98.6% of women did not know what menopause is or when it typically begins. Symptom severity was higher among older, married, postmenopausal, and less educated women, while those with less menopause‑related knowledge paradoxically scored lower on symptom severity scales. The authors emphasized the need for targeted educational interventions for high‑risk, low‑income populations to improve health literacy and long‑term outcomes. |
| Sanchez-Zarza 2023 | exposure/outcome categories | https://doi.org/10.1080/09513590.2023.2235427 | This study titled 'Prevalence of menopausal symptoms and severity related factors among mid-aged Paraguayan women as measured with the 10-item Cervantes Scale. ' was excluded due to exposure/outcome categories. Therefore, no specific symptom-related findings were extracted. |
| Sayakhot 2012 | exposure/outcome categories | https://dx.doi.org/10.1097/gme.0b013e31825fd14e | This study titled 'Cross-cultural study: experience, understanding of menopause, and related therapies in Australian and Laotian women ' was excluded due to exposure/outcome categories. Therefore, no specific symptom-related findings were extracted. |
| Sayon Orea 2015 | exposure/outcome categories | DOI: 10.1097/GME.0000000000000378 | This study titled 'Adherence to Mediterranean dietary pattern and menopausal symptoms in relation to overweight/obesity in Spanish perimenopsusal and postmenopausal women ' was excluded due to exposure/outcome categories. Therefore, no specific symptom-related findings were extracted. |
| Schnatz 2006 | exposure/outcome categories | 10.1097/01.ogx.0000201923.84932.90 | This study titled 'Menopausal Symptoms in Hispanic Women and the Role of Socioeconomic Factors ' was excluded due to exposure/outcome categories. Therefore, no specific symptom-related findings were extracted. |
| Sharami 2022 | lack of symptom-specific statistics | https://doi.org/10.1186/s12905-022-01715-z | Sharami et al. (2022) found that reproductive history was significantly associated with somatic, psychological, and urogenital menopausal symptom severity in women aged 35–65. Women with a history of abortion had higher overall and psychological symptom scores, while those with a greater number of children showed significantly higher somatic symptom scores. Although the study confirmed statistically meaningful associations, it did not provide detailed symptom‑specific prevalence rates. The authors concluded that reproductive factors may help identify women at elevated risk for more severe menopausal symptoms later in life. |
| SharanyaShre 2016 | lack of symptom-specific statistics | 10.4103/0975-7406.171728 | Sharanya Shre et al. (2016) found that hot flushes (24%), heart‑related complaints (49%), and disturbed sleep (48%) were among the most commonly reported menopausal symptoms in postmenopausal women in Chennai, India. The study reported no significant difference in overall menopausal symptom severity between obese and non‑obese women based on MRS scores. Married women showed significantly higher rates of overweight/obesity (64%) compared with widows (41%). Overall, the authors emphasized the need for accessible, technology‑supported education to help women manage chronic postmenopausal symptoms. |
| Shea 2020 | exposure/outcome categories | https://doi.org/10.1097/GME.0000000000001540 | This study titled ' Depression, hormone therapy, and the menopausal transition among women aged 45 to 64 years using Canadian Longitudinal Study on aging baseline data ' was excluded due to exposure/outcome categories. Therefore, no specific symptom-related findings were extracted. |
| Sheereen 2022 | exposure/outcome categories | https://doi.org/10.4103/jfmpc.jfmpc_1377_22 | This study titled 'Community-based appraisal of menopause-specific health problems and quality of life among women of rural Western Maharashtra. ' was excluded due to exposure/outcome categories. Therefore, no specific symptom-related findings were extracted. |
| Shivappa 2016 | exposure/outcome categories | https://doi.org/10.1017/S0007114516002853 | This study titled 'Association between inflammatory potential of diet and risk of depression in middle-aged women: the Australian Longitudinal Study on Women’s Health ' was excluded due to exposure/outcome categories. Therefore, no specific symptom-related findings were extracted. |
| Shukla 2018 | incompatible reference groups | https://doi.org/10.4103/jmh.JMH_34_16 | Shukla et al. (2018) reported a 47% prevalence of postmenopausal symptoms among rural Indian women, with physical symptoms being most common (91.5%), followed by psychosocial (44.7%) and vasomotor symptoms (21.3%). Age at menopause averaged 44.9 years, and couples were generally aware of menopause, but most relied on avoidant emotional coping rather than active coping strategies. The presence of symptoms was associated with mild‑to‑moderate reductions in quality of life, indicating that symptom burden directly affects well‑being. The study suggests that limited coping behaviors and acceptance of symptoms as “natural” may contribute to unmanaged symptom severity. |
| Shyu 2012 | incompatible reference groups | 10.1097/jnr.0b013e318265619b | Shyu et al. (2012) found that age, religion, smoking, exercise, and comorbidities were independently associated with the presence of menopausal symptoms in Taiwanese women. Women with menopausal symptoms had significantly higher odds of poor physical and mental health‑related quality of life, even after adjustment for confounders. The study also showed that symptomatic women were more likely to use healthcare services, including outpatient care, traditional Chinese medicine, emergency services, and hospitalization. Overall, menopausal symptoms were associated with both diminished quality of life and increased healthcare utilization. |
| Simpkins 2009 | exposure/outcome categories | https://doi.org/10.1016/j.maturitas.2009.06.002 | This study titled 'Role of ethnicity in the expression of features of hot flashes ' was excluded due to exposure/outcome categories. Therefore, no specific symptom-related findings were extracted. |
| Singhania 2020 | exposure/outcome categories | https://doi.org/10.4103/jmh.JMH_123_19 | This study titled 'Association of Menopausal Symptoms with Overweight and Obesity among Rural Middle Aged Women in North India: A Population Based Study' was excluded due to exposure/outcome categories. Therefore, no specific symptom-related findings were extracted. |
| Sitini 2023 | exposure/outcome categories | https://doi.org/10.1080/13697137.2023.2246887 | This study titled 'Prevalence, impact and management of postmenopausal symptoms among postmenopausal women in Rwanda ' was excluded due to exposure/outcome categories. Therefore, no specific symptom-related findings were extracted. |
| Skrzypulec 2010 | lack of symptom-specific statistics | https://doi.org/10.3109/13697131003597019 | Skrzypulec et al. (2010) found that higher levels of physical activity were strongly associated with fewer and less severe climacteric symptoms in menopausal women. Over half of highly active participants (52.1%) reported no menopausal symptoms, and symptom reduction was consistent across work, transportation, domestic, and leisure‑time activity domains. Women with moderate or high activity levels consistently reported better wellbeing and lower symptom burden compared with those with low activity levels. The study underscores physical activity as a key modifiable factor linked to reduced menopausal symptom severity. |
| Smith 2018 | incompatible reference groups | 10.1097/GME.0000000000000959 | Smith et al. (2018) used a Bayesian network approach to examine how various biological and lifestyle factors relate to hot flashes in midlife women. Hormone levels—including estrogen, progesterone, and testosterone—were the factors most consistently linked to the presence, timing, severity, and frequency of hot flashes. Hormone levels were sometimes indirectly related to BMI, but showed no direct associations with other risk factors such as race, income, smoking, alcohol use, or physical activity. Overall, the study highlights hormonal regulation as the central determinant of hot flash patterns, with other demographic and lifestyle variables playing minimal or indirect roles. |
| Song 2022 | lack of symptom-specific statistics | https://doi.org/10.1155/2022/2560053 | Song et al. (2022) reported that the most common menopausal symptoms among women aged 40–65 were fatigue (72.9%), sleep disturbance (67%), and hot flashes/night sweats (65%). Sleep disturbance was highly prevalent, with 66.9% of women meeting criteria for poor sleep quality (PSQI > 5). Increasing menopausal symptom severity was strongly associated with worsening sleep: women with mild, moderate, and severe symptoms had approximately 3‑, 7‑, and 17‑fold higher odds of sleep disturbance, respectively. Although this article has since been retracted, the reported data suggested a strong link between menopausal symptom burden and impaired sleep. |
| Stojanovska 2014 | incompatible reference groups | 10.1016/j.maturitas.2014.01.006 | Stojanovska et al. (2014) reported that physical activity is a key factor influencing menopausal symptom severity, with regular exercise helping to reduce psychological, vasomotor, somatic, and sexual symptoms. The study suggests that active women tend to experience fewer and less intense menopausal symptoms, positioning exercise as an effective non‑hormonal management option. Physical activity was also linked to improvements in mood, sleep, hot flashes, and musculoskeletal discomfort, identifying it as an important modifiable lifestyle factor influencing overall symptom burden. |
| Stojanovska 2015 | incompatible reference groups | Page 1391 of abstracts | This study titled 'Menopause and avoidance of physical activity ' was excluded due to incompatible reference groups. Therefore, no specific symptom-related findings were extracted. |
| Su 2021 | exposure/outcome categories | https://doi.org/10.1097/GME.0000000000001874 | This study titled 'Menopausal symptoms among Chinese and Japanese women: differences and similarities ' was excluded due to exposure/outcome categories. Therefore, no specific symptom-related findings were extracted. |
| Su 2022 | exposure/outcome categories | 10.1097/GME.0000000000001874 | This study titled 'Menopausal symptoms among Chinese and Japanese women: differences and similarities. ' was excluded due to exposure/outcome categories. Therefore, no specific symptom-related findings were extracted. |
| Suau 2005 | exposure/outcome categories |  | This study titled 'Depressive symptoms and risk factors among perimenopausal women. ' was excluded due to exposure/outcome categories. Therefore, no specific symptom-related findings were extracted. |
| Sun 2014 | exposure/outcome categories | http://dx.doi.org/10.1016/j.jpsychores.2013.12.002 | This study titled 'Sleep disturbance and correlates in menopausal women in Shanghai ' was excluded due to exposure/outcome categories. Therefore, no specific symptom-related findings were extracted. |
| Sun 2021 | lack of symptom-specific statistics | https://doi.org/10.1177/1010539520960987 | Sun et al. (2021) found that women with higher health literacy reported significantly lower overall menopausal symptom burden, indicating a protective role of health‑related knowledge and self‑management skills. The study highlighted substantial variation in symptom experience, noting that women with limited health literacy were more likely to report greater physical and psychological discomfort during menopause. Although the article did not provide detailed symptom‑specific prevalence, it emphasized that improving health literacy may help mitigate symptom severity and enhance coping strategies. |
| Sut 2017 | lack of symptom-specific statistics | 42_sut_ABSTRACT_10_1 | This study titled 'Severity of Menopausal Symptoms in Climacteric Turkish Women ' was excluded due to lack of symptom-specific statistics . Therefore, no specific symptom-related findings were extracted. |
| Sweed 2012 | lack of symptom-specific statistics | https://doi.org/10.26719/2012.18.3.213 | Sweed et al. (2012) reported very high prevalence of postmenopausal symptoms among Egyptian women aged over 65, with joint pain (90.3%), sleep problems (84%), and physical/mental exhaustion (80%) being the most common complaints. Symptom severity increased significantly with older age, longer duration since menopause, and a higher number of chronic diseases, but was not related to age at menopause. Overall, the study highlights a heavy late‑life symptom burden and the strong influence of age‑related health conditions on menopausal symptom scores in elderly women. |
| Takenaka 2021 | exposure/outcome categories | 10.1265/jjh.20004 | This study titled 'Investigation of Relationships among Health Literacy, Social Determinants of Health, Menopausal Symptoms, Lifestyle related Diseases and Depression in Menopausal Women ' was excluded due to exposure/outcome categories. Therefore, no specific symptom-related findings were extracted. |
| Tan 2014 | lack of symptom-specific statistics | http://www.biomedcentral.com/1472-6874/14/38 | Tan et al. (2014) found that physically active women reported significantly lower overall menopausal symptom scores, including reductions in somatic, psychological, and urogenital complaints. Although physical activity did not influence vasomotor symptoms, it was associated with fewer sleep problems, less joint/muscle discomfort, and reduced vaginal dryness. BMI was generally not associated with symptom severity, except for a higher likelihood of depressive mood among overweight and obese women. Overall, the study highlights physical activity—rather than weight—as a key modifiable factor linked to reduced menopausal symptom burden. |
| Taskiran 2021 | exposure/outcome categories | https://doi.org/10.1007/s43032-021-00545-y | This study titled 'Individual Characteristics Associated with Menopausal Symptom Severity and Menopause-Specific Quality of Life: A Rural Perspective ' was excluded due to exposure/outcome categories. Therefore, no specific symptom-related findings were extracted. |
| Taylor 2010 | exposure/outcome categories |  | This study titled 'Do attitudes towards aging and menopause vary by menopausal status, age and symptoms? ' was excluded due to exposure/outcome categories. Therefore, no specific symptom-related findings were extracted. |
| Terauchi 2020 | lack of symptom-specific statistics | abstract | This study titled 'Body Mass Index and Percentage Body Fat Are Negatively Associated with Severe Dyspareunia in Japanese Post-menopausal Women ' was excluded due to lack of symptom-specific statistics . Therefore, no specific symptom-related findings were extracted. |
| Terauchi 2022 | exposure/outcome categories | 10.1111/jog.15418 | This study titled 'Body weight and body fat are negatively associated with severe dyspareunia in postmenopausal women ' was excluded due to exposure/outcome categories. Therefore, no specific symptom-related findings were extracted. |
| Thapa 2020 | lack of symptom-specific statistics | https://doi.org/10.1080/03630242.2019.1643815 | Thapa (2020) found a high prevalence of menopausal symptoms among Cambodian women, with somatic and psychological symptoms occurring more frequently than urogenital symptoms across all menopausal stages. The most common symptoms were physical and mental exhaustion (88.1%), irritability (85.9%), and sleep problems (82.5%). Symptom severity was significantly higher among postmenopausal women and was associated with lower income, high parity, history of abortion, calcium supplement use, and chronic conditions such as heart disease and rheumatoid arthritis. Overall, the study highlights a substantial symptom burden and the need for targeted support for Cambodian women. |
| Tong 2023 | exposure/outcome categories | https://doi.org/10.1080/03630242.2023.2188100 | This study titled 'High levels of physical activity are associated with a reduced likelihood of depressive symptoms in postmenopausal women. ' was excluded due to exposure/outcome categories. Therefore, no specific symptom-related findings were extracted. |
| Urrunaga-Pastor 2022 | exposure/outcome categories | http://dx.doi.org/10.1097/GME.0000000000001973 | This study titled 'Female orgasmic dysfunction and severe climacteric symptomatology in women aged 40 to 59years: an independent association from an analysis of a multicenter Latin American study ' was excluded due to exposure/outcome categories. Therefore, no specific symptom-related findings were extracted. |
| Valadares 2016 | exposure/outcome categories | 10.1097/GME.0000000000000783 | This study titled 'Is the age at menopause a cause of sexual dysfunction? A Brazilian population-based study ' was excluded due to exposure/outcome categories. Therefore, no specific symptom-related findings were extracted. |
| Waidyasekera 2009 | exposure/outcome categories | 10.1097/gme.0b013e31817a8abd | This study titled 'Menopausal symptoms and quality of life during the menopausal transition in Sri Lankan women. ' was excluded due to exposure/outcome categories. Therefore, no specific symptom-related findings were extracted. |
| Wang 2013 | exposure/outcome categories | http://dx.doi.org/10.1016/j.maturitas.2013.04.021 | This study titled 'Depressive symptoms in Taiwanese women during the peri- and post-menopause years: Associations with demographic, health, and psychosocial characteristics ' was excluded due to exposure/outcome categories. Therefore, no specific symptom-related findings were extracted. |
| Wang 2021 | lack of symptom-specific statistics | https://doi.org/10.1186/s12905-021-01531-x | Wang et al. (2021) found that nearly half of the women (49.27%) experienced moderate or severe menopausal symptoms, with perimenopausal and postmenopausal women reporting significantly higher symptom scores than premenopausal women. Symptom severity was positively associated with older age, higher BMI, non‑married status, longer menstruation duration, higher parity, extended breastfeeding, and menopausal status, as well as use of menopausal hormone therapy. In contrast, higher income, education, physical activity, and a history of gynecologic, breast, or chronic disease were linked to lower symptom scores. Overall, the study highlights a broad set of demographic and reproductive factors contributing to greater symptom severity among Chinese women. |
| Wang 2022 | exposure/outcome categories | https://doi.org/10.3389/fpsyg.2022.763596 | This study titled 'Menopause-Related Symptoms and Influencing Factors in Mosuo, Yi, and Han Middle-Aged Women in China ' was excluded due to exposure/outcome categories. Therefore, no specific symptom-related findings were extracted. |
| Wang 2022_2 | exposure/outcome categories | https://doi.org/10.1080/13697137.2021.1998435 | This study titled 'Association of menopausal status and symptoms with depressive symptoms in middle-aged Chinese women ' was excluded due to exposure/outcome categories. Therefore, no specific symptom-related findings were extracted. |
| Watson 2019 | exposure/outcome categories | https://doi.org/10.1097/LGT.0000000000000460 | This study titled 'Vulvovaginal Discomfort Is Common in Both Premenopausal and Postmenopausal Women ' was excluded due to exposure/outcome categories. Therefore, no specific symptom-related findings were extracted. |
| Whiteley 2013 | exposure/outcome categories | 10.1089/jwh.2012.3719 | This study titled 'The Impact of Menopausal Symptoms on Quality of Life, Productivity and Economic Outcomes ' was excluded due to exposure/outcome categories. Therefore, no specific symptom-related findings were extracted. |
| Williams 2009 | lack of symptom-specific statistics | https://dx.doi.org/10.1016/j.maturitas.2008.12.006 | Williams et al. (2009) reported that menopausal symptoms had a substantial negative impact on multiple domains of health‑related quality of life, with vasomotor, psychosocial, physical, and sexual MENQOL scores all showing meaningful symptom burdens. Hot flushes were particularly disruptive, affecting sleep (82%), mood (68.6%), concentration (69%), work (46%), and overall quality of life (69.3%). Symptom severity varied by age, education, BMI, exercise, and smoking status, indicating that sociodemographic and lifestyle factors significantly shaped menopause‑related quality‑of‑life outcomes. |
| Wong 2014 | exposure/outcome categories | 10.1371/journal.pone.0110877 | This study titled 'Prevalence and psychosocial correlates of depressive symptoms in urban Chinese women during midlife ' was excluded due to exposure/outcome categories. Therefore, no specific symptom-related findings were extracted. |
| Woods 2022 | exposure/outcome categories | https://doi.org/10.1097/GME.0000000000002072 | This study titled 'Perimenopause meets life: observations from the Women Living Better Survey ' was excluded due to exposure/outcome categories. Therefore, no specific symptom-related findings were extracted. |
| Worsley 2017 | exposure/outcome categories | https://doi.org/10.1089/jwh.2016.6142 | This study titled 'Moderate–Severe Vasomotor Symptoms Are Associated with Moderate–Severe Depressive Symptoms ' was excluded due to exposure/outcome categories. Therefore, no specific symptom-related findings were extracted. |
| Wu 2023 | lack of symptom-specific statistics | https://doi.org/10.1186/s12905-023-02347-7 | Wu et al. (2023) found that 74.8% of midlife women reported menopausal symptoms, with over half experiencing moderate to severe symptom levels. The study showed a strong inverse association between physical activity and symptom severity—women with higher activity levels had significantly fewer or milder symptoms. Factors such as perimenopausal or postmenopausal status, poorer perceived health, and higher parity were associated with greater symptom severity, while physical activity emerged as the most influential protective factor. |
| Xiong 2022 | lack of symptom-specific statistics | https://doi.org/10.1016/j.sleep.2022.08.025 | Xiong et al. (2022) examined sleep in midlife women, but no symptom‑specific statistics or detailed results are accessible through indexed databases or public abstracts. Because the article’s methods, outcomes, and effect estimates cannot be retrieved from any publicly available source, associations between menopausal symptoms and sleep outcomes cannot be evaluated. As a result, the study provides insufficient extractable data for symptom‑specific synthesis and is excluded from comparative analysis. |
| Yang 2008 | lack of symptom-specific statistics | https://doi.org/10.1080/13697130802239075 | Yang et al. (2008) found that mid‑life women in southern China reported a generally low prevalence and low severity of menopausal symptoms, with insomnia, joint/muscle pain, and dizziness being the most common complaints. Only 17.5% experienced hot flushes, a much lower rate than typically reported in Western cohorts. Symptom frequency was associated with profession, education level, type of menopause, and the presence of physical or emotional health problems. Overall, care‑seeking was limited, with fewer than one‑third of symptomatic women consulting healthcare providers. |
| Yanikkerem 2018 | exposure/outcome categories | https://doi.org/10.1080/13697137.2017.1417374 | This study titled 'Effects of physical and depressive symptoms on the sexual life of Turkish women in the climacteric period ' was excluded due to exposure/outcome categories. Therefore, no specific symptom-related findings were extracted. |
| Yim 2014 | lack of symptom-specific statistics | https://scholarx.skku.edu/handle/2021.sw.skku/53368 | This study titled 'Menopausal symptoms and associated factors in middle-aged women in Korea ' was excluded due to lack of symptom-specific statistics. Therefore, no specific symptom-related findings were extracted. |
| Yim 2015 | exposure/outcome categories | http://dx.doi.org/10.1097/GME.0000000000000438 | This study titled 'Prevalence and severity of menopause symptomsand associated factors across menopause status in Korean women ' was excluded due to exposure/outcome categories. Therefore, no specific symptom-related findings were extracted. |
| Yin 2015 | exposure/outcome categories |  | This study titled 'Association between polycystic ovary syndrome and hot flash presentation during the menopause transition ' was excluded due to exposure/outcome categories. Therefore, no specific symptom-related findings were extracted. |
| Yokota 2016 | incompatible reference groups | https://doi.org/10.1097/GME.0000000000000660 | Yokota et al. (2016) found that general fatigue and shoulder stiffness were the most common symptoms among 1,969 Japanese women. Vasomotor symptoms, including hot flushes and sweating, differed significantly between pre‑ and postmenopausal women. Symptom severity also varied by BMI, with overweight women reporting more severe vasomotor, joint, and urinary symptoms, while underweight women experienced more cold intolerance, nervousness, and skin changes. Overall, the study identifies BMI as an important factor shaping menopausal symptom patterns. |
| Yoshany 2020 | exposure/outcome categories | https://doi.org/10.29333/ejgm/7885 | This study titled 'Association between Lifestyle and Severity of Menopausal Symptoms in Postmenopausal Women ' was excluded due to exposure/outcome categories. Therefore, no specific symptom-related findings were extracted. |
| Zainal 2008 | exposure/outcome categories | http://dx.doi.org/10.1177/1010539508322538 | This study titled ' Depressive Symptoms in Middle-Aged Women in Peninsular Malaysia ' was excluded due to exposure/outcome categories. Therefore, no specific symptom-related findings were extracted. |
| Zeleke 2016 | lack of symptom-specific statistics | http://dx.doi.org/10.1016/j.fertnstert.2015.09.017 | Zeleke et al. (2016) found that one‑third of older Australian women (32.8%) who were not using menopausal hormone therapy continued to experience vasomotor symptoms such as hot flushes and night sweats. Although only 3.4% rated these symptoms as moderately to severely bothersome, sexual symptoms were highly prevalent, affecting more than half of partnered women, with 32.5% reporting vaginal dryness during intercourse. Factors associated with ongoing vasomotor symptoms included older age, obesity, caregiving responsibilities, and prior bilateral oophorectomy. Overall, the study highlights that untreated estrogen‑deficiency symptoms remain common well into older age. |
| Zeleke 2016 | lack of symptom-specific statistics | DOI: 10.1080/13697137.2016.1242242 | Zeleke et al. (2016) found that vasomotor symptoms (VMS) persisted in nearly one‑third (32.8%) of community‑dwelling Australian women aged 65–79, even decades after menopause. Obesity, caregiving responsibilities, and bilateral oophorectomy were significantly associated with ongoing VMS, while vulvovaginal atrophy (VVA) and pelvic floor disorders were also common, affecting 20.3% and 47.2% of women respectively. The study highlights that estrogen‑deficiency symptoms often continue into older age, and clinicians should remain attentive to persistent VMS, VVA, and overlapping pelvic floor problems in this population. |
| Zeleke 2017 | exposure/outcome categories | DOI: 10.1097/GME.0000000000000938 | This study titled 'Vasomotor symptoms are associated with depressive symptoms in community-dwelling older women ' was excluded due to exposure/outcome categories. Therefore, no specific symptom-related findings were extracted. |
| Zhang 2016 | lack of symptom-specific statistics | DOI: 10.4103/0366-6999.178961 | Zhang et al. (2016) reported that nearly half of women (49.8%) experienced menopausal symptoms, with the highest prevalence occurring during the early postmenopausal stage. The five most common symptoms were fatigue, joint/muscle pain, decreased libido, insomnia, and nervousness, reflecting a broad mix of physical and psychological complaints. Additionally, poor sleep quality was highly prevalent, affecting 55% of perimenopausal women. Overall, symptoms were common but generally mild, yet still contributed notably to reduced well‑being during the menopausal transition. |
| Zhao 2021 | lack of symptom-specific statistics | https://doi.org/10.1097/GME.0000000000001864 | Zhao et al. (2021) used latent class analysis to identify four distinct menopausal symptom subtypes among 797 Chinese women: severe symptoms, dominant sleep–emotion symptoms, physical/mental exhaustion, and no symptoms. These symptom patterns were stable across age and menopause‑status subgroups, indicating robust underlying clusters. Sociodemographic and psychosocial factors—such as education, health status, and emotional well‑being—were significantly associated with class membership, highlighting their role in shaping symptom experiences. |
| Zhao 2022 | lack of symptom-specific statistics | http://dx.doi.org/10.11836/JEOM21380 | Zhao et al. (2022) found that perimenopausal symptoms were highly prevalent among female petrochemical workers, affecting nearly half of the sample. Key factors associated with higher symptom scores included psychological stress, occupational exposures, reproductive history, and demographic characteristics, highlighting the combined impact of workplace and personal factors. The authors developed a risk nomogram that integrated these predictors to support early identification and monitoring of women at elevated risk for significant perimenopausal symptoms. |
| ZhongKai 2015 | lack of symptom-specific statistics | DOI: 10.1097/GME.0000000000001850 | This study titled 'Survey of common symptoms of peri menopausal women in Shenzhen city and their influencing factors. ' was excluded due to lack of symptom-specific statistics  . Therefore, no specific symptom-related findings were extracted. |
| Zhou 2018 | exposure/outcome categories | https://doi.org/10.1080/13697137.2018.1428295 | This study titled 'Association of body composition with menopausal symptoms in (peri-)menopausal women. ' was excluded due to exposure/outcome categories . Therefore, no specific symptom-related findings were extracted. |
| vanPoppel 2008 | exposure/outcome categories | https://doi.org/10.1097/gme.0b013e31804b418c | This study titled '"It's my hormones, doctor"-does physical activity help with menopausal symptoms?' was excluded due to exposure/outcome categories . Therefore, no specific symptom-related findings were extracted. |
