# Supplementary material for: Factors Associated With Menopause Symptoms: A Systematic Review and Meta‐Analysis
Source: BJOG. 2026 May 6;133(11):1967–85. doi: 10.1111/1471-0528.70257 (PMC13571893; doi:10.1111/1471-0528.70257)
Supplement: Supplementary file 2 [file BJO-133-1967-s002.docx]

| Corresponding Author | Responsibilities |
| --- | --- |
| First CA: Janice Hoang  j.hoang@leeds.ac.uk | Prepare a submission-ready manuscript with all necessary auxiliary files.  Put together a submission package, stay in close contact with the publisher, and follow time schedules.  Ensure authorship details are correct, have all authors review and approve the final version, get signed consents.  Obtain permissions for the use of copyrighted materials, follow all editorial and submission policies, adhere to ethical practices and guidelines.  Manage open access details, deal with the payment and invoicing, meet specific institutional or funder requirements, add the article to a repository. |
| Second CA: Jennifer Hirst  [jennifer.hirst@phc.ox.ac.uk](mailto:jennifer.hirst@phc.ox.ac.uk) | Forward readers’ scientific/copyright-related questions to the co-authors, reply with the collective response.  Manage requests for data sharing. |
